# Assessing Best Practices in Natural Gas Production and Emerging CO_2_ Capture Techniques to Minimize the Carbon Footprint of Electricity Generation

**DOI:** 10.1021/acs.est.4c02933

**Published:** 2024-11-16

**Authors:** Ryan Cownden, Mathieu Lucquiaud

**Affiliations:** Department of Mechanical Engineering, University of Sheffield, Sheffield S1 3JD, U.K.

**Keywords:** life cycle assessment, greenhouse gas emissions, combined cycle gas turbines, duty cycle, carbon capture and storage, natural gas production

## Abstract

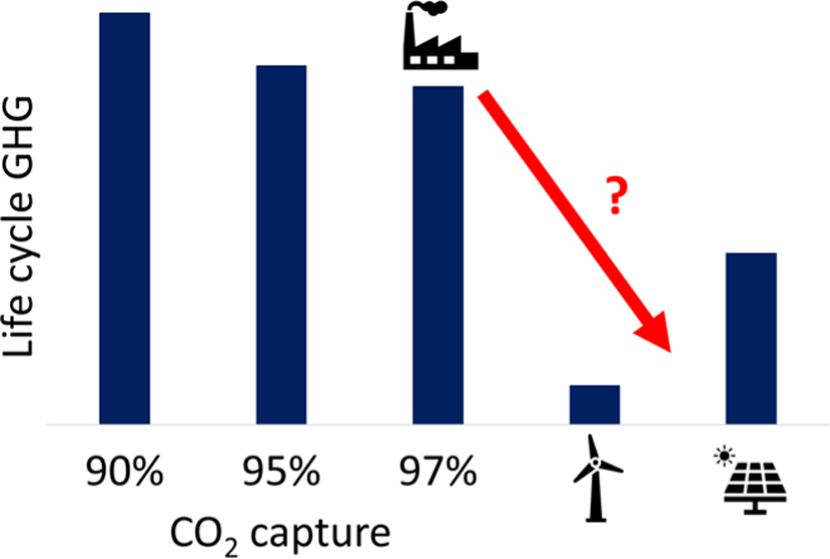

Natural gas (NG) is expected to provide a substantial portion of electricity generation in many jurisdictions for the foreseeable future. Postcombustion carbon capture and storage (CCS) effectively abates direct CO_2_ emissions; however, indirect NG supply chain emissions in most jurisdictions are incompatible with climate change mitigation goals. This life cycle assessment evaluates specific opportunities to reduce the carbon footprint of combined cycle gas turbine (CCGT) generation with CCS using existing low-emission NG production practices, technologies, and processes combined with emerging CCS techniques to achieve high CO_2_ capture rates and mitigate startup emissions. We find baseload life cycle greenhouse gas (GHG) emission intensity ranges from 22 to 62 kgCO_2_e/MWh for 95–98.5% CO_2_ capture, within the range of published estimates for wind and photovoltaic power and considerably below prior estimates of CCGT with CCS. Low-emission NG production practices reduce other environmental impacts, which are dominated by combustion-related air pollution. We also show how interim solvent storage can effectively mitigate emissions from CCGT start/stop cycles. This work highlights the importance of mitigating both CO_2_ and methane emissions from NG supply chains and proposes a more nuanced discussion regarding the potential contribution of NG to the future energy supply. A surrogate model is provided to estimate life cycle GHG emissions for CCGT with CCS and user-input parameters.

## Abstract 2

First LCA of electricity generation from natural gas with best practices in supply chain emissions; shows carbon footprint could be comparable to wind turbines and 63–71% lower than photovoltaics.

## Introduction

Transitioning to low-carbon energy sources is critical for efforts to mitigate climate change because production and consumption of energy creates 65% of anthropogenic greenhouse gas (GHG) emissions.^1−3^ However, jurisdictions and individuals approach proposed changes to energy systems from different perspectives and ideological worldviews.^4^ There is also substantial regional variability in existing energy sources,^5^ forecast demand,^6^ temporal consumption patterns,^7^ available renewable resources,^8^ and fossil fuel reserves.^3^ Cost, political, fiscal, and energy security considerations are expected to lead to heterogeneous development of low-carbon energy sources.^8^

Worldwide wind and photovoltaic power generation have both grown substantially since the Paris Agreement was signed (+4.6 and +3.8 EJ, respectively, in 2022 compared to 2015), yet fossil fuel consumption has also increased: +19.3 EJ for natural gas (NG), +7.2 EJ for oil, and +4.3 EJ for coal.^9^ While costs for wind and photovoltaic electricity generation have decreased considerably, balancing generation and demand becomes more challenging and costly as the share of intermittent renewable generation increases.^10^ Electricity production from fossil fuels can provide important dispatchable generation and inertial services for power grids.^11^ Based on current government energy policies, fossil fuels are expected to continue supplying most human energy consumption over the next 30 years (60–80% in 2050^12,13^), but the associated GHG emission forecasts are inconsistent with commitments to mitigate climate change. Limiting global warming (GW) within the Paris Agreement goals would require faster growth in low-carbon energy than is currently forecast.^13^

State-of-the-art combined cycle gas turbines (CCGTs) generate electricity with direct CO_2_ emissions of approximately 334 kgCO_2_/MWh^14^ and also cause substantial indirect GHG emissions and other environmental impacts.^15−17^ Postcombustion carbon capture and storage (CCS) can effectively mitigate most direct emissions and is technologically ready for widespread deployment;^10,14,18^ however, CCS increases other environmental impact intensities, primarily due to higher NG consumption.^15−17^ Similarly, wind and photovoltaic power installations generate electricity with no direct emissions but impact the environment through their supply chains and land occupation.^10,16,17^ Life cycle assessment (LCA) is an effective tool to evaluate and compare the emissions and impacts caused by electricity generation technologies at all stages of production.^10,16^

Fuel consumption and flaring during NG production impact not only GHG emissions but also many other environmental impact categories due to nitrogen oxide (NOx) and sulfur dioxide pollution.^15^ A large proportion of stationary combustion emissions during NG production come from NG engine-driven compressors.^15,19^ Reducing NOx emissions from NG engines typically leads to increased methane emissions.^20^

Prior LCAs of CCGT electricity generation with CCS have been based on national or continental average NG supply (e.g., refs (15–17 and 21–32)); however, indirect emissions/impacts vary widely between jurisdictions due to different production practices and regulations (e.g., 4.2–14 gCO_2_e/MJ in ref (33)). Average emissions are not representative of best practices in jurisdictions with low-emission NG production.^33,34^ Bui et al. performed a parametric analysis showing that life cycle emissions of CCGT’s with CCS are strongly dependent on the GHG emission intensity of the NG supply;^35^ however, their analysis did not assess NG supply chains to determine what is technically feasible and did not consider other environmental impacts.

Fugitive methane from NG supply chains can substantially affect life cycle GHG emissions of CCGT with CCS, but leakage rates vary widely between jurisdictions (e.g., near zero in Norway compared to >6% in Libya and Iraq^36^) and between comparable facilities/regions within the same jurisdiction.^37−39^ Government regulations and leak detection/repair programmes have successfully reduced fugitive methane rates in some jurisdictions.^38,40^ Fugitive methane emission intensity for NG production in British Columbia, Canada (BC), dropped 81% from 2006 to 2021 (absolute reduction of 59%)^41,42^ (Supporting Information Figure 1) as methane emission regulations became progressively more restrictive.^43^ In 2021, the BC government established new regulations for NG production^44^ as part of its plan to reduce methane emissions from the BC oil and NG industry to 75% below 2014 emissions by 2030 and near zero by 2035.^45,46^ Similar regulations apply to other Canadian provinces,^46^ and there is a broad effort by many countries to abate methane emissions (e.g., refs (47 and 48)).

Many studies in different jurisdictions have measured methane emission rates from oil and NG production facilities that are higher than implied by government-reported estimates, but recent changes to reporting standards in Canada have aligned reported emissions with independent estimates.^49^ The BC government substantially increased fugitive methane emission estimates (current and historical) for the oil and NG industry in their 2020 and 2021 GHG emission inventories.^42,50^ Satellite measurements during May 2018 to February 2020 published in ref (39) for northeastern BC where upstream oil and gas production is located indicate annual methane emissions 14% lower than the 2019 methane emission estimates reported by the BC government for the upstream oil and NG industry (Supporting Information Note 1.5.1).

Most prior LCAs of CCGT with CCS have also assumed CO_2_ capture rates of 90% or less (e.g., refs (15–17 and 21–32)); however, capture rates up to 99% have been found feasible with relatively little impact on cost^14,51−54^ and demonstrated in pilot testing.^18,55−57^ Gross-CO_2_ capture of 99.2% for CCGT captures 100% of the fossil-CO_2_ associated with NG combustion after discounting CO_2_ entering with ambient air. State-of-the-art baseline performance studies by the US National Energy Technology Laboratory (NETL)^14^ and the International Energy Agency^58^ identify gross-CO_2_ capture rates for CCGT up to 97% and 98.5%, respectively, as feasible, but do not consider supply chain emissions. Evidence from peer-reviewed science formed the basis of recent requirements for the permitting of postcombustion CO_2_ capture plants in the UK.^59^ The guidance directs project proponents to design plants to achieve and demonstrate a minimum CO_2_ capture rate of 95% under normal operating conditions as part of the environmental permitting process and subsequent operations. Similarly, the draft Canadian Clean Electricity Regulation requires fossil-fueled electricity generators to achieve an annual average CO_2_ emission intensity of less than 30 kg/MWh including starts/shutdowns by 2035, based on an assumed annual average CO_2_ capture rate of 95%.^60^ Recent assessments of life cycle GHG emissions from CCGTs with up to 100% fossil-CO_2_ capture^35,54^ used simplified life cycle inventories which do not comply with best practices for LCA, did not consider other environmental impacts, and did not rigorously assess opportunities to reduce upstream emissions from NG supply to show how to achieve low life cycle GHG emission intensity in practice.

Existing CCGT facilities in different jurisdictions operate with a wide range of different load profiles and duty cycles.^61,62^ The proportion of electricity generated by intermittent wind and photovoltaic facilities is expected to increase considerably in most jurisdictions^6^ and CCGT facilities will be required to operate more flexibly to balance supply and demand. Several studies have assessed the economic implications for CCGT power generation.^11^ Further, the effect of part-load operation and transients on performance of CCGTs with CCS have been assessed.^11,63,64^ However, existing LCAs of CCGT with CCS only consider baseload operation at rated output (e.g., refs (15–17, 21–25, and 27–31)).

Postcombustion CO_2_ capture is typically performed using a regenerative amine absorption process—amine solvent absorbs CO_2_ from the exhaust gas stream at low temperature and is regenerated by heating the CO_2_-rich amine to release the CO_2_. Low-pressure steam is usually used to regenerate the amine.^14^ However, steam is unavailable during the initial stages of CCGT startup, while the heat recovery steam generator warms up to operating temperature and this can increase CO_2_ emissions during start cycles.^35^ CO_2_ emissions during startup can be mitigated by temporarily storing CO_2_-rich solvent while the system heats up.^65^ Storing CO_2_-rich solvent during startup requires additional solvent capacity and the stored solvent needs to be regenerated once the system reaches operating temperature with an associated energy penalty.^11^ Bui et al.^35^ included solvent storage in assessing the effect of startup/shutdown cycles on carbon footprint of CCGT generation. However, their analysis was based on empirical data from testing solvent storage on one pilot plant which did not have equipment designed with the intent to minimize startup/shutdown emissions. Limited storage capacity and the existing process configuration reduced total CO_2_ capture during startup compared to a purpose-built system designed with sufficient interim solvent storage to fully abate emissions prior to the system reaching operating temperature.

In this study, we perform a detailed LCA of low-emission NG production practices to identify how life cycle GHG emissions from CCGTs could be reduced to align with climate change mitigation goals. We develop a detailed inventory of existing low-emission NG production practices in BC and evaluate three specific opportunities to further mitigate GHG emissions: low-emission processing plant design, electrification of compressor drivers, and achieving the BC government’s 2030 fugitive methane emission reduction target. We combine this novel low-emission NG supply chain with CCGT using CCS to calculate full life cycle environmental impacts up to the point of producing a carbon-free energy vector (electricity) and evaluate CO_2_ capture rates >95% consistent with the emerging regulatory framework for fossil fuel power generation in the UK and Canada. We extend the analysis by investigating the effect of duty cycles and startup/shutdown cycles on life cycle impacts and the opportunity to store solvent during startup to mitigate CO_2_ emissions.

## Methods

**Figure 1:**
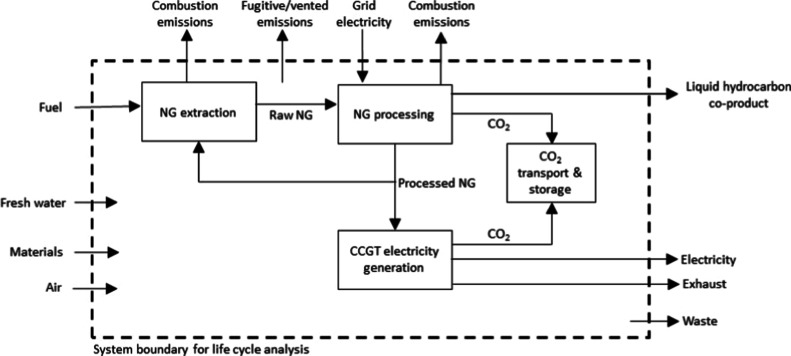
Simplified system boundary for LCA calculations.

This cradle-to-gate LCA evaluated electricity generated by a new industrial-scale CCGT with CCS supplied with NG from low-emission production practices. Life cycle impacts for net electricity generated (functional unit of 1 MWh output at 345 kV to transmission grid) were calculated for the system boundary (Figure 1) in accordance with ISO 14040/14044.^66,67^ 100 year global warming potential (GWP) was used to characterize CO_2_ equivalence of GHG species for consistency with most prior studies and alignment with UNFCCC GHG reporting. We used process data to capture the most important contributions to life cycle impacts (e.g., direct emissions and primary material consumption) and environmentally extended input–output factors to account for small contributions where process data were not available (e.g., construction activities for the CO_2_ capture plant and the NG processing plant). Similar hybrid LCA methodology has been used previously to study CCGT with CCS.^21,22^ Life cycle impacts were assessed with SimaPro^68^ using the Ecoinvent database^69^ for background inventory and the ReCiPe 2016 impact assessment method^70^ based on midpoint indicators. GWP characterization factors were updated to align with IPCC assessment report 6.^71^ Details on the LCA methodology and inventories summarized below are available in Supporting Information Note 1.

### NG Extraction Inventory

We used public data from the BC Energy Regulator^19,72,73^ to estimate average material and energy inputs to drill, complete, and equip Montney formation NG wells in 2020 and associated hydrocarbon production. The Montney is a large shale formation in northeast BC with substantial remaining NG reserves and well inventory^74,75^ and accounted for 91% of new oil and NG wells drilled in BC in 2020 (348 of 384).^19^ BC is advantageous for this case study because there is low liquid hydrocarbon production compared to other jurisdictions which reduces the significance of coproduct allocation to the results. We selected 2020 as the period for this analysis to provide a balance between using recent data while ensuring sufficient historical production to accurately estimate well productivity.

The NG extraction inventory includes direct and indirect emissions from land use, drilling, completions, wellbore materials, surface equipment, gathering pipelines, and access roads.

### NG Production Inventory

The NG production inventory includes direct and indirect emissions associated with operating the wells and processing the raw well effluent. We considered four scenarios for NG production practices. The first scenario assumed average emissions for BC NG production based on existing infrastructure and practices (total of 5.44 gCO_2_e/MJ_LHV_). This scenario is representative of a CCGT supplied from the NG transmission pipeline in northeast BC.

For the second scenario, we modeled a NG production process designed to minimize emissions while meeting typical NG and condensate product specifications using Aspen Hysys^76^ (detailed description in Supporting Information Note 2). The low-emission design eliminated process CO_2_ venting and assumed electricity supply from the BC grid and NG engine-driven compressors meeting current government emission standards. The resulting energy allocation between NG and liquid coproduct for the low-emission processing plant design matches the reported marketable hydrocarbon production in BC^41^ to 3 significant digits (90.2% NG). This scenario assumed the BC average fugitive methane emissions from NG production in 2020 (1.06 gCO_2_e/MJ_LHV_).

The third and fourth scenarios used the same NG production process, but NG compressors were assumed to be electric motor driven. While most existing compressors in BC are NG engine driven, some facilities located near grid distribution use industrial-scale electric motors and the BC electric utility has a mandate to increase the use of grid-supplied electricity by the NG industry.^77^ Impacts associated with electricity consumption were modeled based on medium voltage electricity supply for BC in the Ecoinvent database (78 kgCO_2_e/MWh). The third scenario used BC average fugitive emissions in 2020, while the fourth scenario assumed that fugitive methane emission intensity is reduced to 0.51 gCO_2_e/MJ_LHV_ (75% of 2014 intensity in BC), consistent with BC government policy^45^ assuming constant NG production.

Emissions to air from flaring during NG production for all scenarios were based on BC industry average data in 2020 (0.20 gCO_2_/MJ_LHV_ plus unburnt methane included in the fugitive emissions).

### CCGT Inventory

The most recent US NETL state-of-the-art power generation baseline study^14^ provides H-class CCGT (873–883 MW_e_) operating inventories for three CO_2_ capture rates (90%, 95%, and 97% gross). These gross-CO_2_ capture rates correspond to 90.7%, 95.7%, and 97.7% fossil-CO_2_ capture. The IEAGHG baseline study^58^ includes analysis of a similar CCGT with 98.5% gross-CO_2_ capture (99.2% fossil-CO_2_) using the same absorption solvent as the NETL study (Cansolv). The ratio of net power output at 98.5% capture to 90% capture in the IEAGHG study was used to estimate the net power output for the CCGT design in the NETL study at 98.5% capture (864 MW_e_). Results for CO_2_ capture rates greater than 95% are provided in the main text, while results for 90% are included in Supporting Information Note 3 to facilitate comparisons with legacy studies of CCGT with CCS. Solvent consumption rates and life cycle inventory for the proprietary solvent assumed in the NETL and IEAGHG studies are not publicly available, so we used data for monoethanolamine as a proxy. CCGT with CCS using monoethanolamine solvent can achieve similar CO_2_ capture rates and CCGT efficiency as published in the NETL and IEAGHG studies.^54^

We combined typical CCGT startup (cold, warm, and hot) and shutdown procedures^65,78^ with estimates of part-load performance for CCGT with CCS^11^ to determine emissions and NG consumption for different operating modes. We assessed the effect of operating profiles based on data (2020–2022) from five CCGT facilities in different North American jurisdictions with units that have rated power outputs similar to those of the CCGT in this study. Capacity factors and duty cycles for these units range from 37 to 81% and 0.3–104 shutdowns per year.^61,62^ We also considered theoretical future duty cycles with up to 400 startup/shutdown cycles per year. We compared the effect of unmitigated CO_2_ emissions during startup with abatement using a process design with sufficient interim solvent storage capacity to mitigate CO_2_ emissions throughout the startup sequence until the regenerator reached the operating temperature. Stored CO_2_-rich solvent was regenerated at part load after the CCGT reached operating temperature.

### CO_2_ Sequestration

Infrastructure requirements for CO_2_ sequestration are difficult to generalize because of considerable variability in subsurface geology.^79^ Many potential target formations for CO_2_ disposal have been identified in northeast BC—depleted hydrocarbon pools and widespread saline aquifers—with storage potential c. two orders of magnitude larger than the c. 75 MtCO_2_ required for 30 years of baseload operation of the CCGT in this study.^80^ There is substantial variability in porosity, permeability, and thickness between, and within, potential disposal formations.^80^ We assumed a total of five disposal wells to sequester CO_2_ based on an assumed maximum injectivity rate of c. 0.6 MtCO_2_/year per well similar to injectivity demonstrated at an existing CCS project in Alberta, Canada.^81^ We assumed a total of 50 km of 323 mm diameter pipeline to access different disposal pools and/or distribute CO_2_ disposal within the aquifer.

### Life Cycle Impact Comparisons

We include comparisons with the life cycle impact assessment to provide context for the results: CCGT with CCS assuming global/UK average NG supply and photovoltaic/wind generation in BC and western USA. Western USA is relevant in this context because it is part of the same North American electric grid interconnection (Western Interconnection) as BC.

## RESULTS AND DISCUSSSION

### NG Supply Chain Impacts

**Figure 2:**
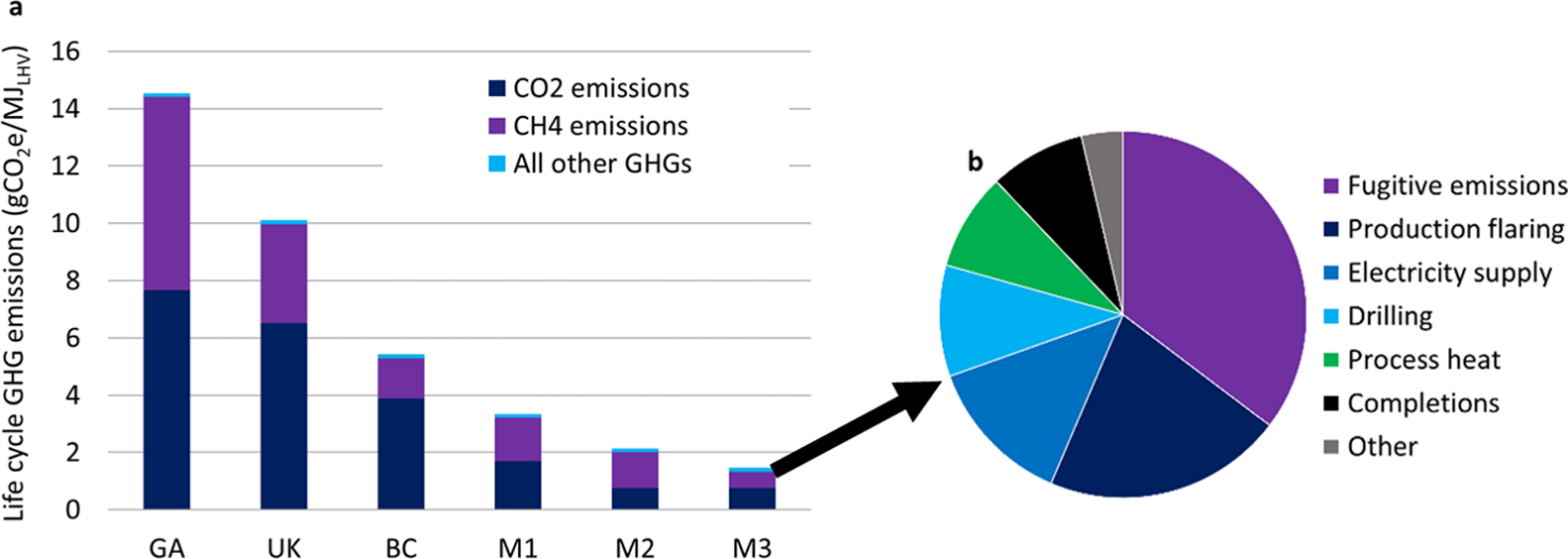
Life cycle GHG emission intensity of NG production practices. (a) Carbon footprint with species contributions for global average NG supply (GA), UK average NG supply (UK), BC average production of marketable NG (BC), and the three low-emission cases developed in this study for BC Montney production with NG drive compressors (M1), electric drive compressors (M2), and electric drive compressors with reduced fugitive emissions (M3). (b) Breakdown of carbon footprint for the M3 scenario. “Other” includes NG processing plant construction and maintenance, land use (LU) change, wellsite surface equipment, pipelines, roads, and decommissioning.

GHG emission intensity of upstream NG production in BC (5.44 gCO_2_e/MJ_LHV_) is 64% lower than the Ecoinvent global average NG supply (14.5 gCO_2_e/MJ_LHV_) and 46% lower than the UK average NG supply (10.1 gCO_2_e/MJ_LHV_ including imported NG) reflecting low fugitive methane emission intensity in BC and low CO_2_ emission intensity due to the absence of long-distance transportation (Figure 2a). Other jurisdictions with similar low-emission domestic NG production include offshore UK (4.76 gCO_2_e/MJ_LHV_), Norway (2.57), and Qatar (6.06).^68^ Low-emission processing plant design with Montney NG wells and an electricity supply from the BC grid would reduce GHG emission intensity to 3.35 gCO_2_e/MJ_LHV_ with NG-drive compressors and 2.14 gCO_2_e/MJ_LHV_ with electric-drive compressors. Achieving fugitive methane emission intensity consistent with the BC 2030 target would reduce the GW intensity to 1.46 gCO_2_e/MJ_LHV_ with electric compressors. The largest contributions to life cycle GHG emissions in the lowest emission scenario are residual fugitive methane (35%), production flaring (21%), and grid-supplied electricity (13%) (Figure 2b).

The absence of energy consumption and infrastructure for long-distance transportation in the BC NG supply scenarios also substantially reduces most other life cycle environmental impacts compared to the global and UK average NG supply (Supporting Information Figure 20). Stratospheric ozone depletion in the BC NG supply scenarios is higher than the global average because of nitrous oxide emissions from flaring, which are not included in the Ecoinvent methodology. Stratospheric ozone depletion in the UK average NG supply scenario is also higher than the global average because of emissions of ozone depleting chemicals associated with firefighting equipment for offshore production operations, which account for a larger share of UK supply than the global average.^68,82^ Water consumption in the electric-drive compressor supply scenarios is higher than the global average because of the predominance of hydroelectric power generation in the BC electricity supply.

### Baseload CCGT Scenarios

**Figure 3:**
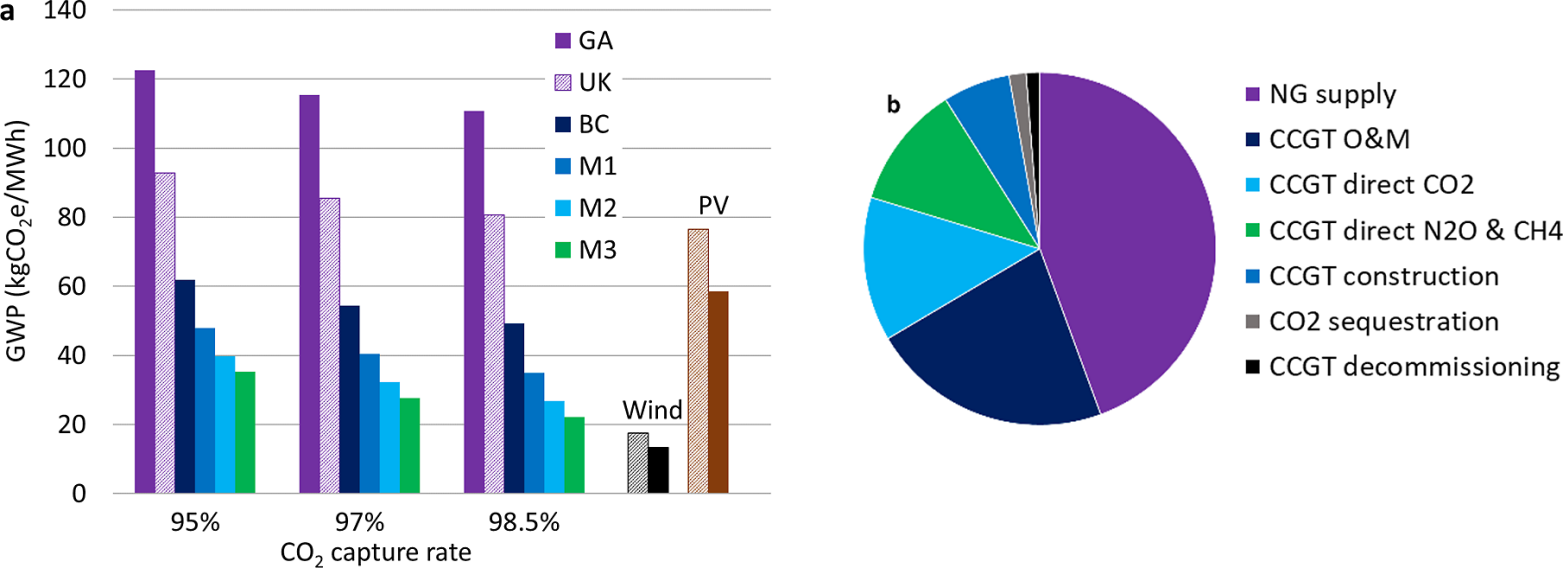
Life cycle GW intensity of electricity produced from CCGT with CCS. (a) Carbon footprint for six NG supply chain scenarios compared to wind and photovoltaic generation. NG supply scenarios: global average supply (GA), UK average supply (UK), BC average production in 2020 (BC), BC Montney production with NG drive compressors (M1), BC Montney production with electric drive compressors (M2), and BC Montney production with electric drive compressors and 2030 fugitive methane emission reduction target achieved (M3). Results for wind and photovoltaic shown for BC (diagonal hatch) and western USA (solid). (b) Breakdown of life cycle GHG emissions for CCGT with 98.5% CO_2_ capture and BC Montney NG supply with electric drive compression and reduced fugitive methane emissions (M3).

Life cycle GW intensity for baseload electricity generation is more affected by NG production practices than the CO_2_ capture rate over the range of scenarios considered in this study (Figure 3a). Increasing the CO_2_ capture rate from 95% to 98.5% reduces life cycle GW intensity 13 kgCO_2_e/MWh versus a reduction of 61 kgCO_2_e/MWh using BC average NG production compared to global average NG supply. All three low-emission NG production scenarios that were assessed materially reduce GHG emission intensity. Eliminating CO_2_ venting and achieving the 2030 fugitive methane emission target would reduce life cycle GW intensity by 4 and 5 kgCO_2_e/MWh, respectively, compared to BC average NG production. The remainder of the emission reductions are due to lower fuel gas consumption during NG processing (e.g., replacing self-generated electricity with low-carbon grid electricity and c. 8 kgCO_2_e/MWh from electrifying compression). Emission intensity of electricity from CCGT with CCS using BC average NG production (49–62 kgCO_2_e/MWh for 95–98.5% CO_2_ capture) is slightly lower than photovoltaic power generation in BC and western USA (59–77 kgCO_2_e/MWh), while CCGT with 98.5% CO_2_ capture supplied with the lowest emission NG scenario (22 kgCO_2_e/MWh) approaches the range of wind power (13–18 kgCO_2_e/MWh).

In the lowest CCGT emission scenario, upstream NG supply accounts for 44% of life cycle GHG emissions with 22% from CCGT operations and maintenance (50% of which is makeup amine solvent) and 13% from CCGT residual direct CO_2_ emissions (Figure 3b). At 98.5% CO_2_ capture, residual CO_2_ emissions contribute approximately the same share of life cycle GHG emissions as direct nitrous oxide and methane emissions from the CCGT.

**Figure 4:**
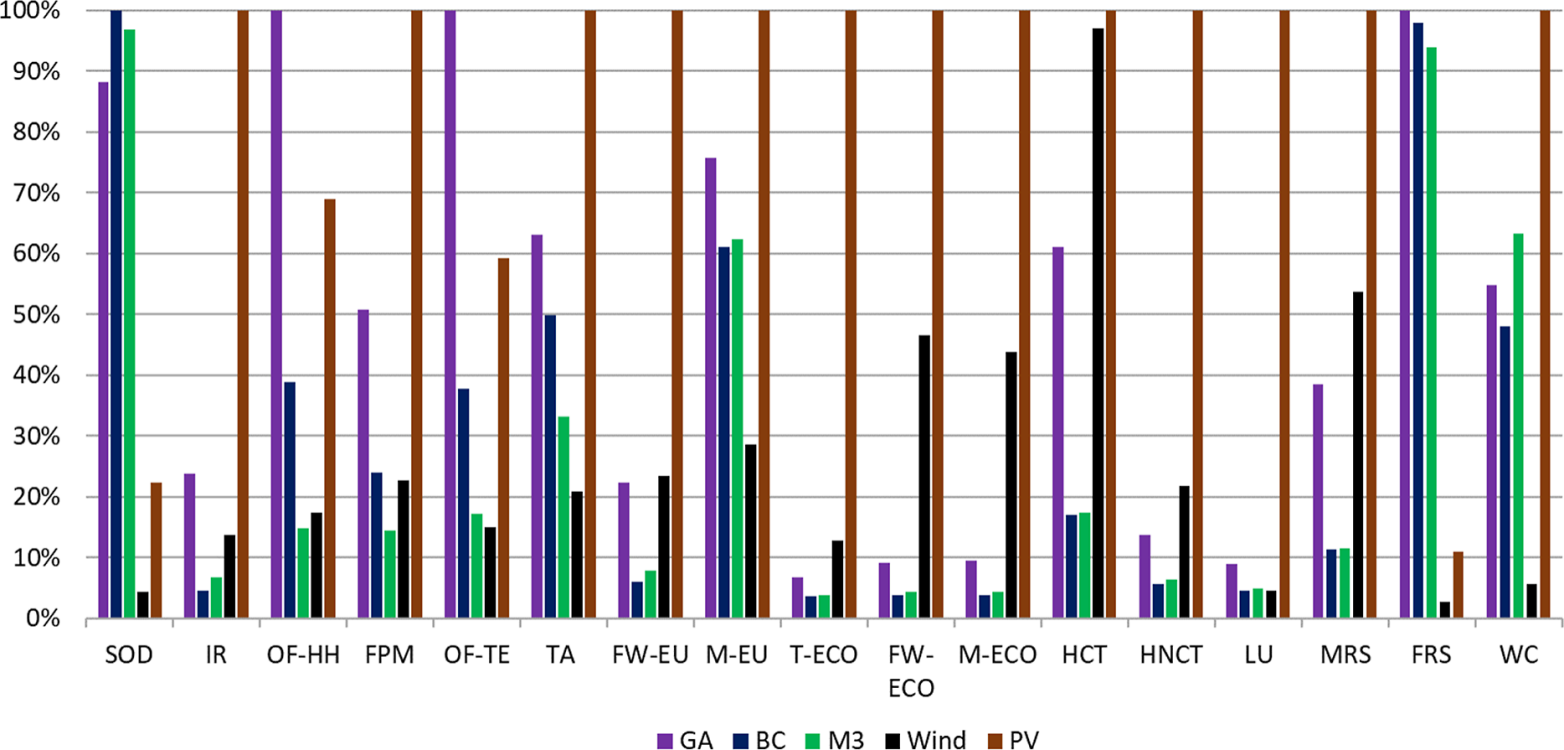
Midpoint environmental impact indicators for CCGT with CCS compared to wind and photovoltaic generation. NG supply scenarios: global average supply (GA), BC average production in 2020 (BC), and BC Montney production with electric drive compressors and 2030 fugitive methane emission reduction target achieved (M3). Results for wind and photovoltaic shown for BC. Results in each environmental impact category normalized to the maximum case in that category. Environmental impact categories: stratospheric ozone depletion (SOD), ionizing radiation (IR), ozone formation–human health (OF-HH), fine particulate matter, ozone formation–terrestrial ecosystems (OF-TEs), terrestrial acidification (TA), freshwater eutrophication (FW-EU), marine eutrophication (M-EU), terrestrial ecotoxicity (T-ECO), freshwater ecotoxicity (FW-ECO), marine ecotoxicity (M-ECO), human carcinogenic toxicity (HCT), human noncarcinogenic toxicity (HNCT), LU, mineral resource scarcity (MRS), fossil resource scarcity (FRS), and water consumption (WC).

CCGT with CCS has considerably higher fossil resource depletion and stratospheric ozone depletion (primarily CCGT nitrous oxide emissions) than renewable energy for all NG supply scenarios (Figure 4). Ionizing radiation, freshwater eutrophication, terrestrial ecotoxicity, freshwater ecotoxicity, marine ecotoxicity, human carcinogenic toxicity, human noncarcinogenic toxicity, and mineral resource scarcity impacts for CCGT with CCS in the BC NG supply scenarios are lower than renewable power generation because of lower material requirements. Land use is much higher for open-ground photovoltaic electricity generation than either wind power or CCGT with CCS. The effect of using engines meeting current NOx emission regulations and electrifying compressor drives in the BC Montney NG scenarios is apparent in the reduced levels of ozone formation and terrestrial acidification impacts. Marine eutrophication in the CCGT scenarios is dominated by supply of amine for absorption of CO_2_ from the exhaust gas, while water consumption is primarily related to CCGT operation (cooling tower). There is a very low variance in impacts between CO_2_ capture scenarios for the other environmental impact categories.

### Effect of Duty Cycles

**Figure 5:**
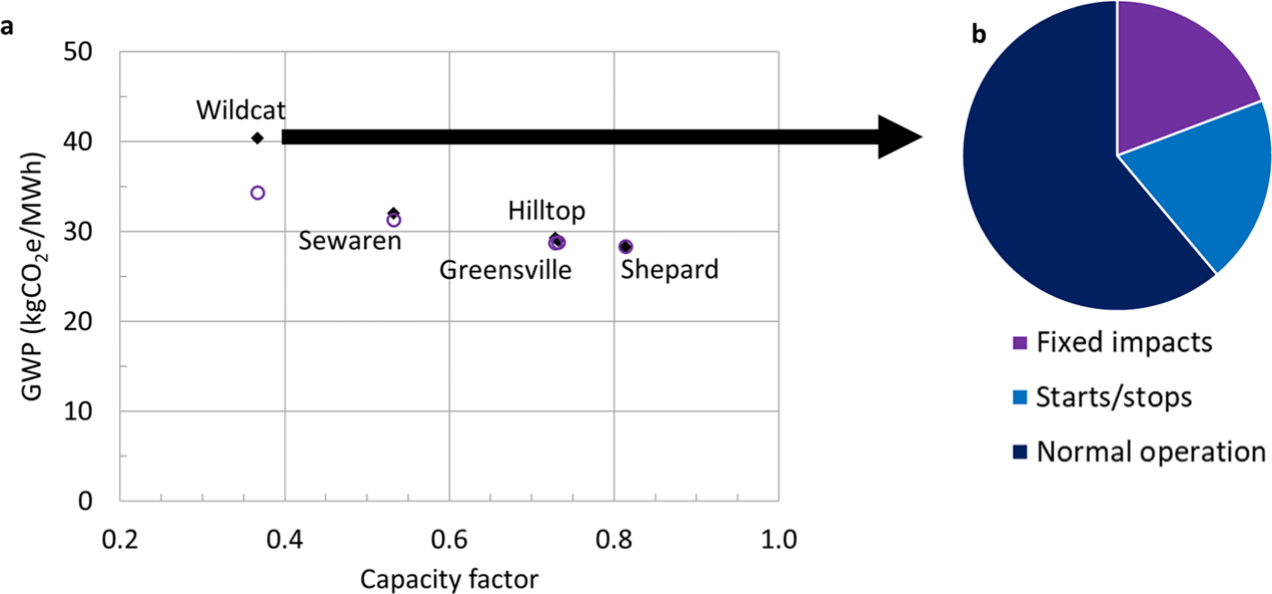
Impact of capacity factor on life cycle GW intensity for CCGT with CCS. (a) Life cycle GW intensity per MWh electricity for CCGT with 97% CO_2_ capture using historical duty cycle data for five existing CCGT facilities without solvent storage (solid black diamonds) and with solvent storage to mitigate startup emissions (hollow purple circles). BC Montney NG supply with electric drive compression and reduced fugitive methane emissions. (b) Contribution of normal operation, fixed infrastructure, and startup/shutdown to life cycle GHG emissions for the Wildcat duty cycle scenario without solvent storage.

Duty cycles from the five existing CCGT facilities considered in this study result in GW intensities 2–46% higher than the baseload assumption without mitigating startup emissions (Figure 5). CO_2_ emissions during startup can be reduced by incorporating interim solvent storage in the process design; however, carbon footprint remains negatively correlated with capacity factor due to the increased relative contribution of fixed infrastructure and the contribution of non-CO_2_ GHGs emitted during startups (primarily uncombusted methane).

The effect of the duty cycle on other environmental impact categories is mixed (Supporting Information Figure 23). Stratospheric ozone depletion, marine eutrophication, fossil resource scarcity, and water consumption impacts are correlated with variable operating inputs, so the corresponding intensities are not materially affected by duty cycle. The other environmental impact categories are more significantly affected by duty cycle depending on the relative contribution of emission sources related to fixed infrastructure.

**Figure 6:**
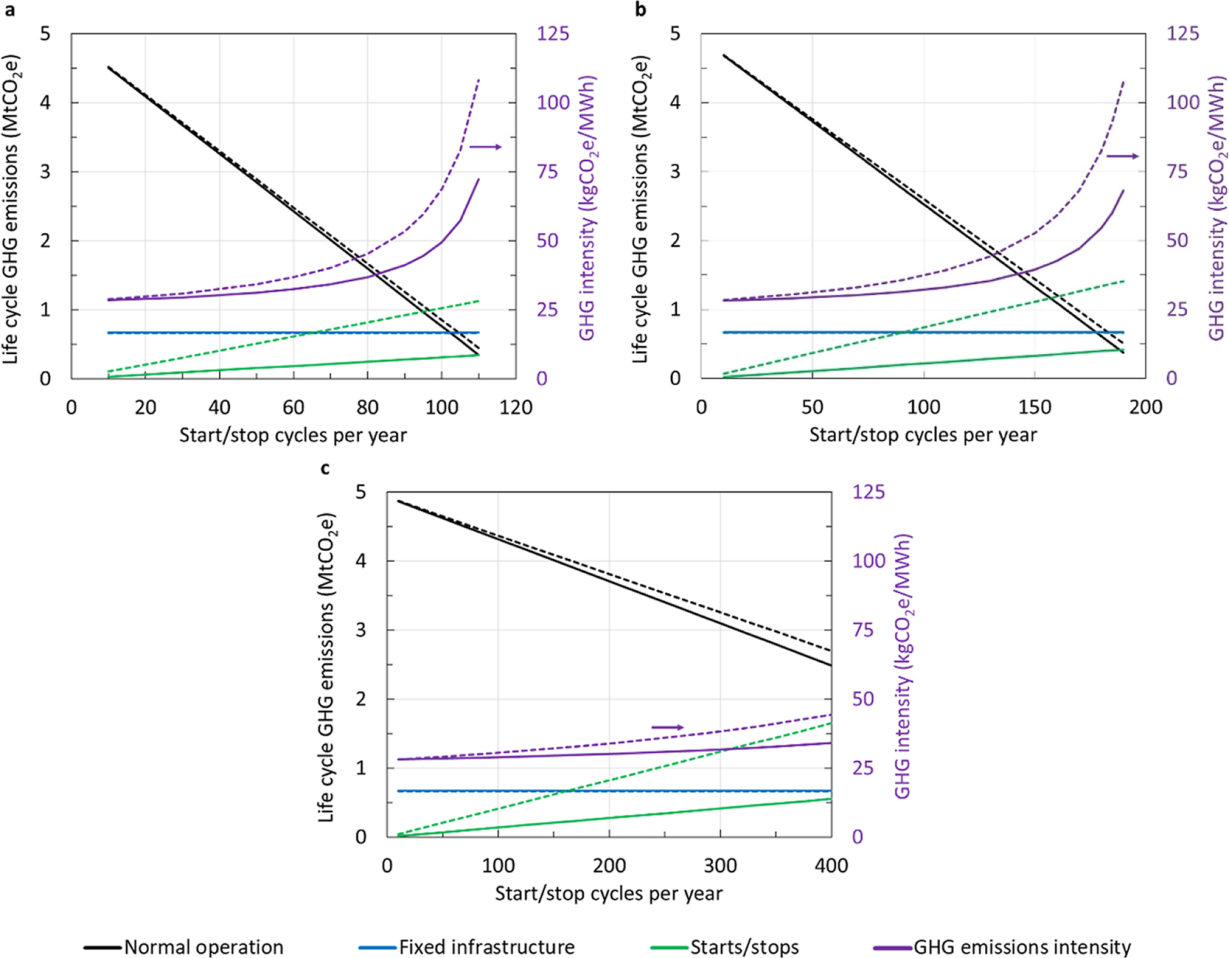
Impact of start/stop cycles on life cycle GHG emissions. Effect of start/stop frequency on the contribution of normal operation, fixed infrastructure, and start/stop cycles to life cycle GHG emissions for CCGT with CCS (LHS) and overall GHG emission intensity (RHS) of electricity produced. Baseline case (dashed) compared to case with interim solvent storage to mitigate startup CO_2_ emissions (solid). CCGT operating at 95% rated output during normal operation with 97% CO_2_ capture. BC Montney NG supply with electric drive compression and reduced fugitive methane emissions. (a) Cold starts, (b) warm starts, and (c) hot starts. Duration of shutdown preceding each hot/warm/cold start is assumed to be 8/36/64 h.

Life cycle GHG emissions associated with start/stop cycles increase linearly with shutdown frequency, while normal operating emissions decrease linearly (Figure 6). Cold starts have a greater effect on GW intensity than warm/hot starts because more emissions are produced and the preceding shutdown is longer which reduces the amount of time the CCGT is operating normally. Interim solvent storage effectively mitigates the CO_2_ emissions associated with startups; however, methane and nitrous oxide emissions remain (c. 15% of GW in cold start without solvent storage). GW intensity increases exponentially with shutdown frequency as the total electricity generated over the life of the CCGT decreases toward zero.

**Figure 7:**
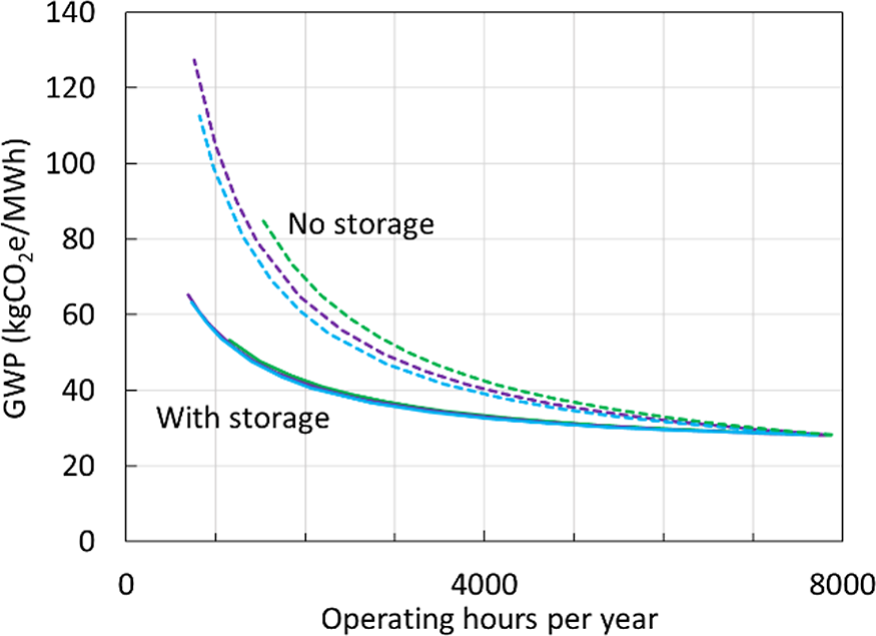
Effect of annual operating hours on life cycle GW intensity. Life cycle GW intensity per MWh electricity produced from CCGT with CCS versus nominal operating hours per year for three different distributions of hot/warm/cold starts: 40/40/20% (blue), 60/30/10% (purple), and 80/15/5% (green). Baseline case (dashed) compared to case with interim solvent storage to mitigate startup CO_2_ emissions (solid). CCGT operating at 95% rated output during normal operation with 97% CO_2_ capture rate. BC Montney NG supply with electric drive compression and reduced fugitive methane emissions.

While there is considerable variance in the impact of hot/warm/cold starts on GHG emissions, there is low variance in the negative correlations between the life cycle GW intensity and nominal operating hours per year over a wide range of startup frequency distributions (Figure 7). Interim solvent storage substantially reduces GW intensity for scenarios with low nominal operating hours (more frequent start/stop cycles). The Supporting Information includes a surrogate model which can be used to estimate life cycle GHG emissions for user-input duty cycle parameters, the CO_2_ capture rate, and NG supply chain emissions.

Other environmental impact intensities are similarly negatively correlated with nominal operating hours per year (Supporting Information Note 3). The strength of the negative correlation varies depending on the relative contributions of fixed infrastructure and startup emissions to each environmental impact category. Using interim solvent storage to mitigate initial CO_2_ emissions does not materially affect environmental impact intensities other than GW.

### Comparisons to Prior Studies

Comparing LCA results between studies is complicated because assumptions about supply chains and performance substantially affect the results. Life cycle GW intensity for baseload CCGT with CCS using global average NG supply and 90% CO_2_ capture in this study (140 kgCO_2_e/MWh) is similar to comparable cases published by US NETL^15^ and Volkart et al.^27^ (163 and 129 kgCO_2_e/MWh, respectively). Steady-state results for carbon intensity in Bui et al.^35^ are close to this study for comparable NG supply assumptions and CO_2_ capture rates (e.g., 75 kgCO_2_e/MWh for 4.9 gCO_2_e/MJ_LHV_ and 90% capture in Bui et al. v. 80 kgCO_2_e/MWh for 5.2 gCO_2_e/MJ_LHV_ and 91% fossil-CO_2_ capture in this study). Startup/shutdown results in this study are not directly comparable to those of Bui et al. because their emission intensity calculations assumed cooldown periods of 1–8 h preceding the startup to reach the different startup states compared to 8–64 h in this study (based on typical industrial equipment). In contrast to Bui et al., this study shows that a process designed with sufficient interim solvent storage that is segregated from the normal process flow can maintain life cycle GW intensity less than 100 kgCO_2_e/MWh regardless of the startup type for plausible ranges of annual starts. While Bui et al. found similar trends of exponentially increasing GW intensity with startup frequency, this study highlights that the primary driver of that exponential increase is not the absolute increase in emissions but the reduction in CCGT electrical output as normal operating hours approach zero.

Life cycle GW intensities reported for wind and photovoltaic power generation depend strongly on assumptions related to supply chain emissions and location—e.g., 28–95 kgCO_2_e/MWh for existing utility-scale photovoltaic facilities worldwide,^83^ 9–250 kgCO_2_e/MWh in a review of 30 LCAs of photovoltaic power generation,^84^ and 4–56 kgCO_2_e/MWh for existing global wind farms.^85^ The values used in this study from Ecoinvent background inventories^68^ for wind (13–18 kgCO_2_e/MWh) and photovoltaic electricity generation (59–77 kgCO_2_e/MWh) are within the corresponding ranges of published values.

Comparing other environmental impact categories with those of prior LCAs is further complicated by different methodologies and impact metrics. Nonetheless, Barbera et al.^16^ also identified shifting environmental burdens comparing CCGT with CCS and wind/photovoltaic power generation that are similar to the comparisons in this study using global average NG supply.

### Climate-Neutral Electricity

Regardless of electricity generation technology, residual life cycle GHG emissions must be reduced to zero to stabilize the climate.^10^ Thus, for CCGT with CCS, there will be an economic trade-off between increasing direct CO_2_ capture from the exhaust stream and offsetting with atmospheric CO_2_ removal (CDR). This study considered gross-CO_2_ capture rates up to 98.5%, but higher capture rates are possible. The unit cost of electricity for CCGT with CCS increased 1.2% in the NETL baseline study for a CO_2_ capture rate of 97% compared to 95%,^14^ while the environmental impacts associated with fixed infrastructure calculated in this study increased less than 1%. Achieving 100% fossil-CO_2_ capture for CCGT would reduce the GW intensity to approximately 19 kgCO_2_e/MWh in the lowest-emission NG supply scenario in this study. Given the small marginal increases in cost and fixed environmental impacts associated with achieving 97% CO_2_ capture, higher capture rates are likely to be economical compared to the current cost of high-permanence CDR.^86,87^

There are also opportunities to mitigate the key drivers of residual GHG emissions in the NG supply chain. Fugitive methane emissions and production flaring could be further abated with regulations requiring operators to implement mitigation measures. Emissions associated with electricity supply should decrease over time as the grid decarbonizes. Drilling and completion operations could be electrified, and decarbonization of steel production will reduce indirect emissions associated with material supply. Finally, process heat emissions could be abated with CCS at the NG processing facilities. It is notable that, in addition to decreasing fugitive methane emission intensity, CO_2_ emission intensity for NG production in BC has also decreased considerably −53% lower in 2021 compared to 2010 (Supporting Information Note 1.3.2).

### Policy Implications

There are many considerations in developing policies for electricity supply, and people perceive those considerations through different economic, social, and political frameworks. Performance and life cycle impacts of wind and photovoltaic power generation vary considerably between jurisdictions.^83,85^ Furthermore, incorporating a large proportion of intermittent renewable generation into an electric grid would require substantial long-term energy storage to avoid production curtailment and supply electricity during periods when real-time generation is lower than demand.^10^ Batteries are frequently proposed for energy storage, but environmental impacts associated with production of current battery technology are considerable—e.g., average life cycle GW of 74 kgCO_2_e/MWh of electricity delivered for lithium ion battery production in a review study of LCAs.^88^ All electricity generation and storage technologies will exhibit an exponential negative correlation between life cycle impact intensities and average capacity factor, as found in this study for CCGT, due to the impacts associated with fixed infrastructure. Therefore, when considering the use of CCGT with CCS to provide dispatchable power, it will be important to compare emission intensity and costs with alternative options given the anticipated duty cycle and capacity factor for the specific application.

This study has shown an approach using existing technology and low-emission production practices that would substantially reduce the GW intensity of electricity generated by CCGT with CCS in NG-producing regions to within the range of published estimates of renewable electricity without considering the additional impacts of energy storage. This finding could provide an opportunity to increase support for more aggressive GHG abatement in NG-producing regions. However, regulatory requirements and/or financial incentives would likely be required to realize the potential reductions, and this study provides evidence that could support the development of future regulations/contracts for low-carbon NG/electricity supply. Cross-border adjustment mechanisms may be required to prevent carbon leakage given widespread interjurisdictional trade in NG and electricity. Prior large-scale postcombustion CCS facilities have not attempted to achieve high CO_2_ capture (>90%) and some have experienced construction delays and/or underperformed compared to expected CO_2_ capture (e.g., refs (89 and 90)); it is crucial that regulations/contracts for low-carbon NG production and CCGTs with CCS require operating emissions to be verified as consistent with GHG mitigation goals. Detailed, case-specific analysis should be employed to compare the financial costs and economic benefits of different options for dispatchable, low-carbon electricity generation, because these will vary substantially between jurisdictions. Extension of the results of this study beyond NG-producing regions would require evaluating options for decarbonizing downstream NG transportation infrastructure (e.g., transmission, storage, and liquefaction/regasification).

Considerable attention has been given recently to regulating methane emissions from NG production, but CO_2_ emissions make up more than half of life cycle emissions in the global average NG supply and 71% in the case of average BC production. For NG consumption to be consistent with net-zero ambitions, both methane and CO_2_ emissions in the supply chain must be reduced to near zero.

It is also important that policy instruments developed to regulate GHG emissions from CCGTs with CCS include emissions during startup and shutdowns to ensure that total life cycle emissions are consistent with GHG abatement objectives given uncertainty in future duty cycles. If expected duty cycles during initial operation do not warrant the additional expense of including interim solvent storage (or other mitigation approaches), then provisions should be included in the facility layout and process design to incorporate mitigation if it becomes justifiable as duty cycles and GHG abatement requirements evolve.

## Supporting Information Available

Detailed information on the life cycle inventory, LCA methodology, NG processing model, and supplementary results (PDF).
Surrogate model for estimating life cycle GHG emissions from CCGTs (XLSX)
