# Supplementary material for: Assessing Best Practices in Natural Gas Production and Emerging CO2 Capture Techniques to Minimize the Carbon Footprint of Electricity Generation
Source: Environ Sci Technol. 2024 Nov 16;58(47):20906–17. doi: 10.1021/acs.est.4c02933 (PMC11603766; doi:10.1021/acs.est.4c02933)
Supplement: Supplementary file 1 — es4c02933_si_001.pdf [file es4c02933_si_001.pdf]

Assessing best practices in natural gas production and emerging CO_2 capture techniques to minimise the carbon footprint of electricity generation

Supporting information

Ryan Cownden[1*], Mathieu Lucquiaud[1]

1. Department of Mechanical Engineering, University of Sheffield, S1 3JD, Sheffield, UK

*Correspondence: ryan.cownden@cownden.ca

Summary: 91 pages, 28 figures, 43 tables.

CONTENTS

1 Supplementary Note 1 – LCA assumptions S-10
1.1 GWP characterisation factors S-10
1.2 Land use change (LUC) methodology S-10
1.3 Life cycle inventory summary S-13
1.3.1 Allocation methodology S-13
1.3.2 Analysis period S-14
1.3.3 Environmentally extended input-output (EEIO) factors S-15
1.4 NG extraction inventory S-15
1.4.1 Transport S-19
1.4.2 Fuel consumption S-19
1.4.3 Surface equipment and pipelines S-20
1.4.4 Flaring S-22
1.4.5 Gas composition S-22
1.4.6 EUR estimation S-23
1.4.7 LUC S-25
1.4.8 Abandonment S-26
1.5 NG processing S-26
1.5.1 Fugitive emissions S-26
1.5.2 Production flaring S-28
1.5.3 Energy inputs S-28
1.5.4 Glycol and amine consumption S-30
1.5.5 Production equipment S-30
1.5.6 Water disposal S-31
1.5.7 Pipeline releases/spills S-32
1.5.8 LUC S-32
1.5.9 Abandonment S-32
1.6 Electricity generation S-32
1.6.1 CCGT load profiles S-32
1.6.2 Normal operating inventory S-34
1.6.3 Startup and shutdown inventory S-38
1.6.4 LUC S-46
1.6.5 Capital and operating/maintenance expenses S-46
1.6.6 Abandonment S-48
1.7 CO_2 sequestration S-48
1.7.1 Fugitive emissions S-50
1.7.2 LUC S-50
1.7.3 Abandonment S-50
1.8 Life cycle impact assessment (LCIA) methodology S-50
1.8.1 Data quality analysis S-51
2 Supplementary note 2 – NG Processing model Details S-53
2.1 Process description S-53
2.2 Process model assumptions S-67

2.3 Process model results S-70
2.4 NG processing plant cost model S-72
3 Supplementary note 3 – Additional results S-74
3.1 NG supply chain impacts S-74
3.2 Baseload CCGT scenarios S-74
3.3 Sensitivity analysis S-82
4 References S-85

LIST OF FIGURES

Supplementary Figure 1. Historical trends of key metrics for BC NG production emissions and industry activity. Based on data reported by the BC Energy Regulator[13] and BC Government[19,20]. **(a)** Annual marketable NG production in BC. **(b)** Annual energy allocation of NG production in BC as a proportion of total energy content (LHV basis) of all marketable NG and liquid hydrocarbon products. **(c)** Annual contribution of stationary combustion, fugitive methane, process CO_2 venting, and flaring to GHG emission intensity of NG production in BC based on energy allocation, 100-year GWP CO_2-equivalence of 29.8 for methane, and reported GHG emissions. **(d)** Number of oil and NG wells drilled in BC per year. S-14

Supplementary Figure 2. Example of decline curve analysis used in this study to estimate EUR. NG production data (purple dots) for BC Montney well drilled in 2012 with stretched exponential decline curves fit through first 28 months of production data (green line) and all available production data (blue line). Data from ref.[13]. S-25

Supplementary Figure 3. Load profiles for the facilities considered in this study. Data shown is the annual average for 2020-2022, except Hilltop is for 2022 only. Based on data from refs.[58,59]. S-34

Supplementary Figure 4. CO_2 capture process operating with interim solvent storage to mitigate startup emissions. Stored lean solvent is pumped through the contactor to separate rich solvent storage until regenerator reaches operating temperature. S-39

Supplementary Figure 5. CO_2 capture process operating in mode to regenerate stored rich solvent. Excess regenerated solvent refills the lean amine storage tank. S-39

Supplementary Figure 6. Overall process model schematic. S-56

Supplementary Figure 7. Inlet separator model schematic. S-57

Supplementary Figure 8. NG compressor model schematic. S-57

Supplementary Figure 9. Amine plant process model schematic. S-58

Supplementary Figure 10. Dehydration plant process model schematic. S-59

Supplementary Figure 11. Refrigeration plant process model schematic. S-60

Supplementary Figure 12. Sales compressor process model schematic. S-61

Supplementary Figure 13. Condensate stabilisation plant model schematic. S-62

Supplementary Figure 14. Recycle compressor model schematic. S-63

Supplementary Figure 15. Acid gas compressor model schematic (first four stages). S-63

Supplementary Figure 16. Acid gas dehydration plant schematic. S-64

Supplementary Figure 17. Acid gas compressor model schematic (final two stages). S-65

Supplementary Figure 18. Water processing model schematic. S-65

Supplementary Figure 19. Vapour recovery unit model schematic. S-66

Supplementary Figure 20. Life cycle environmental impacts of NG production practices. Comparison of life cycle environmental impact intensities for global average NG supply (GA), UK average NG supply (UK), BC average upstream production (BC), and the three low-emission cases developed in this study for BC Montney production with NG drive compressors (M1), electric drive compressors (M2), and electric drive compressors with reduced fugitive emissions (M3). Environmental impact categories: stratospheric ozone depletion (SOD), ionizing radiation (IR), ozone formation – human health (OF-HH), fine particulate matter (FPM), ozone formation

– terrestrial ecosystems (OF-TE), terrestrial acidification (TA), freshwater eutrophication (FW-EU), marine eutrophication (M-EU), terrestrial ecotoxicity (T-ECO), freshwater ecotoxicity (FW-ECO), marine ecotoxicity (M-ECO), human carcinogenic toxicity (HCT), human non-carcinogenic toxicity (HNCT), land use (LU), mineral resource scarcity (MRS), fossil resource scarcity (FRS), and water consumption (WC). Values shown as a percentage of impact intensity for global average NG supply per unit energy on LHV basis. S-74

Supplementary Figure 21. Midpoint environmental impact indicators per MWh electricity produced for CCGT with CCS and six NG supply chain scenarios compared to wind and photovoltaic generation. NG supply scenarios: global average supply (Ecoinvent), UK average supply (Ecoinvent), BC average production (2020), BC Montney production with NG drive compressors, BC Montney production with electric drive compressors, and BC Montney production with electric drive compressors and 2030 fugitive methane emission reduction target achieved. Results for wind and photovoltaic shown for BC (diagonal hatch) and western USA (solid). (a) Global warming potential. (b) Stratospheric ozone depletion. (c) Ionizing radiation. (d) Ozone formation – human health. (e) Fine particulate matter. (f) Ozone formation – terrestrial ecosystems. (g) Terrestrial acidification. (h) Freshwater eutrophication. (i) Marine eutrophication. (j) Terrestrial ecotoxicity. (k) Freshwater ecotoxicity. S-76

Supplementary Figure 22. Midpoint environmental impact indicators per MWh electricity produced for CCGT with CCS and six NG supply chain scenarios compared to wind and photovoltaic generation. NG supply scenarios: global average supply (Ecoinvent), UK average supply (Ecoinvent), BC average production (2020), BC Montney production with NG drive compressors, BC Montney production with electric drive compressors, and BC Montney production with electric drive compressors and 2030 fugitive methane emission reduction target achieved. Results for wind and photovoltaic shown for BC (diagonal hatch) and western USA (solid). (a) Marine ecotoxicity. (b) Human carcinogenic toxicity. (c) Human non-carcinogenic toxicity. (d) Land use. (e) Mineral resource scarcity. (f) Fossil resource scarcity. (g) Water consumption. S-77

Supplementary Figure 23. Effect of CCGT duty cycle on life cycle environmental impact intensities. Comparison of midpoint life cycle environmental impact intensities for electricity generated from CCGT with CCS operating based on average duty cycles for five existing CCGT facilities in North America. Values shown as a percentage of the impact intensities (per MWh of net electricity generated) for the NETL baseload scenario (capacity factor 0.85) for CCGT with 97% CO_2 capture. BC Montney NG supply with electric drive compression and reduced fugitive methane emissions. Environmental impact categories: stratospheric ozone depletion (SOD), ionizing radiation (IR), ozone formation – human health (OF-HH), fine particulate matter (FPM), ozone formation – terrestrial ecosystems (OF-TE), terrestrial acidification (TA), freshwater eutrophication (FW-EU), marine eutrophication (M-EU), terrestrial ecotoxicity (T-ECO), freshwater ecotoxicity (FW-ECO), marine ecotoxicity (M-ECO), human carcinogenic toxicity (HCT), human non-carcinogenic toxicity (HNCT), land use (LU), mineral resource scarcity (MRS), fossil resource scarcity (FRS), and water consumption (WC). S-78

Supplementary Figure 24. Effect of operating hours on life cycle environmental impact intensities. Environmental impacts per MWh electricity produced from CCGT with CCS versus nominal operating hours per year for three different distributions of hot/warm/cold starts. Baseline case (dashed) compared to case with rich solvent storage to mitigate startup CO_2

emissions (solid). CCGT operating at 95% rated output during normal operation. **(a)** Global warming potential. **(b)** Stratospheric ozone depletion. **(c)** Ionizing radiation. **(d)** Ozone formation – human health. **(e)** Fine particulate matter. **(f)** Ozone formation – terrestrial ecosystems. **(g)** Terrestrial acidification. **(h)** Freshwater eutrophication. **(i)** Marine eutrophication. **(j)** Terrestrial ecotoxicity. **(k)** Freshwater ecotoxicity. **(l)** Marine ecotoxicity..S-79
Supplementary Figure 25. Effect of operating hours on life cycle environmental impact intensities. Environmental impacts per MWh electricity produced from CCGT with CCS versus nominal operating hours per year for three different distributions of hot/warm/cold starts. Baseline case (dashed) compared to case with rich solvent storage to mitigate startup CO_2 emissions (solid). CCGT operating at 95% rated output during normal operation. (a) Human carcinogenic toxicity. (b) Human non-carcinogenic toxicity. (c) Land use. (d) Mineral resource scarcity. (e) Fossil resource scarcity. (f) Water consumption. S-80
Supplementary Figure 26. Impact of start/stop cycles on life cycle GHG emissions (UK NG supply). Effect of start/stop frequency on the contribution of normal operation, fixed infrastructure, and start/stop cycles to life cycle GHG emissions for CCGT with CCS (LHS) and overall GHG emission intensity (RHS) of electricity produced. Baseline case (dashed) compared to case with rich solvent storage to mitigate startup CO_2 emissions (solid). CCGT operating at 95% rated output during normal operation with 97% CO_2 capture rate. UK average NG supply based on Ecoinvent background inventory. **(a)** cold starts, **(b)** warm starts, and **(c)** hot starts. Duration of cooldown preceding each hot/warm/cold start is assumed to be 8/36/64 hours.S-81
Supplementary Figure 27. Effect of annual operating hours on life cycle GHG emission intensity (UK NG supply). Life cycle GHG emission intensity per MWh electricity produced from CCGT with CCS versus nominal operating hours per year for three different distributions of hot/warm/cold starts: 40/40/20% (blue), 60/30/10% (purple), and 80/15/5% (green). Baseline case (dashed) compared to case with rich solvent storage to mitigate startup CO_2 emissions (solid). CCGT operating at 95% rated output during normal operation with 97% CO_2 capture rate. UK average NG supply based on Ecoinvent background inventory. S-82
Supplementary Figure 28. Sensitivity analysis of life cycle GW intensity. Percent change in life cycle GHG emission intensity per MWh electricity produced from CCGT with CCS for the baseload scenario (98.5% CO_2 capture) based on changes to indicated parameters. NG supply scenario is BC Montney production with electric drive compressors and fugitive emissions reduced 75% from 2014. Rationale for sensitivity ranges for each parameter provided in Supplementary Note 1.8.1. ....S-84

LIST OF TABLES

Supplementary Table 1. Emissions to air from conversion of forest assumed in this study. Excludes harvested wood products. All quantities in t/ha. S-11
Supplementary Table 2. Parameters used in this study for calculating emissions associated with markets for merchantable timber in BC. S-11
Supplementary Table 3. Average emissions for LUC based on the methodology used in this study. S-12
Supplementary Table 4. Key process activity data for BC Montney well drilling and completions determined in this LCA. Data from BC Energy Regulator[13,23,24]. Mean values and 90% confidence interval for the mean for the wells sampled in this analysis. S-16
Supplementary Table 5. Inventory of drilling mud materials assumed in this study. All quantities are per well. Based on data from BC Energy Regulator[13,25]. Mean values and 90% confidence interval for the mean for the wells sampled in this analysis. S-16
Supplementary Table 6. Inventory of completion chemicals assumed in this study. All quantities are per well. Based on data from BC Energy Regulator[13,23,24]. Mean values and 90% confidence interval for the mean for the wells sampled in this analysis. S-17
Supplementary Table 7. Sample of drill mud materials assumed in this study compared to Nie et al.[28]. All quantities are per well. S-18
Supplementary Table 8. Inventory of disposal waste generated during drilling and completions assumed in this study. All quantities are per well. Based on data from BC Energy Regulator[13,23,24]. S-18
Supplementary Table 9. Gas compositions assumed in this LCA. Montney gas composition based on the average of the two reporting areas (Heritage and Northern). BC Average is based on production weighted average of marketable NG in the transmission pipelines. Data from refs.[13,20,24,33]. S-23
Supplementary Table 10. Condensate composition assumed in this LCA. Montney condensate composition based on the average of a sample of typical wells drilled in 2020. Data from ref.[13,24]. S-23
Supplementary Table 11. Breakdown of land use assumption for NG wells. S-26
Supplementary Table 12. Calculated energy allocations for marketable oil and gas products in BC during 2020. Based on reported production volumes[20] and assumed energy densities (LHV basis)[33,48-50]. S-27
Supplementary Table 13. Stationary combustion emission intensities assumed for baseline. Based on BC government reported stationary combustion GHG emissions for oil and NG industry[43] and government of Canada reported air pollutant emissions inventory[51] in 2020. Based on reported production volumes[20] and assumed energy densities[33,48-50]. Estimated drilling and completion (D&C) emissions are subtracted from reported emissions to avoid double counting. S-29
Supplementary Table 14. US EEIO categories used to model environmental impacts for the NG processing facility construction, electrical materials, instrumentation, and facility maintenance. Plant construction costs in million USD and annual maintenance costs in million USD per year with Q1 2018 base year. S-31

Supplementary Table 15. Summary of operating parameters for the existing CCGT facilities considered in this study. Data shown is the annual average for 2020-2022, except Hilltop is for 2022 only. Shutdowns and restarts are average number per year. Based on data from refs.[58,59]. S-33
Supplementary Table 16. CCGT NG consumption, electricity output, and air emissions inventory for normal operation assumed in this study. CCGT output is percent of maximum for each CO_2 capture scenario. Net CO_2 emitted excludes CO_2 that entered the CCGT with the inlet air. Calculated using data from refs.[31,64,69] and part load correlations from Spitz et al.[66]. S-36
Supplementary Table 17. CCGT consumable inventory assumed in this study. Calculated using data from the NETL baseline study[64]. 2018 base year for consumable costs. S-37
Supplementary Table 18. Description of hot startup sequence assumed in this study. S-40
Supplementary Table 19. Description of warm startup sequence assumed in this study. S-40
Supplementary Table 20. Description of cold startup sequence assumed in this study. S-41
Supplementary Table 21. CCGT life cycle inventory for hot startups with solvent storage assumed in this study. Net CO_2 emitted excludes CO_2 that entered the CCGT with the inlet air. Values shown are per startup. S-42
Supplementary Table 22. CCGT life cycle inventory for warm startups with solvent storage assumed in this study. Net CO_2 emitted excludes CO_2 that entered the CCGT with the inlet air. Values shown are per startup. S-43
Supplementary Table 23. CCGT life cycle inventory for cold startups with solvent storage assumed in this study. Net CO_2 emitted excludes CO_2 that entered the CCGT with the inlet air. Values shown are per startup. S-43
Supplementary Table 24. CCGT life cycle inventory for hot startups without solvent storage assumed in this study. Net CO_2 emitted excludes CO_2 that entered the CCGT with the inlet air. Values shown are per startup. S-44
Supplementary Table 25. CCGT life cycle inventory for warm startups without solvent storage assumed in this study. Net CO_2 emitted excludes CO_2 that entered the CCGT with the inlet air. Values shown are per startup. S-44
Supplementary Table 26. CCGT life cycle inventory for cold startups without solvent storage assumed in this study. Net CO_2 emitted excludes CO_2 that entered the CCGT with the inlet air. Values shown are per startup. S-45
Supplementary Table 27. Description of shutdown sequence assumed in this study. S-46
Supplementary Table 28. CCGT life cycle inventory for shutdowns assumed in this study. Net CO_2 emitted excludes CO_2 that entered the CCGT with the inlet air. Values shown are per shutdown. S-46
Supplementary Table 29. Construction material inventory for CO_2 capture, compression, and dehydration equipment for 758 MW CCGT. Data from the Panda Sherman front-end engineering design study[70] with 2021 base year (USD) for EEIO categories. S-47
Supplementary Table 30. Construction cost estimates for CO_2 capture, compression, and dehydration equipment assumed in this study. Based on data from the NETL baseline study[64] with 2018 base year (million USD). S-48
Supplementary Table 31. Maintenance labour cost estimates for CCGT with CCS assumed in this study. All amounts in million USD/year with 2018 base year. Based on data from the NETL baseline study[64]. S-48

Supplementary Table 32. Summary of sensitivity analyses conducted in this study. S-52
Supplementary Table 33. Amine sweetening design parameters used in this study. S-68
Supplementary Table 34. Dehydration plant design parameters used in this study..............S-69
Supplementary Table 35. Refrigeration plant design parameters used in this study.............S-69
Supplementary Table 36. Condensate stabiliser design parameters used in this study. S-69
Supplementary Table 37. Acid gas dehydration plant design parameters used in this study.S-70
Supplementary Table 38. Shaft power calculated in the NG processing plant model. All values are kW for the design cases with NG engine driven inlet compressors and electric motor driven inlet compressors. S-71
Supplementary Table 39. Process heat requirements calculated in the NG processing plant model. All values are MJ/h for the design cases with NG engine driven inlet compressors and electric motor driven inlet compressors. S-71
Supplementary Table 40. Electricity and NG consumption estimates from the NG processing plant model. Values are provided for the design cases with NG engine driven inlet compressors and electric motor driven inlet compressors. S-71
Supplementary Table 41. Overall flows from the NG processing plant model. Values are provided in energy content (GJ/h, LHV basis) for the design cases with NG engine driven inlet compressors and electric motor driven inlet compressors. S-72
Supplementary Table 42. Required well effluent, fuel, and electricity inputs to NG processing plant. Values expressed per unit of energy produced by the NG gas processing plant LHV basis (GJ_{out}) for the design cases with NG engine driven inlet compressors and electric motor driven inlet compressors. Total energy production includes liquid condensate co-product allocation.S-72
Supplementary Table 43. Capital and maintenance cost estimates for NG processing plant assumed in this study for each design case. Amounts in US$ for North America with base year of 2018..........S-73

1 SUPPLEMENTARY NOTE 1 – LCA ASSUMPTIONS

1.1 GWP characterisation factors

The 100-year GWP characterisation factors for GHGs used in this analysis were based on the IPCC Sixth Assessment Report (AR6)[1]: 29.8 for methane and 273 for nitrous oxide. An updated characterisation factor for carbon monoxide was not included in AR6, so a value of 1.69 was assumed based on AR5[2].

1.2 Land use change (LUC) methodology

We assumed original landcover is 88% forest and 12% agricultural based on satellite imagery of the Montney field location[3,4] and BC landcover classification[5].

For the forest area, we assumed that merchantable timber is salvaged for use in harvested wood products and non-merchantable biomass is burned. We used estimated average carbon stock for the Canadian Boreal Plains region of 225 tC/ha distributed as 25.5% live biomass, 24% litter, 10.5% deadwood, and 40% soil[6]. An assumed carbon fraction of 0.5 tC/t_{dry} [7] resulted in total biomass of 270 t_{dry}/ha. We used IPCC methodology and factors for boreal forest[8] and roundwood density of 0.386 odt/m^3 [7] to estimate merchantable timber of 60 t_{dry}/ha. We followed IPCC methodology and factors[8] to estimate emissions from combustion of the residual 210 t_{dry}/ha of non-merchantable biomass (**Supplementary Table 1**). Uncombusted carbon in the biomass was assumed to convert to CO_2 through decomposition of the residual slash in the first year following disturbance. We assumed 25% soil carbon loss in alignment with Yeh et al.[9] and slightly higher than IPCC guidelines (20%)[8]. We did not consider foregone sequestration during industrial use because the assumed carbon stock when cleared is based on relatively mature boreal forest which has an annual rate of carbon accumulation that is insignificant compared to other carbon flows in this analysis[6].

Supplementary Table 1. Emissions to air from conversion of forest assumed in this study. Excludes harvested wood products. All quantities in t/ha.

	Biomass combustion	Biomass decomposition	Soil carbon loss
CO_2	194	168	83
Carbon monoxide	13.2		
Methane	0.582		
Nitrous oxide	0.0322		
Nitrogen oxides (NO_x)	0.371		

We assumed merchantable timber is converted to harvested wood products in proportion to the BC forest industry fiber flow distribution in 2020[10] with half-lives based on IPCC guidelines[8]. Carbon in pellet products was assumed to completely convert to CO_2 in the first year. We calculated emissions from decay of wood products over a 100-year period from the date of disturbance. Overall, approximately 94% of carbon in the wood products is converted to CO_2 over this period resulting in emissions of 103 tCO_2/ha which were allocated back to the land conversion to maintain carbon balance during regrowth. Impacts associated with log transport and processing of merchantable timber were allocated to the wood products and were not explicitly quantified in this study. We did not include any indirect LUC credit for reduced logging activity due to log supply to the forest products industry.

Supplementary Table 2. Parameters used in this study for calculating emissions associated with markets for merchantable timber in BC.

	Allocation	Half-life (years)
Lumber	41.3%	35
Oriented strand board	7.1%	25
Pellets	10.1%	N/A
Pulp	41.5%	2

We assumed soil carbon on agricultural land is 70% of forest soil carbon[8] and that 25% is lost due to conversion to industrial use resulting in 58 tCO_2/ha of agricultural land disturbance. We ignored carbon stored in biomass on agricultural land in alignment with IPCC methodology[8]. We included indirect land use change associated with conversion of agricultural land by assuming that an equal area of Canadian Boreal Plains forest is temporarily converted to agricultural land

and then returned to forest after the industrial development is abandoned. We did not include the impact on climate change from modifying surface albedo through LUC, but note that the warming effect of trees in the boreal region of Canada has been found to exceed the cooling effect of sequestered carbon[11,12].

For the CCGT facility, NG processing plant, and CO_2 disposal wells/pipelines, we assumed site construction takes 2 years, operating life is 30 years, and decommissioning takes 1 year (duration of disturbance is 33 years). Although we limit the life of each individual well to 20 years for calculating production volume, we assume the same duration of disturbance for NG production wellsites, pipelines, and roads because drilling and completion activities on each wellsite are typically staged over several years[13] and the model assumes that pipeline and road infrastructure is shared between well pads.

Following decommissioning, we assumed disturbed land is reclaimed to its original use. Carbon sequestered following reversion to original use was calculated up to 100 years from the initial disturbance. For forest land, we assumed net ecological productivity during regrowth increases linearly from 0 to 2.1 tC/ha-year over 20 years, continues at 2.1 tC/ha-year for 30 years, and then decreases linearly to 0 tC/ha-year over 70 years based on typical profiles and measurements of Canadian boreal forests[6,8,11,14-16]. For agricultural land, we assumed soil carbon increases linearly to its pre-disturbance level over 20 years[8].

This methodology results in net emissions over a 100-year timeframe of 173 tCO_2e/ha (**Supplementary Table 3**). Our results are slightly higher than the mid range result of 157 tCO_2e/ha calculated in Yeh et al.[9] for conventional oil production on boreal land in Alberta, Canada using slightly different methodology.

Supplementary Table 3. Average emissions for LUC based on the methodology used in this study.

	Life cycle emissions (t/ha)
CO_2	125
Carbon monoxide	13.2
Methane	0.582
Nitrous oxide	0.0322
Nitrogen oxides (NO_x)	0.371

1.3 Life cycle inventory summary

The description of the life cycle inventory is segregated into the following sections:

- NG extraction: materials and fuel consumed during drilling and completion of NG production wells. Also includes wellsite production equipment, pipelines for NG gathering and fuel gas/water distribution, access roads, disposal of waste generated, LUC, flaring during initial flow back, and abandonment;
- NG processing: installation and operation of equipment to produce NG to supply CCGT. Includes fuel combustion, operational flaring, fugitive emissions, materials for equipment, maintenance, LUC, chemical consumption, and abandonment;
- Electricity generation: installation and operation of CCGT and CO_2 capture/compression. Includes direct emissions, LUC, chemical consumption, capital equipment, maintenance, and abandonment; and
- CO_2 sequestration: materials and fuel consumed during drilling and completion of sequestration wells. Also includes CO_2 pipelines, access roads, disposal of waste generated, LUC, fugitive emissions, and abandonment.

1.3.1 Allocation methodology

Where necessary, allocation of inventory between NG and co-produced liquid hydrocarbons has been based on energy content (LHV). Energy basis was deemed more appropriate than economic basis for this forward-looking analysis due to uncertain future prices; it was also believed more reasonable to allocate emissions based on the physical contribution of each commodity towards meeting the energy demands of society. Since this study is focused on assessing impacts associated with NG production, energy allocation is a more conservative approach than economic allocation, resulting in higher impacts, because liquid hydrocarbon commodity prices in western Canada are significantly higher than NG per unit of energy[17].

1.3.2 Analysis period

2020 was selected as the period for analysis in this study as a balance between using recent inventory data to reflect current industry practices and ensuring that sufficient production data is available from each well to develop an accurate forecast of estimated ultimate recovery (EUR). While some studies (e.g., ref. [18]) have reported significant impacts from the COVID-19 pandemic on GHG emissions from oil and NG production in other regions, we found no evidence that overall annual emissions or activity for NG production in BC was significantly affected outside of historical ranges and trends (**Supplementary Figure 1**).

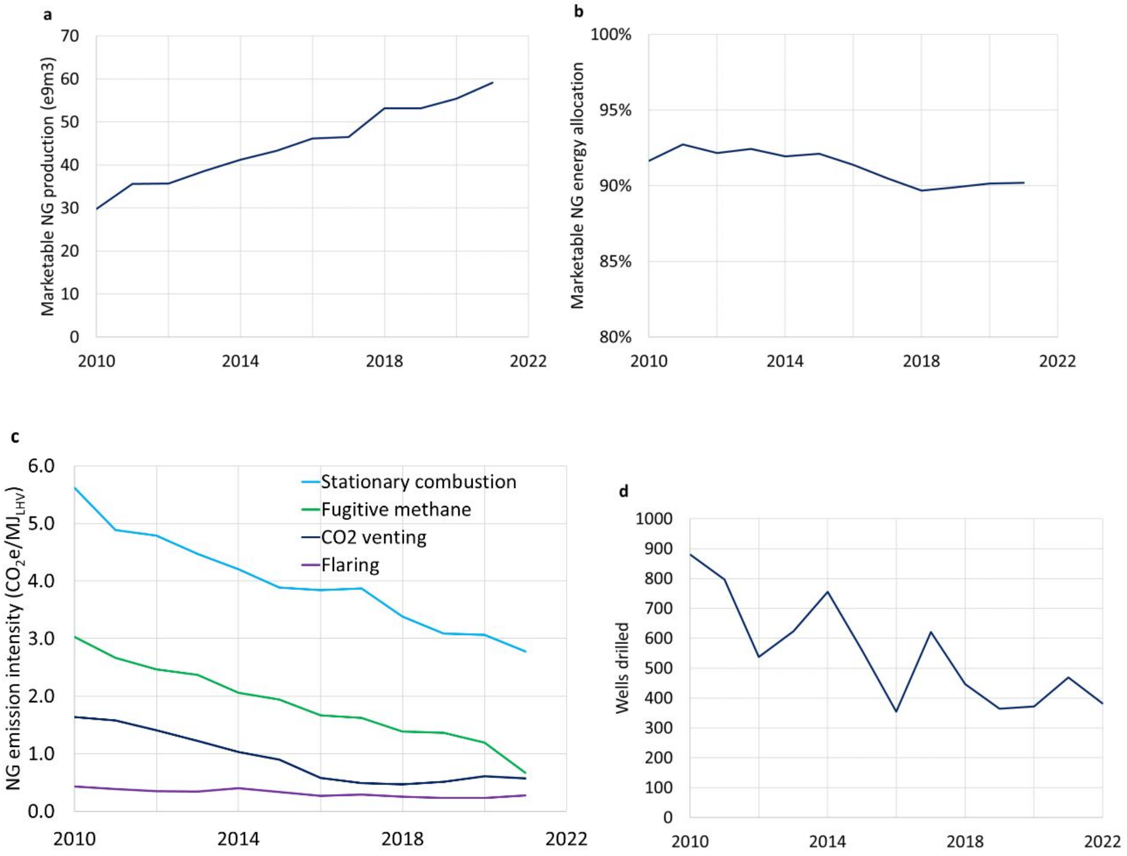

Supplementary Figure 1. Historical trends of key metrics for BC NG production emissions and industry activity. Based on data reported by the BC Energy Regulator[13] and BC Government[19,20]. **(a)** Annual marketable NG production in BC. **(b)** Annual energy allocation of NG production in BC as a proportion of total energy content (LHV basis) of all marketable NG and liquid hydrocarbon products. **(c)** Annual contribution of stationary combustion, fugitive methane, process CO_2 venting, and flaring to GHG emission intensity of NG production in BC based on energy allocation, 100-year GWP CO_2-equivalence of 29.8 for methane, and reported GHG emissions. **(d)** Number of oil and NG wells drilled in BC per year.

1.3.3 Environmentally extended input-output (EEIO) factors

US Environmental Protection Agency EEIO impact factors were used in this analysis because of high disaggregation compared to other datasets[21]. Relevant data for Canada are expected to be similar due to the integrated North American economy[22].

1.4 NG extraction inventory

Data were collected for 61 wells (18% sample) operated by the five companies that drilled the most BC Montney wells in 2020 (collectively 86% of wells drilled[13]). The sample was distributed across different well pads owned by the five operators in proportion to their relative share of the overall number of wells (i.e., 20% of wells drilled by each company) to ensure a geographically and operationally representative sample. Due to differences in operating practices and reporting standards, some data were not available for the entire sample.

In addition to the inventory of wellbore materials, fuel consumed during drilling/completions, invert losses, and NG flare volume (**Supplementary Table 4**), inventories were also compiled for drilling fluid additives (

Supplementary Table 5) and completion fluid additives (**Supplementary Table 6**) from reports in the BC Energy Regulator public database[13,23,24]. The drilling mud and completion chemical inventories are based on averages of data for multiple wells across multiple operators and are not intended to represent a specific well. Materials with an average of less than 0.2 t (solids) or m^3 (liquids) were ignored in the calculations. This cut-off criteria resulted in inclusion of c. 99% of drilling mud materials and 99.999% of completions materials. NG for drilling and completions operations was assumed to be supplied from local Montney NG production. EURs for NG and condensate were used to calculate the total energy content of the combined effluent stream for each well based on typical Montney compositions (Supplementary note 1.4.5). Background inventories associated with supply of these materials was modelled by selecting appropriate library processes from the Ecoinvent database.

Supplementary Table 4. Key process activity data for BC Montney well drilling and completions determined in this LCA. Data from BC Energy Regulator[13,23,24]. Mean values and 90% confidence interval for the mean for the wells sampled in this analysis.

Process activity data	Mean	90% confidence interval	Units
Steel in wellbore casing	140	132-148	t/well
Steel in wellbore tubing	15.2	14.7-15.7	t/well
Cement in wellbore	132	125-139	t/well
Diesel consumed during drilling	47.3	42.2-52.3	m^3/well
NG consumed during drilling	8.9	0-19.3	E3m^3/well
Fluid loss during invert drilling	30.7	24.9-36.4	m^3/km
Length of wellbore drilled with invert	1645	1367-1923	m/well
Diesel consumed during completions	80.1	63.9-96.2	m^3/well
NG consumed during completions	48.4	29.9-66.9	E3m^3/well
NG flared during completions	20.3	9.1-31.4	E3m^3/well
NG EUR	146	123-169	E6m^3/well
Condensate EUR	4820	2140-7490	m^3/well
Combined well effluent EUR (LHV basis)	6.30	5.33-7.27	PJ/well

Supplementary Table 5. Inventory of drilling mud materials assumed in this study. All quantities are per well. Based on data from BC Energy Regulator[13,23,24]. Mean values and 90% confidence interval for the mean for the wells sampled in this analysis.

Material	Mean	90% confidence interval	Units
Barium sulphate	55.6	26.4 – 84.8	T
Bentonite clay	2.8	1.3 – 4.4	T
Calcium ammonium nitrate	1.3	0 – 3.6	T
Calcium carbonate	0.3	0 – 0.9	T
Calcium chloride	17.4	9.9 – 24.8	T
Calcium nitrate	9.0	4.9 – 13.1	T
Caustic soda (NaOH)	0.4	0.2 – 0.5	T
Corrosion inhibitor (50% methanol, 50% ethanol products)	0.6	0.3 – 0.9	m^3
Emulsifier (60% middle distillates, 40% tall oil)	0.7	0.3 – 1.1	m^3
Fluid loss additive (80% asphalt, 20% aluminum silicate)	0.5	0.1 – 0.8	T
Graphite	0.3	0 – 0.5	T
Lime (CaO)	4.3	3.4 – 5.3	T
Lubricant (modified fatty acids)	0.5	0.1 – 0.9	m^3
Oxygen scavenger (diethyl hydroxylamine)	0.3	0.1 – 0.5	m^3
Oxygen scavenger (sodium sulphite)	0.9	0.3 – 1.6	T
Polymer beads	0.6	0.3 – 0.9	T
Sawdust	1.6	0.9 – 2.4	T
Sodium bicarbonate	0.5	0 – 1.3	T
Surfactant (middle distillates)	0.9	0.3 – 1.6	m^3
Walnut shells	1.1	0.3 – 1.8	T
Wetting agent (ethylene glycol)	0.9	0 – 2.2	m^3

Supplementary Table 6. Inventory of completion chemicals assumed in this study. All quantities are per well. Based on data from BC Energy Regulator[13,23,24]. Mean values and 90% confidence interval for the mean for the wells sampled in this analysis.

Material	Mean	90% confidence interval	Units
Water	13 990	12 570 – 15 410	m^3
Sand (placed)	3090	2730 – 3440	t
Hydrochloric acid (30% wt.)	13.5	8.08 – 18.8	m^3
Methanol	2.7	1.5 – 3.9	t
Biocide	0.85	0.7 – 1.0	m^3
Friction reducer (liquid)	12.6	7.8 – 17.5	m^3
Friction reducer (solid)	2.8	1.3 – 4.2	t
Surfactant	4.4	3.1 – 5.7	m^3
Scale inhibitor	0.80	0.4 – 1.2	m^3
Nitrogen	4.3	2.1 – 6.5	$E3m^3$

Fuel consumption during drilling and completions in this study is 10% and 12% lower respectively on an energy basis (LHV) than an estimate for the Marcellus shale in the US[25] which was based on substantially higher water injection volume during completions (39 000 m^3/well). Fuel consumption assumed in studies of Montney wells in Alberta, Canada[26,27] is significantly higher than this study due to the larger/longer drill and significantly larger quantity of fluid pumped during hydraulic fracturing compared to the average in this study.

Wellbore steel and concrete assumed in this study is significantly lower than Nie et al.[27] for Montney wells in Alberta, Canada in 2016 (291 and 381 t respectively) due to the larger assumed diameter hole and more complex wellbore design in Nie et al. than employed in typical Montney wells in BC in 2020. Drill mud materials assumed in this study are similar to the materials presented in Nie et al.[27]; however, some of the quantities used in this study are significantly higher (**Supplementary Table 7**). Drill mud materials typically exhibit significant variability depending on the reservoir characteristics. Significantly larger quantities of completions chemicals were assumed in Nie et al.[27] (e.g., 7198 t sand, 18.6 t friction reducer, and 205 m^3 acid) compared to this study reflecting the larger hydraulic fracture fluid volume used by the production company in their study (43 900 m^3/well) compared to the BC Montney average in our

study. Other studies which provide a similar level of detail for drilling and completions life cycle inventories were not identified for comparison.

Supplementary Table 7. Sample of drill mud materials assumed in this study compared to Nie et al.[27]. All quantities are per well.

Material	This study	Nie et al.
Barium sulphate	55.6 t	20 t
Bentonite clay	2.8 t	2.7 t
Calcium chloride	17.4 t	1.8 t
Calcium nitrate	9.0 t	8.2 t
Lime	4.3 t	7.5 t
Sawdust	1.6 t	2.7 t
Invert/Base oil	38.4 m^3	20 m^3

We assumed that drill mud is stored and reused from well to well. Drill cuttings from the wellbore and sand recovered at surface during initial flowback of the well during completions (**Supplementary Table 8**) were assumed to be hauled for processing and disposal at a hazardous waste facility near Fort St. John, BC. Drill cuttings were estimated for each well based on hole diameter and depth, a bulk density of 2.6 t/m^3, and the mass of cuttings increased by 25% to account for residual drill mud moisture and solids[28]. We modelled the emissions/impacts associated with transport and processing/disposal using the Ecoinvent library process "Drilling waste (RoW), market for drilling waste". Prior LCAs which included details regarding drilling and completions waste material handling were not identified for comparison.

Supplementary Table 8. Inventory of disposal waste generated during drilling and completions assumed in this study. All quantities are per well. Based on data from BC Energy Regulator[13,23,24].

Material	Mean	90% confidence interval	Units
Drill cuttings	379	364 – 393	t
Flowback sand	23.4	19.9 – 26.8	t

We assumed that drilling and completions equipment is used on enough wells that the allocation of impacts associated with the manufacturing of the equipment to each individual well is negligible compared to the materials and energy consumed drilling and completing each well.

1.4.1 Transport

Drilling and completions equipment was assumed to be mobilised from Fort St. John, BC to each well pad to work on 5 wells per mobilisation based on the average of Montney formation data for the top five operators by drilling activity in BC in 2020[13]. Drilling equipment transport was based on dedicated heavy haul transport for the rig (e.g., ref.[29]) and ancillary equipment (e.g., office trailers, washrooms, garbage bins, generators, light towers, etc.) with separate return trips for mobilisation and demobilisation – total freight transport of 60 000 tkm per well was assumed. Based on typical completions equipment used for the Montney formation in BC[13], equipment transport was based on 30 heavy haul loads with dedicated trucks making a single round trip per mobilisation/demobilisation (e.g., fluid pumpers, blenders, hydration units, coiled tubing, data van) plus 30 heavy haul loads making two round trips per mobilisation/demobilisation (e.g., rental equipment, offices, tanks) – total freight transport of 90 000 tkm per well was assumed.

Transport for materials consumed in well drilling and completions operations was included in the Ecoinvent library inventories.

1.4.2 Fuel consumption

Air emissions inventory for fuel consumption during drilling and completions of new wells was calculated from the activity data (**Supplementary Table 4**). 80% of diesel consumption during drilling operations was assumed to supply internal combustion engines and 20% to supply boilers based on typical consumption data[13]. All NG consumption during drilling operations was assumed to supply boilers. All diesel and NG consumption during completion operations was conservatively assumed to supply internal combustion engines operating in dual-fuel mode (simultaneous combustion of diesel and NG) based on public completions reports[13]. Ecoinvent library processes were used as the basis for determining pollution from stationary combustion sources to include trace pollutants and were adjusted to align with the conditions of this study as follows:

1. Drilling NG boiler emissions were modelled using “Heat, central or small-scale, natural gas (RoW)| heat production, natural gas, at boiler fan burner low-NOx non-modulating

<100kW | Cut-off, U" modified for fuel supplied from internal BC Montney production, CO_2 emissions increased based on gas composition, and 90% efficiency.

2. Drilling diesel boiler emissions were modelled using "Heat, central or small-scale, other than natural gas (RoW)| heat production, light fuel oil, at boiler 100kW, non-modulating | Cut-off, S" with no modifications and assuming 90% efficiency.
3. Drilling diesel engine emissions were modelled using "Diesel, burned in diesel-electric generating set, 10MW, for oil and gas extraction (GLO)| market for diesel, burned in diesel-electric generating set, 10MW, for oil and gas extraction | Cut-off, S" with no modifications.
4. Completions dual fuel engine emissions were modelled using "Diesel, burned in diesel-electric generating set, 10MW, for oil and gas extraction (GLO)| diesel, burned in diesel-electric generating set, 10MW, for oil and gas extraction | Cut-off, U" modified to reflect 60% of fuel supply from diesel and 40% from NG on LHV basis. The CO_2 emission factor was adjusted to reflect this fuel supply mix. Emission factors for carbon monoxide, methane, nitrogen oxides, and non-methane volatile organic compounds were adjusted to match US EPA emission factors for dual fuel engines[30]. The emission factor for sulphur dioxide was adjusted to match Canadian fuel standards (maximum 0.015% sulphur by weight for diesel). Other pollutants were left unchanged.

1.4.3 Surface equipment and pipelines

We assumed 15 t of steel material per well associated with the wellhead, surface piping, wellsite production equipment, and support piles based on typical wet metering facility layouts employed within the BC Montney field[13,23,31] ("Steel, low-alloyed, hot rolled (GLO)| market for steel, low-alloyed, hot rolled | Cut-off" plus "Drawing of pipe, steel (GLO)| market for drawing of pipe, steel | Cut-off").

We assumed each well pad ultimately has 30 wells and that pads are spaced 2 km apart with three pipelines between each pad based on typical Montney field layouts for the major producers[23,31]: 219 mm diameter x 6.35 mm steel pipe for NG production, 89 mm x 4 mm steel

pipe for fuel gas, and 236 mm composite pipe for water. We used Ecoinvent library processes to provide inventories for the installation of the multiple pipelines in the same ditch:

- "Steel, low-alloyed, hot rolled (GLO)| market for steel, low-alloyed, hot rolled| Cut-off, S" plus "Drawing of pipe, steel (GLO)| market for drawing of pipe, steel | Cut-off, S" for steel pipe (total of 41.6 t per km);
- "Steel, low-alloyed, hot rolled (GLO)| market for steel, low-alloyed, hot rolled" plus "Sheet rolling, steel (GLO)| market for sheet rolling, steel | Cut-off, S" for steel portion of composite pipe (total of 17.6 t per km);
- "Welding, arc, steel (RoW)| welding, arc, steel| Cut-off, S" for field joint welds (total of 53.8 m per km);
- "Pitch (RoW)| market for pitch| Cut-off, S" for steel pipe coating adhesive (total of 174 kg per km);
- "Polyethylene, high density, granulate (GLO)| market for polyethylene, high density, granulate | Cut-off, S" for steel pipe external coating and plastic component of composite pipe (total of 14.4 t per km);
- "Excavation, skid-steer loader (RoW)| excavation, skid-steer loader| Cut-off, S" for topsoil removal (0.3 m deep by 10 m wide) along right-of-way (soil volume was doubled to account for removal and replacement);
- "Excavation, hydraulic digger (RoW)| excavation, hydraulic digger| Cut-off, S" for excavation of trench (1.7 m deep x 2 m wide) along right-of-way (soil volume was doubled to account for removal and replacement);
- "Machine operation, diesel, >= 74.57 kW, low load factor (GLO)| machine operation, diesel, >= 74.57 kW, low load factor| Cut-off, S" for pipe layers (30 h per km); and
- "Transport, passenger car, large size, petrol, EURO 5 (GLO)| market for transport, passenger car, large size, petrol, EURO 5| Cut-off, S" for personnel transport on location (2000 km per km of pipeline).

We assume that an 8 m wide road is constructed on a 15 m wide right-of-way between each well pad. Road construction was modelled using the Ecoinvent library process "Road, company, internal (RoW)| road construction, company, internal| Cut-off, S".

1.4.4 Flaring

Flaring emissions were modelled starting with the Ecoinvent library process "Waste natural gas, sweet (GLO)| treatment of waste natural gas, sweet, burned in production flare". This process was modified for CO_2 emissions to reflect the Montney average wellhead composition (**Supplementary Table 9**) and to add methane, nitrous oxide, sulphur dioxide, and nitrogen oxides. The methane emission factor for flaring activity in our study was based on 98% combustion efficiency[7]. Nitrous oxide emissions during flaring were calculated based on an emission factor of 95.2 µg/MJ (HHV basis) for flaring[7]. Sulphur dioxide emissions were calculated based on the typical Montney wellhead composition (**Supplementary Table 9**) and conversion of hydrogen sulphide to sulphur dioxide in the flare. The emission factor for nitrogen oxides was based on the US EPA emission factor for flares[30].

Flared gas volume during completions operations for new wells (**Supplementary Table 4**) was based on public data reported for the BC Montney wells sampled in this study[13]. Most of these emissions were associated with initial flowback of the wells following hydraulic fracturing, but we also included any flaring reported during subsequent workovers.

1.4.5 Gas composition

The Montney formation in BC is split into two reporting areas ("Northern" and "Heritage") which have similar typical gas compositions (**Supplementary Table 9**). We assumed the average of the two areas for all raw Montney NG production. The production scenario based on average BC marketable NG used an average of published NG compositions for the transmission pipelines in BC[32] weighted by sales volumes in each pipeline[20]. For the scenarios assessing processing emissions for Montney NG, condensate composition (**Supplementary Table 10**) was assumed based on the average of reported data for a sample of typical wells drilled in 2020.

Supplementary Table 9. Gas compositions assumed in this LCA. Montney gas composition based on the average of the two reporting areas (Heritage and Northern). BC Average is based on production weighted average of marketable NG in the transmission pipelines. Data from refs.[13,20,24,32].

Component	Heritage	Northern	Montney Average	BC Average
Methane	0.8312	0.8134	0.8223	0.8872
Ethane	0.0916	0.0956	0.0936	0.0799
Propane	0.0363	0.0388	0.0376	0.0215
i-Butane	0.0066	0.0074	0.0070	0.0025
n-Butane	0.0103	0.0118	0.0110	0.0029
i-Pentane	0.0029	0.0037	0.0033	0.0005
n-Pentane	0.0032	0.0037	0.0034	0.0004
Hexane	0.0029	0.0035	0.0032	0.0002
Heptane	0.0100	0.0100	0.0100	0
CO_2	0.0018	0.0025	0.0022	0.0026
Hydrogen sulphide	0.0008	0.0022	0.0015	0.0022
Nitrogen	0.0021	0.0070	0.0046	0
Hydrogen	0.0001	0.0004	0.0003	0

Supplementary Table 10. Condensate composition assumed in this LCA. Montney condensate composition based on the average of a sample of typical wells drilled in 2020. Data from ref.[13,24].

Component	Mole fraction
Methane	0.1226
Ethane	0.0656
Propane	0.0776
i-Butane	0.0289
n-Butane	0.0636
i-Pentane	0.0390
n-Pentane	0.0457
Hexane	0.0795
Heptane	0.4768
CO_2	0.0004
Hydrogen sulphide	0.00002
Nitrogen	0.0003

1.4.6 EUR estimation

EUR is a key parameter in calculating emission and impact intensity for well drilling and completion operations. We forecasted EUR (NG, condensate, and water) for each well sampled based on fitting a stretched exponential decline curve through the available monthly production data for each month (i):

$$Q_i = Q_0 \cdot e^{-\left(\frac{i}{\tau}\right)^n}$$

where parameters Q_0, n, and τ were adjusted for each well to minimise the residual sum of squares between the available production data and predicted decline curve. Production data in the first two months, or that was impacted by operating restrictions (e.g., downhole chokes or flush production after shut-in period), were excluded from the curve fits to optimise interpolation of unrestricted production data. EUR was determined for each well from the reported cumulative production to date plus the total of forecast future monthly production based on the decline curve for 20 years from first production or until the forecast production rate fell below 3 E3m^3/d, whichever occurred first.

EURs for NG and condensate (liquid hydrocarbons) were estimated by fitting a stretched exponential decline curve through the available monthly production data. Stretched exponential decline curves have demonstrated a good fit for historical Montney production data[27,33]. The mean NG EUR used in this study (146 E6m^3) is consistent with average NG EUR published by the BC Energy Regulator for Montney wells drilled in 2020 (c. 143 E6m^3)[34]. The mean condensate EUR used in this study is within the range of average condensate EURs published by the BC Energy Regulator for Montney wells[34]. We allocated life cycle inventory to the two product streams on an energy basis.

Eight wells drilled in the BC Montney between 2007 and 2015 were checked in this study to compare the forecast production after 28 months based on extrapolating a stretched exponential curve with actual long-term production data. In the example shown in **Supplementary Figure 2**, the forecast EUR based on the first 28 months of production data is 136 E6m3 compared to 149 E6m3 based on 122 months of production data. In this example, the cumulative produced NG at 122 months is 105 E6m3 and the production rate after 20 years is forecast to be 10 E3m3/d. The average EUR at 28 months for the eight historical wells that were checked in this study was 139 E6m^3 compared to 155 E6m^3 (+12%) based on all data available for each of the wells (72-152 months).

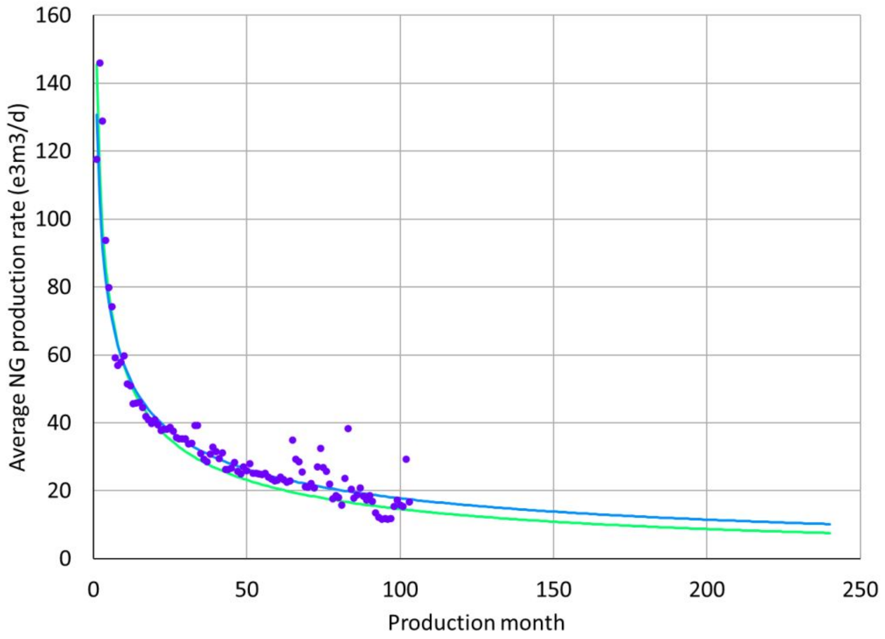

Supplementary Figure 2. Example of decline curve analysis used in this study to estimate EUR. NG production data (purple dots) for BC Montney well drilled in 2012 with stretched exponential decline curves fit through first 28 months of production data (green line) and all available production data (blue line). Data from ref.[13].

1.4.7 LUC

We assumed land use per well of 0.80 ha based on typical existing well pad size with 30 wells per pad, 30 m wide pipeline right-of-way, and 15 m wide road right-of-way[13,31] (**Supplementary Table 11**). Our estimated land use for NG extraction and processing is higher than average US wells estimated by NETL[35] (0.6 ha/well). Our land occupation estimate for wellsites is close to a prior estimate of Montney wells in Alberta (0.46 ha/well)[27]. Jordaan et al. (2017)[36] found significantly higher land occupation for NG production from Barnett shale NG wells (Texas, USA), but their analysis was based on 2009 data with 73% of the wells located on single well pads and 99% of the wells located on pads with less than 4 wells. Their sensitivity analysis showed a strong negative correlation between average land occupation per well and number of wells per pad, but they did not consider well density comparable to typical Montney well pad layouts.

Supplementary Table 11. Breakdown of land use assumption for NG wells.

	Area (ha/well)
Wellsite	0.5
Pipeline	0.2
Access road	0.1

1.4.8 Abandonment

We assumed that wells are abandoned by setting cement plugs (5 m^3/well assumed) in the wellbore casing and then cutting and capping in accordance with well decommissioning guidelines for BC[37]. We assumed production tubing is removed and recycled along with wellhead and surface piping/structural/equipment (10 t/well total assumed). We assumed support piles are cut below grade. Pipelines were assumed to be abandoned in place with risers cut and capped below grade. We assumed that abandonments are completed as part of a large, coordinated program so that equipment transport allocation for individual wells is negligible.

1.5 NG processing

1.5.1 Fugitive emissions

Studies measuring fugitive methane emissions associated with oil and NG production in BC[38-40] have found that emission rates in government reported annual GHG inventories were underestimated (2010-2019 data); however, MacKay et al. (2021)[41] found significant variance between fields in western Canada and measured fugitive methane rates within the BC Montney field in 2016 (1.04 gCO_2e/MJ, adjusted for GWP100 of 29.8) that were lower than reported for the industry overall. The BC government significantly increased fugitive methane emission estimates (current and historical) for the oil and NG industry in their 2020 and 2021 GHG emissions inventories[19,42] compared to prior inventories[43]. The new estimates more closely align with the prior studies which were not field specific (refs.[38-40]). The revised estimate of total methane emissions in the 2019 GHG emissions inventory related to upstream oil and NG production in BC[19] is 16% higher than the annual emission rate we calculate with QGIS[44] using

data from the analysis of satellite measurements during May 2018 to February 2020 published by Shen et al.[45] for the region of northeastern BC where upstream oil and gas production is located. We allocated 2020 reported fugitive methane emissions in BC (2.75 MtCO_2e, adjusted for GWP100 of 29.8[19]) on an energy basis (**Supplementary Table 12**) to estimate a baseline fugitive methane intensity of 1.19 gCO_2e/MJ_{LHV} (0.0401 gCH_4/MJ_{LHV}) and assumed that this is representative of new Montney wells. Fugitive emissions of other hydrocarbons are not reported, so we calculate fugitive emission rates for ethane and propane by assuming that these components have leakage rates in proportion to the assumed gas composition for Montney wells (0.00855 gC_2H_6/MJ_{LHV} and 0.00504 gC_3H_8/MJ_{LHV}). This fugitive emission intensity was used for the first three NG production scenarios. Similar methodology applied to 2014 data (2.06 gCO_2e/MJ_{LHV}) resulted in a fugitive methane emission intensity for the fourth scenario of 0.51 gCO_2e/MJ_{LHV} (0.0173 gCH_4/MJ_{LHV}), and proportionate rates for ethane and propane, based on the BC government target to reduce fugitive emissions 75% below 2014 emissions by 2030[46].

Supplementary Table 12. Calculated energy allocations for marketable oil and gas products in BC during 2020. Based on reported production volumes[20] and assumed energy densities (LHV basis)[32,47-49].

Commodity	Energy Density	2020 Production		Energy Allocation
		Volume	Energy (PJ)	
NG	37.5 MJ/m^3	55 446 625 e3m^3	2079	90.2%
Ethane	16.8 GJ/m^3	354 917 m^3	6	0.3%
Propane	23.3 GJ/m^3	2 089 867 m^3	49	2.1%
Butane	25.9 GJ/m^3	1 841 736 m^3	48	2.1%
Pentane+	28.2 GJ/m^3	3 308 728 m^3	93	4.0%
Crude Oil	39.6 GJ/m^3	790 695 m^3	31	1.3%

Baseline fugitive methane emissions in this study are similar to measured values in the Alberta Montney for a producer that had implemented a leak detection and repair program (1.15 gCO_2e/MJ_{LHV}, adjusted to methane GWP100 of 29.8)[26]. Fugitive emissions associated with NG transmission in BC are negligible compared to upstream emissions[19].

1.5.2 Production flaring

Insufficient data were available to disaggregate flaring emissions reported by BC for the oil and NG industry to different aspects of production. Methane emissions from flaring are included in the fugitive methane emission estimate. To estimate CO_2 emissions from flaring during other production operations (e.g., maintenance, shutdowns, process upsets), we deducted our estimate for CO_2 emissions from flaring associated with new wells drilled in BC in 2020 from the total reported CO_2 emissions for flaring in BC in 2020 (0.47 $MtCO_2$)[42] and allocated the balance to NG and liquids production on an energy basis (**Supplementary Table 12**) to obtain an average CO_2 emission factor for flaring associated with on-going production operations of 0.20 gCO_2/MJ_{LHV}. We assume that the composition of flared gas is the same as average Montney well effluent to calculate gas flaring of 3.57 MJ_{LHV}/GJ_{LHV} of produced NG and use the same trace pollutant emission factors as described above for flaring during completion operations (Section 1.4.4).

For the baseline NG processing scenario (average marketable NG in BC), we also included emissions of CO_2 vented from NG processing facilities (1.40 $MtCO_2$ in 2020[42]) allocated on an energy basis (0.61 gCO_2/MJ_{LHV}). For the low-emission Montney NG production scenarios, there is no CO_2 vented from the NG processing equipment by design.

1.5.3 Energy inputs

For the average BC NG supply scenario, we used the BC government reported 2020 GHG inventory[42] and oil and NG production data (**Supplementary Table 12**) to calculate industry average emission factors (CO_2, methane, nitrous oxide) for NG production based on all existing infrastructure and practices (**Supplementary Table 13**). Emission factors for other criteria air pollutants are based on the government of Canada air pollutant emissions inventory[50]. This scenario is intended to be representative of a CCGT facility which is supplied NG from one of the transmission pipelines in northeast BC near or within the Montney field. We assumed the CCGT is located upstream of any booster compression on the transmission pipeline and that impacts associated with the transmission pipeline are negligible given the presumed short distance from

processing. Stationary combustion emissions associated with drilling and completing new wells were deducted from reported emissions to avoid double-counting.

Supplementary Table 13. Stationary combustion emission intensities assumed for baseline. Based on BC government reported stationary combustion GHG emissions for oil and NG industry[42] and government of Canada reported air pollutant emissions inventory[50] in 2020. Based on reported production volumes[20] and assumed energy densities[32,47-49]. Estimated drilling and completion (D&C) emissions are subtracted from reported emissions to avoid double counting.

Air emission	Reported emissions	D&C emissions	Emission intensity (g/MJ_{LHV})
Total GHG	7.07 $MtCO_2e$	0.20 $MtCO_2e$	2.98
CO_2	6.59 Mt	0.19 Mt	2.78
Methane	14.5 kt	0.53 kt	6.04E-3
Nitrous oxide	0.169 kt	0.002 kt	7.22E-5
Ammonia	34.6 t	-	1.50E-5
Carbon monoxide	20.6 kt	-	8.94E-3
Volatile organic compounds (non-methane)	8.3 kt	-	3.58E-3
Nitrogen oxides	23.2 kt	-	1.01E-2
Sulphur oxides	10.9 kt	-	4.74E-3
Particulate matter (<2.5 µm)	314 t	-	1.36E-4

Electricity consumption by the oil and NG industry in BC is reported together with the mining industry (1679 GWh in 2020)[51], so an accurate estimate of existing electricity consumption specific to NG production is not possible. However, the combined grid-supplied electricity consumption is very small compared to the energy content of NG consumed[51] and the BC grid electricity GHG emission factor is low due to most supply coming from domestic hydroelectric facilities[52] (this analysis uses the Ecoinvent background inventory for BC electricity supply which is higher than the 40.1 $kgCO_2e/MWh$ factor recommended by the BC government for 2020). Therefore, Scope 2 emissions for the baseline scenario associated with electricity supply to the oil and NG industry in BC were neglected as insignificant compared to reported stationary combustion emissions since emissions associated with the total electricity consumption by the oil and NG industry and mining industry combined are less than 1% of the stationary combustion

emissions reported for the oil and NG industry. For the baseline scenario, emissions from self-generated electricity are included in the reported emissions.

For the low-emission NG process scenarios, the NG and electricity consumption of the process were determined from the process model (Supplementary note 2). Electricity was assumed to be supplied from the BC electrical grid and fuel gas was internally supplied from the processed NG stream leaving the processing plant. Compressor engine NO_x emissions were based on the statutory limit in Canada for large stationary NG engines (2.7 gNO_x/bkW-h)[53]. Other engine trace air pollutants were modeled based on the US EPA emission factors for 4-stroke lean burn NG engines[30]. Trace air pollutants from supply of heat to the NG processing plant were modeled based on the Ecoinvent background inventory for "Heat, district or industrial, natural gas (RoW)| heat production, natural gas, at boiler modulating >100kW | Cut-off, U" adjusted for the BC Montney NG composition.

1.5.4 Glycol and amine consumption

The glycol/amine losses in the NG dehydration/sweetening processes due to vaporization were calculated in the process model. The baseline case with average BC production emissions assumed glycol/amine losses were the same as calculated in the process model. Cases with reciprocating engine driven compressors assumed 0.03 g/MJ_{fuel} of engine lubricating oil consumption and disposal based on comparable equipment in the Ecoinvent background inventory database[54].

1.5.5 Production equipment

Life cycle impacts for the NG processing facility construction and operation were estimated using a hybrid LCA approach. The inventory for steel consumption for equipment and construction materials was based on weight estimates provided by the process cost model (2364/2115 t for NG/electric drive compressors) and the combination of Ecoinvent library processes "Steel, low-alloyed, hot rolled (GLO)| market for steel, low-alloyed, hot rolled | Cut-off" plus "Sheet rolling, steel (GLO)| market for sheet rolling, steel | Cut-off". The inventory for steel consumption for supply of annual maintenance materials was estimated as the steel consumption for facility construction multiplied by the ratio of annual maintenance and facility construction cost

estimates from the process cost model (0.93% and 0.73% per year for NG-engine and electric drive scenarios). Facility construction labour, electrical materials, instrumentation, and maintenance labour were modelled using US environmentally extended input-output (EEIO) factors applied to the capital and maintenance cost estimates for the facility (Supplementary note 2.4) disaggregated into representative US EEIO categories (**Supplementary Table 14**). Capital and operating costs associated with engineering design, financing, insurance, taxes, and operating labour were assumed to have negligible environmental impacts. The base year of the process cost model (2018) was adjusted to the base year of the US EEIO database (2017) using the Chemical Engineering Plant Cost Index[55].

Supplementary Table 14. US EEIO categories used to model environmental impacts for the NG processing facility construction, electrical materials, instrumentation, and facility maintenance. Plant construction costs in million USD and annual maintenance costs in million USD per year with Q1 2018 base year.

US EEIO category	NG drive	Electric drive
**Plant construction**		
Other nonresidential structures	$19.4	$19.0
Industrial process variable instruments	$4.6	$4.6
Communication and energy wire and cable	$2.0	$2.7
Switchgear and switchboards	$2.0	$2.7
**Annual maintenance**		
Other nonresidential structures	$0.33	$0.25
Industrial process variable instruments	$0.061	$0.047
Communication and energy wire and cable	$0.024	$0.028
Switchgear and switchboards	$0.024	$0.028

1.5.6 Water disposal

We assumed that one additional well would be drilled on or near the NG processing facility for disposal of produced water and that the well has the same life cycle inventory as a typical Montney well in this study (excluding flaring).

1.5.7 Pipeline releases/spills

Releases from pipelines and spills related to oil and NG industry activities in BC were modelled as emissions to soil based on total release volumes for all oil and NG industry incidents in BC in 2020 (544 m^3 salt water, 5 m^3 emulsion, and 185 m^3 methanol)[56] allocated to production on an energy basis. Salt water composition was based on typical Montney produced water analyses[13]. Emulsion composition was assumed to be 19% petroleum oil and 81% salt water based on ratio of Montney well EUR for condensate and water.

1.5.8 LUC

We assumed 10 ha for the central NG processing facility and 0.5 ha for the water disposal wellsite based on similar existing facilities in the BC Montney[13,31].

1.5.9 Abandonment

We estimate equipment decommissioning LCA inventory based on US EEIO factors using the facility construction cost estimates for construction labour as a proxy for equipment decommissioning costs and do not include credits from associated material recycling or potential reuse. We assumed the abandonment inventory for the water disposal well is the same as production wells except surface piping/structural/equipment was neglected as insignificant.

1.6 Electricity generation

1.6.1 CCGT load profiles

We considered the load profiles from five existing CCGT facilities in North America which have rated output similar to the unit in this study – Shepard (Alberta), Sewaren (New Jersey), Hilltop (Pennsylvania), Wildcat (Maryland), and Greensville (Virginia) – with a wide range of average capacity factors, start-up/shutdown frequency, and load profiles (**Supplementary Table 15** and **Supplementary Figure 3**). The power plants were selected based on rated capacity: the largest CCGT in Alberta (the only facility in Alberta comparable in output to this study)[57] and the four largest facilities in the US EPA Clean Air Markets Program (eastern US)[58]. We used the average of

data reported during 2020-2022 from each facility except Hilltop which was commissioned in 2021 (only 2022 data used). Wildcat and Greensville have multiple units (2 and 3 respectively) with very similar load profiles, so we used the average of the units at each facility. The duration of the start-up sequence for a CCGT depends on the temperature of the equipment at the time of start-up, as the CCGT will gradually cool from operating temperature to ambient during the preceding shutdown. For the purposes of this study, three different start conditions were considered: hot (<8 hours shutdown), warm (8-64 hours shutdown), and cold (>64 hours shutdown)[59-62]. We assumed the small number of operating hours for each facility at less than 35% load were associated with start-up/shutdown cycles.

Supplementary Table 15. Summary of operating parameters for the existing CCGT facilities considered in this study. Data shown is the annual average for 2020-2022, except Hilltop is for 2022 only. Shutdowns and restarts are average number per year. Based on data from refs.[57,58].

Facility	Capacity (MW)	Capacity factor	Shutdowns	Hot restarts	Warm restarts	Cold restarts
Shepard	868	81%	0.3	0	0	0.3
Sewaren	717	53%	18.7	8.0	6.7	4.0
Hilltop	665	73%	18.0	7.0	4.0	7.0
Wildcat	557	37%	104.0	45.7	41.3	17.3
Greensville	591	73%	7.3	1.7	3.0	2.7

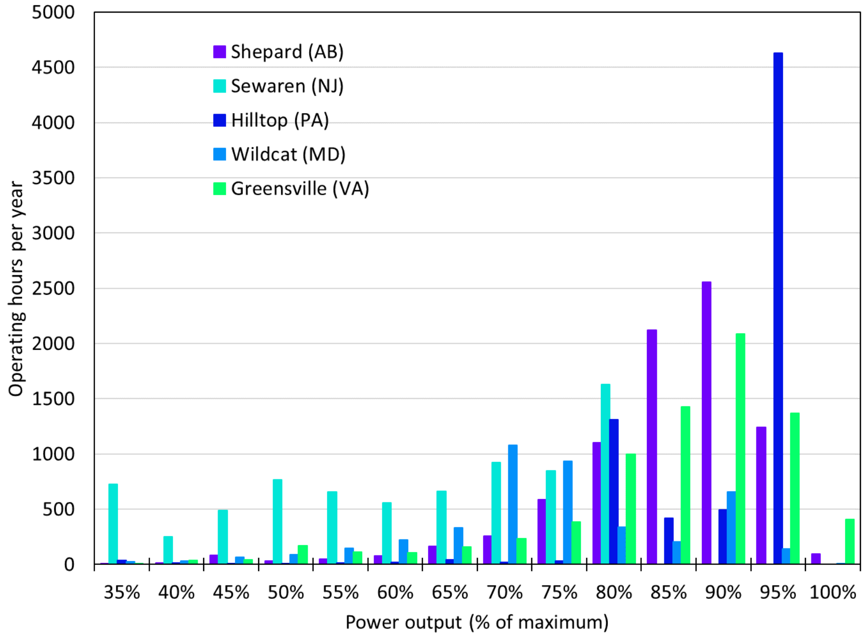

Supplementary Figure 3. Load profiles for the facilities considered in this study. Data shown is the annual average for 2020-2022, except Hilltop is for 2022 only. Based on data from refs.[57,58].

We also considered the effect of potential future CCGT duty cycles where frequent startup/shutdown cycles are required (up to 400 per year) to balance generation and demand on the electricity grid. For these cases, we assumed different distributions of hot/warm/cold startups and that the CCGT operates at 95% of rated output when required to run. The duration of shutdown prior to each hot/warm/cold start in these scenarios was assumed to be 8/36/64 hours and the CCGT was assumed to be unavailable 30 days per year due to maintenance and unplanned outages.

1.6.2 Normal operating inventory

We considered four inventory scenarios for CCGT with CCS in our study corresponding to the three gross-CO_2 capture rates in the NETL baseline study[63] – 90%, 95%, and 97% (net fossil-CO_2 capture rates, excluding CO_2 associated with inlet air, of 90.7%, 95.7%, and 97.7% respectively) – and 98.5% gross capture rate based on the IEAGHG baseline study (99.2% fossil-CO_2 capture)[64] which used the same absorption solvent as the NETL study. Net CCGT power output in the 98.5% capture case was assumed to be 97.89% of the net power output in the 90% capture case based on the IEAGHG baseline study[64].

The NETL and IEAGHG baseline studies provide performance and emissions data for operation at maximum rated output. We adjusted the CCGT part load correlations developed by Spitz et al.[65] to generate inventories for part load operation that are aligned with the performance data at maximum output in the NETL baseline study (**Supplementary Table 16**). We increased net direct CO_2 emissions in the NETL baseline study by 2% to account for the marginally higher carbon content of the NG composition in this study and ignore effects of this composition change on other life cycle inventory data.

Trace air pollutant inventory from CCGT combustion was modelled using factors from the Ecoinvent library process "Electricity, high voltage (CA-BC)| electricity production, natural gas, combined cycle power plant" with the modifications described below to nitrogen oxide, sulphur dioxide, and particulate emission rates. Carbon monoxide, sulphur oxides, and particulate emissions for the state-of-the-art H-class gas turbine with CCS were assumed to be negligible during normal operation[63]. The NO_x emission factor (0.01 kg/MWh) was based on an ultra-low NO_x burner with catalytic reduction per the assumptions in the NETL study[63]. Ammonia/amine emissions in the flue gas exiting the absorber are not included in the NETL baseline study[63], NETL CCGT LCA[35], or the background Ecoinvent inventory for CCGT without CCS. We assumed the contactor water wash section reduces ammonia concentration in the flue gas to 5 ppmv and amine concentration to a negligible residual based on pilot test data[66,67].

Supplementary Table 16. CCGT NG consumption, electricity output, and air emissions inventory for normal operation assumed in this study. CCGT output is percent of maximum for each CO_2 capture scenario. Net CO_2 emitted excludes CO_2 that entered the CCGT with the inlet air. Calculated using data from refs.[30,63,68] and part load correlations from Spitz et al.[65].

**CCGT output**	**35%**	**40%**	**45%**	**50%**	**55%**	**60%**	**65%**	**70%**	**75%**	**80%**	**85%**	**90%**	**95%**	**100%**
NG consumption (GJ_{LHV}/h)	2492	2775	3048	3315	3576	3834	4087	4340	4590	4841	5092	5345	5598	5854
Electricity output (MW)														
90% capture	309	353	397	442	486	530	574	618	662	706	751	795	839	883
95% capture	307	351	395	439	482	526	570	614	658	702	745	789	833	877
97% capture	306	349	393	437	480	524	567	611	655	698	742	786	829	873
98.5% capture	303	346	389	432	475	519	562	605	648	691	735	778	821	864
Net CO_2 emissions (t/h)														
90% capture	13.3	14.8	16.2	17.7	19.1	20.4	21.8	23.1	24.5	25.8	27.2	28.5	29.9	31.2
95% capture	6.11	6.80	7.47	8.13	8.77	9.40	10.0	10.6	11.3	11.9	12.5	13.1	13.7	14.4
97% capture	3.24	3.60	3.96	4.31	4.64	4.98	5.31	5.63	5.96	6.28	6.61	6.94	7.27	7.60
98.5% capture	1.08	1.20	1.32	1.44	1.55	1.66	1.77	1.88	1.99	2.09	2.20	2.31	2.42	2.53
Other air emissions (kg/h)														
Nitrogen oxides	5.1	5.4	5.7	6.1	6.4	6.7	7.0	7.4	7.7	8.0	8.4	8.7	9.0	9.4
Ammonia	7.9	8.4	8.9	9.4	9.9	10.4	10.9	11.4	11.9	12.4	13.0	13.5	14.0	14.6

Consumable inventory for the CCGT with CCS (**Supplementary Table 17**) was taken from the NETL baseline study[63]. Consumable inventory trends versus capture rate from the NETL study were extrapolated to include the 98.5% capture case where necessary. We assumed that consumption rates at part load scale linearly with electrical output. Materials for the SCR catalyst and CO_2 absorbent are not specified. We modeled impacts for the SCR catalyst based on the operating cost estimate and US EEIO factors using the category "Other basic inorganic chemicals, at manufacturer". There is limited long-term test data on amine solvent consumption rates for post-combustion CO_2 capture with CCGT and significant variability in reported data (c. 0.3-3.6 kgMEA/tCO_2 captured)[66,67]. Impacts associated with CO_2 absorbent supply in this study were modelled using monoethanolamine with a consumption rate of 2 kgMEA/tCO_2 captured[69] (near the midpoint of the range of values reported in pilot testing[66]) as a proxy for the proprietary absorbent assumed in the NETL study. Water treatment chemicals were modelled using the Ecoinvent process "Sodium chloride, powder (GLO)| market for sodium chloride, powder". Waste disposal for absorbent reclaimer and triethylene glycol were modelled using the Ecoinvent waste treatment processes "Refinery sludge (RoW)| market for refinery sludge" and "Spent antifreezer liquid (GLO)| market for spent antifreezer liquid" respectively.

Supplementary Table 17. CCGT consumable inventory assumed in this study. Calculated using data from the NETL baseline study[63]. 2018 base year for consumable costs.

**Capture rate scenario**	**90%**	**95%**	**97%**	**98.5%**
<u>Consumables</u>				
Water (m^3/MWh)	0.958	0.978	0.997	1.021
Water treatment chemicals (kg/MWh)	0.522	0.530	0.537	0.540
Ammonia 19%wt. (kg/MWh)	0.199	0.200	0.201	0.203
SCR catalyst (USD/MWh)	2.92E-2	2.94E-2	2.95E-2	2.98E-2
CO_2 absorbent (kgMEA/tCO_2)	2	2	2	2
Triethylene glycol (kg/MWh)	0.115	0.123	0.126	0.129
<u>Waste disposal</u>				
Absorbent reclaimer (kg/MWh)	0.100	0.105	0.110	0.116
Triethylene glycol (kg/MWh)	0.115	0.123	0.126	0.129

1.6.3 Startup and shutdown inventory

We considered a base case where the amine regenerator is heated up to operating temperature before amine circulation begins and a CO_2 capture system designed with segregated lean and rich amine solvent storage to capture CO_2 from the CCGT exhaust gas during startup prior to the amine regenerator reboiler reaching operating temperature as described in ref.[59]. Stored lean solvent is pumped through the contactor to separate rich solvent storage. Sufficient lean/rich solvent storage volume is included for the amine solvent flow rate through the absorber to be sustained until the regenerator reaches operating temperature (**Supplementary Figure 4**). Rich solvent stored during the startup sequence is then regenerated while the CCGT operates at 50% load on the gas turbine using throttling control between the intermediate pressure and low pressure steam turbines to increase amine regeneration up to the rated capacity of the regenerator as described in Spitz et al.[65]. Amine solvent from the rich solvent storage tank is blended with rich solvent leaving the absorber to go to the regenerator in this operating mode (**Supplementary Figure 5**). Amine solvent leaving the regenerator in excess of the inlet rate to the absorber refills the lean amine storage tank to prepare for the next startup. Once the stored amine has been regenerated, the CCGT begins normal operation and ramps to the desired electrical output.

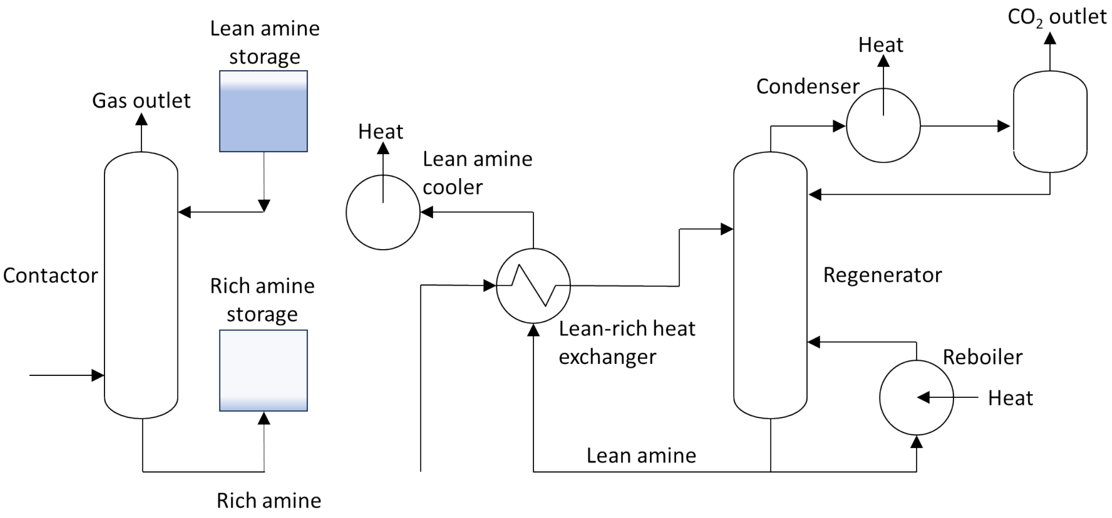

Supplementary Figure 4. CO_2 capture process operating with interim solvent storage to mitigate startup emissions. Stored lean solvent is pumped through the contactor to separate rich solvent storage until regenerator reaches operating temperature.

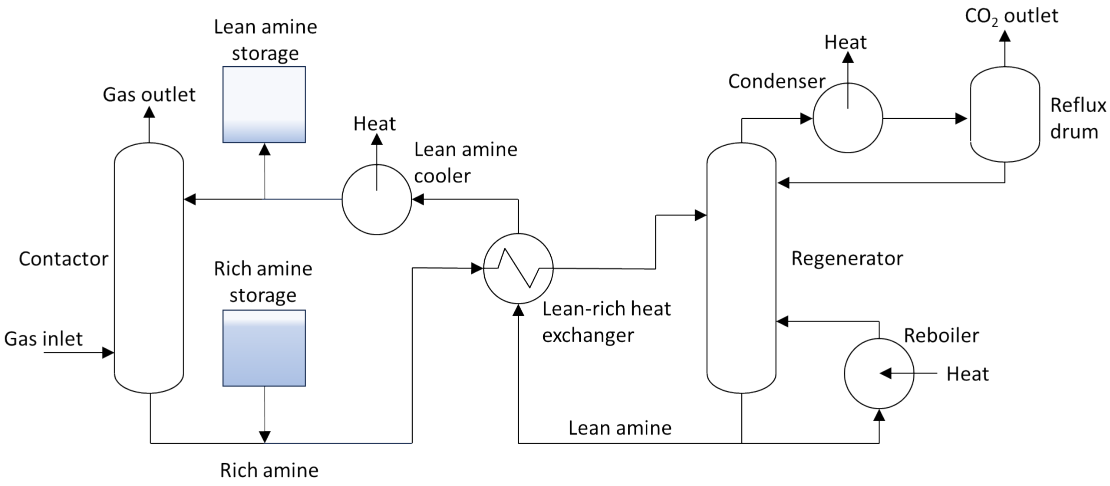

Supplementary Figure 5. CO_2 capture process operating in mode to regenerate stored rich solvent. Excess regenerated solvent refills the lean amine storage tank.

The startup sequences for each of the three start conditions considered in this study were based on refs.[59-62] (**Supplementary Table 18**, **Supplementary Table 19**, and **Supplementary Table 20**). We assumed power output in steps 1 and 2 from only the gas turbine generator and used the average output for periods where output is ramped (linear ramp assumed). We assumed that amine solvent flow rate is adjusted to maintain the design capture rate and lean/rich amine

loading as described in Spitz et al.[65]. Details on the CO_2 absorption system were not provided in the NETL baseline study. Therefore, we followed the methodology of ref.[59] to estimate the required energy input to heat the regenerator reboiler to operating temperature. The rate of energy provided to the regenerator reboiler from the CCGT steam system is based on the methodology from ref.[59] using part load performance correlations described in Spitz et al.[65]. The NETL baseline study does not discuss amine solvent storage for CO_2 capture during start-up, so we assumed that the solvent required to accommodate this is incremental to the cost estimate and quantified the mass required based on the cold startup scenario. We used monoethanolamine in the life cycle impact calculations as a proxy for the proprietary solvent in the NETL baseline study and accounted for additional storage tanks based on material estimates calculated using Aspen Process Economic Analyzer[70].

Supplementary Table 18. Description of hot startup sequence assumed in this study.

Step	Description	Steam status	Gas Turbine Load	Duration (min)
1	Ignition, ramp gas turbine load	Not available	0-50%	15
2	Hold gas turbine load, preheating steam generator	Not available	50%	10
3	Ramp steam turbine load, preheat amine solvent regenerator reboiler	Available	50%	10
4	Hold CCGT load, regenerate loaded amine solvent stored during startup	Available	50%	52

Supplementary Table 19. Description of warm startup sequence assumed in this study.

Step	Description	Steam status	Gas Turbine Load	Duration (min)
1	Ignition, ramp gas turbine load	Not available	0-50%	15
2	Hold gas turbine load, preheating steam generator	Not available	50%	25
3	Continue steam generator preheat, preheat amine solvent regenerator reboiler	Available	50%	20
4	Ramp steam turbine load, regenerate loaded amine solvent stored during startup	Available	50%	25
5	Hold CCGT load, regenerate loaded amine solvent stored during startup	Available	50%	70

Supplementary Table 20. Description of cold startup sequence assumed in this study.

Step	Description	Steam status	Gas Turbine Load	Duration (min)
1	Ignition, ramp gas turbine load	Not available	0-50%	15
2	Hold gas turbine load, preheating steam generator	Not available	50%	45
3	Continue steam generator preheat, preheat amine solvent regenerator reboiler	Available	50%	20
4	Ramp steam turbine load, regenerate loaded amine solvent stored during startup	Available	50%	40
5	Hold CCGT load, regenerate loaded amine solvent stored during startup	Available	50%	90

During startup sequences, unburned methane in the exhaust is higher than normal operation[30,71,72], but published data are very limited. We used the emission intensity measured by Hajny et al.[68] during startup of a commercial CCGT facility (417 gCH_4/GJ_{HHV}), but acknowledge that this represents a single datapoint and that further study is needed to better quantify emissions for a range of turbine designs and operating conditions. Unburned methane emission intensity was assumed at the startup rate until steam becomes available from the heat recovery steam generator (end of step 2) based on estimated durations of startup sequences prior to meeting emissions compliance in regulatory applications for similar H-class CCGT facilities[61,62]. The operating emission intensity was used for the remaining duration of the startup sequence.

Emissions of criteria air pollutants (nitrogen oxides, carbon monoxide, and non-methane volatile organic compounds) during startup (until steam available) and shutdown events were estimated based on published estimates in regulatory applications for similar H-class CCGT facilities[61,62]. Values were scaled linearly based on the rated output capacity of the combustion turbine. Part-load emission rates for nitrogen oxides and non-methane volatile organic compounds after steam availability were calculated assuming flue gas concentration is the same as full load[71]. Nitrous oxide emissions during startup and shutdown were estimated based on the emission factor for normal operation. The selective catalytic reduction system on the turbine exhaust was assumed to be inactive until the availability of steam. Therefore, ammonia emissions were assumed to be

zero up to that point and then were calculated using the normal operation flue gas concentration. Based on these assumptions, inventories for hot, warm, cold startups were estimated for each CO_2 capture rate scenario (**Supplementary Table 21, Supplementary Table 22, and Supplementary Table 23**).

Start-up sequences for baseline scenarios without solvent storage were also developed for comparison. In these cases, amine solvent circulation was assumed to begin when the regenerator reboiler reached operating temperature and the CO_2 capture system began normal operation. Startup duration and associated life cycle inventories (**Supplementary Table 24, Supplementary Table 25, and Supplementary Table 26**) were reduced as there is no stored inventory of rich amine solvent to regenerate. For hot starts without solvent storage, the life cycle inventories for all capture cases were identical because the regenerator reboiler reaches operating temperature at the same time as the steam turbine finishes ramping up in the assumed startup sequence.

Supplementary Table 21. CCGT life cycle inventory for hot startups with solvent storage assumed in this study. Net CO_2 emitted excludes CO_2 that entered the CCGT with the inlet air. Values shown are per startup.

**Capture rate scenario**	**90%**	**95%**	**97%**	**98.5%**
NG consumption (GJ_{LHV})	5064	5064	5064	5064
Electricity produced (MWh)	562	559	557	552
Air emissions				
Net CO_2 (t)	27.0	12.4	6.6	2.2
Methane (t)	0.56	0.56	0.56	0.56
Nitrogen oxides (kg)	54.0	54.0	54.0	54.0
Volatile organic compounds (non-methane, kg)	10.6	10.6	10.6	10.6
Ammonia (kg)	12.2	12.2	12.2	12.2
Carbon monoxide (kg)	84.5	84.5	84.5	84.5

Supplementary Table 22. CCGT life cycle inventory for warm startups with solvent storage assumed in this study. Net CO_2 emitted excludes CO_2 that entered the CCGT with the inlet air. Values shown are per startup.

Capture rate scenario	90%	95%	97%	98.5%
NG consumption (GJ_{LHV})	9304	9304	9304	9304
Electricity produced (MWh)	997	993	989	983
Air emissions				
Net CO_2 (t)	49.6	22.8	12.1	4.0
Methane (t)	0.99	0.99	0.99	0.99
Nitrogen oxides (kg)	104	104	104	104
Volatile organic compounds (non-methane, kg)	15.0	15.0	15.0	15.0
Ammonia (kg)	19.6	19.6	19.6	19.6
Carbon monoxide (kg)	109	109	109	109

Supplementary Table 23. CCGT life cycle inventory for cold startups with solvent storage assumed in this study. Net CO_2 emitted excludes CO_2 that entered the CCGT with the inlet air. Values shown are per startup.

Capture rate scenario	90%	95%	97%	98.5%
NG consumption (GJ_{LHV})	12 695	12 695	12 695	12 695
Electricity produced (MWh)	1359	1353	1349	1340
Additional amine inventory required (t)	1660	1753	1789	1817
Air emissions				
Net CO_2 (t)	67.7	31.1	16.5	5.5
Methane (t)	1.56	1.56	1.56	1.56
Nitrogen oxides (kg)	202	202	202	202
Volatile organic compounds (non-methane, kg)	49.0	49.0	49.0	49.0
Ammonia (kg)	25.5	25.5	25.5	25.5
Carbon monoxide (kg)	557	557	557	557

Supplementary Table 24. CCGT life cycle inventory for hot startups without solvent storage assumed in this study. Net CO_2 emitted excludes CO_2 that entered the CCGT with the inlet air. Values shown are per startup.

Capture rate scenario	All
NG consumption (GJ_{LHV})	1840
Electricity produced (MWh)	165
Air emissions	
Net CO_2 (t)	105
Methane (t)	0.56
Nitrogen oxides (kg)	48.3
Volatile organic compounds (non-methane, kg)	7.0
Ammonia (kg)	3.4
Carbon monoxide (kg)	84.5

Supplementary Table 25. CCGT life cycle inventory for warm startups without solvent storage assumed in this study. Net CO_2 emitted excludes CO_2 that entered the CCGT with the inlet air. Values shown are per startup.

Capture rate scenario	90%	95%	97%	98.5%
NG consumption (GJ_{LHV})	4921	4921	4921	4921
Electricity produced (MWh)	472	471	470	469
Air emissions				
Net CO_2 (t)	202	198	196	194
Methane (t)	0.99	0.99	0.99	0.99
Nitrogen oxides (kg)	96.5	96.5	96.5	96.5
Volatile organic compounds (non-methane, kg)	10.1	10.1	10.1	10.1
Ammonia (kg)	7.6	7.6	7.6	7.6
Carbon monoxide (kg)	109	109	109	109

Supplementary Table 26. CCGT life cycle inventory for cold startups without solvent storage assumed in this study. Net CO_2 emitted excludes CO_2 that entered the CCGT with the inlet air. Values shown are per startup.

Capture rate scenario	90%	95%	97%	98.5%
NG consumption (GJ_{LHV})	7077	7077	7077	7077
Electricity produced (MWh)	690	689	688	686
Additional amine inventory required (t)	0	0	0	0
Air emissions				
Net CO_2 (t)	277	270	267	265
Methane (t)	1.56	1.56	1.56	1.56
Nitrogen oxides (kg)	193	193	193	193
Volatile organic compounds (non-methane, kg)	42.9	42.9	42.9	42.9
Ammonia (kg)	10.2	10.2	10.2	10.2
Carbon monoxide (kg)	557	557	557	557

We assumed the CCGT shutdown sequence begins after the gas turbine output has been reduced to 40% of maximum load. First, the steam turbine load is ramped to zero, followed by the gas turbine load ramping to zero, and then termination of NG supply (**Supplementary Table 27**). We assumed that amine continues to circulate through the absorber during the shutdown sequence and that excess steam is available to continue operation of the amine regenerator. As in the startup sequences, we assumed that amine solvent flow rate is adjusted to maintain the design capture rate and lean/rich amine loading as described in Spitz et al.[65]. There is a lack of data quantifying methane emissions during gas turbine shutdown sequences, but evidence that gas turbine combustion efficiency is significantly impaired at low flow rates (c. <35% of maximum design)[30,71-73]. Unburned methane in the exhaust stream was estimated at normal operating emission intensity while the gas turbine is operating at 40% load and the startup emission intensity based on Hajny et al.[68] while the gas turbine is ramping down to zero load. Life cycle inventories for a shutdown were estimated for each CO_2 capture rate scenario using these assumptions (**Supplementary Table 28**).

Supplementary Table 27. Description of shutdown sequence assumed in this study.

Step	Description	Steam status	Gas Turbine Load	Duration (min)
1	Hold gas turbine load, ramp steam turbine load to zero	Available	40%	10
2	Ramp gas turbine load to zero	Available	40-0%	20
3	Shut-off NG supply	Not available	0%	0

Supplementary Table 28. CCGT life cycle inventory for shutdowns assumed in this study. Net CO_2 emitted excludes CO_2 that entered the CCGT with the inlet air. Values shown are per shutdown.

**Capture rate scenario**	**90%**	**95%**	**97%**	**98.5%**
NG consumption (GJ_{LHV})	1076	1076	1076	1076
Electricity produced (MWh)	102	102	102	102
Air emissions				
Net CO_2 (t)	5.7	2.6	1.4	0.5
Methane (t)	0.25	0.25	0.25	0.25
Nitrogen oxides (kg)	6.0	6.0	6.0	6.0
Volatile organic compounds (non-methane, kg)	19.0	19.0	19.0	19.0
Ammonia (kg)	5.1	5.1	5.1	5.1
Carbon monoxide (kg)	87.4	87.4	87.4	87.4

1.6.4 LUC

We assumed a site area of 40 ha for the CCGT facility including CO_2 capture[63].

1.6.5 Capital and operating/maintenance expenses

Similar to Barbera et al.[74], the environmental impacts associated with CCGT construction were estimated by scaling the Ecoinvent library process "Gas power plant, combined cycle, 400MW electrical (RoW)| gas power plant construction, combined cycle, 400MW electrical" based on net electrical output of the CCGT without CCS. Insufficient detail was provided in the NETL study to develop an inventory of materials required for CO_2 capture, compression, and dehydration. The impacts associated with supply of materials required for the construction of this equipment were

modelled based on the material inventory provided in a front-end engineering design study for retrofit of CCS on a 758 MW CCGT power plant (**Supplementary Table 29**)[69] scaled by the ratio of design CO_2 capture rate. Impacts associated with on-site construction of the CCS equipment were modelled based on US EEIO factors and the NETL capital cost estimates (**Supplementary Table 30**). The base year of the NETL baseline study cost estimates (2018) and 758 MW retrofit engineering study (2021) were adjusted to the base year of the US EEIO database (2017) using the Chemical Engineering Plant Cost Index[55]. Component weights for the absorber and regenerator from the 758 MW CCGT retrofit study[69] are similar to the corresponding weight estimates for a 555 MW CCGT in Fadeyi et al.[75]; however, the overall material estimates in the 758 MW CCGT retrofit study[69] are significantly higher than Fadeyi et al.[75] and are believed to be the result of a more comprehensive engineering study.

Supplementary Table 29. Construction material inventory for CO_2 capture, compression, and dehydration equipment for 758 MW CCGT. Data from the Panda Sherman front-end engineering design study[69] with 2021 base year (USD) for EEIO categories.

Item	Amount
**Ecoinvent material quantities**	
Steel, low-alloyed, hot rolled (GLO)| market for	2005 t
Iron-nickel-chromium alloy (GLO)| market for	1495 t
Sheet rolling, steel (GLO)| market for sheet rolling, steel	3500 t
Titanium (GLO)| market for	5 t
Concrete, normal strength (RoW)| market for	8659 m^3
Monoethanolamine (RoW)| ethanolamine production	441 t
**US EEIO category**	
Switchgear and switchboards	$8.90 MM
Communication and energy wire and cable	$8.90 MM
Industrial process variable instruments	$13.3 MM

Supplementary Table 30. Construction cost estimates for CO_2 capture, compression, and dehydration equipment assumed in this study. Based on data from the NETL baseline study[63] with 2018 base year (million USD).

US EEIO category	90% capture	95% capture	97% capture	98.5% capture
Other nonresidential structures	$133.4	$138.1	$141.3	$144.7

Maintenance materials (excluding fuel and consumables) and labour inventories were based on the NETL baseline study cost estimates[63]. Annual maintenance material inventories were estimated as 2.4% of the construction material inventories based on the ratio of the annual maintenance material and the construction material cost estimates. Maintenance labour impacts were modelled using US EEIO factors (**Supplementary Table 31**). Impacts associated with operating labour, taxes, and insurance were neglected as insignificant.

Supplementary Table 31. Maintenance labour cost estimates for CCGT with CCS assumed in this study. All amounts in million USD/year with 2018 base year. Based on data from the NETL baseline study[63].

US EEIO category	90% capture	95% capture	97% capture	98.5% capture
Other nonresidential structures	$16.9	$17.1	$17.1	$17.1

1.6.6 Abandonment

We estimate equipment decommissioning LCA inventory using US EEIO factors for “Other nonresidential structures” based on the facility construction labour cost estimate from the NETL study[63] as a proxy for equipment decommissioning costs and do not include credits from associated material recycling or potential reuse.

1.7 CO_2 sequestration

We assumed that surface equipment required at the CO_2 disposal wells has negligible life cycle inventory compared to the CO_2 pipelines and other inventories in this study. Life cycle impacts

for the CO_2 pipeline were modelled based on a 323.8 mm x 11.1 mm steel pipe. This standard pipe size was selected to provide reasonable pressure drop for the design CO_2 flow rate over 50 km and sufficient wall thickness to meet design criteria for pressure containment with allowances for corrosion and thermal expansion stress. We assumed that CO_2 sequestration infrastructure requirements do not materially change between the different cases based on the relatively small differences in flow rates.

We used Ecoinvent library processes to provide inventories for the installation of the CO_2 pipeline:

- "Steel, low-alloyed, hot rolled (GLO)| market for steel, low-alloyed, hot rolled| Cut-off, S" plus "Drawing of pipe, steel (GLO)| market for drawing of pipe, steel | Cut-off, S" for steel pipe (total of 85.8 t per km);
- "Welding, arc, steel (RoW)| welding, arc, steel| Cut-off, S" for field joint welds assuming 18 m linepipe sections (total of 56.5 m per km);
- "Pitch (RoW)| market for pitch| Cut-off, S" for steel pipe coating adhesive (total of 244 kg per km);
- "Polyethylene, high density, granulate (GLO)| market for polyethylene, high density, granulate | Cut-off, S" for steel pipe external coating (total of 1.02 t per km);
- "Excavation, skid-steer loader (RoW)| excavation, skid-steer loader| Cut-off, S" for topsoil removal (0.3 m deep by 10 m wide) along right-of-way (soil volume was doubled to account for removal and replacement);
- "Excavation, hydraulic digger (RoW)| excavation, hydraulic digger| Cut-off, S" for excavation of trench (1.7 m deep x 1 m wide) along right-of-way (soil volume was doubled to account for removal and replacement);
- "Machine operation, diesel, >= 74.57 kW, low load factor (GLO)| machine operation, diesel, >= 74.57 kW, low load factor| Cut-off, S" for pipe layers (30 h per km); and
- "Transport, passenger car, large size, petrol, EURO 5 (GLO)| market for transport, passenger car, large size, petrol, EURO 5| Cut-off, S" for personnel transport on location (2000 km per km of pipeline).

We assume that an 8 m wide road is constructed on a 15 m wide right-of-way beside the pipeline right-of-way. Road construction was modelled using the Ecoinvent library process "Road, company, internal (RoW)| road construction, company, internal".

1.7.1 Fugitive emissions

Fugitive emissions from the CO_2 pipeline and associated facilities were calculated assuming leakage of 0.00232 Gg/km-y[76]. CO_2 leakage from the reservoir was assumed to be negligible because monitoring of existing sequestration projects has shown no detectable leakage over decades of operation[76-80].

1.7.2 LUC

We assumed 0.5 ha of disturbed land per disposal wellsite, a 15 m wide pipeline right-of-way, and a 15 m wide access road. Pipeline right-of-way is narrower than the gathering system based on assumption of only one pipeline in the ditch. We make the conservative assumption that the length of new road required is the same as the pipeline (i.e., no re-use of existing roads) but note that, in practice, this is unlikely given the extensive network of roads that already exist in the Montney area in BC[31] combined with the roads associated with the gathering system in this study. For 50 km of pipeline and 5 disposal wells, the total disturbed area is 152.5 ha.

1.7.3 Abandonment

We assumed the abandonment inventory for the CO_2 disposal wells is the same as production wells except surface piping/structural/equipment was neglected as insignificant.

1.8 Life cycle impact assessment (LCIA) methodology

LCIAs were completed in SimaPro[54] using the midpoint indicators available in the ReCiPe 2016 impact assessment method[81]. Global warming characterisation factors were updated to reflect values published in the IPCC sixth assessment report[1]. We used global average characterisation factors for airborne emissions of nitrogen oxides, non-methane hydrocarbons, sulphur oxides, and ammonia to avoid skewing results based on geographical factors and make the results more comparable.

LCIA results per MWh of electricity produced were compared with photovoltaic and wind turbine electricity generation life cycle impacts for BC and western US based on Ecoinvent library processes to provide context for the results:

- "Electricity, high voltage (CA-BC)| electricity production, wind, 1-3MW turbine, onshore | Cut-off, S";
- "Electricity, high voltage (WECC, US only)| electricity production, wind, 1-3MW turbine, onshore | Cut-off, S;
- "Electricity, low voltage (CA-BC)| electricity production, photovoltaic, 570kWp open ground installation, multi-Si | Cut-off, S"; and
- "Electricity, low voltage (WECC, US only)| electricity production, photovoltaic, 570kWp open ground installation, multi-Si | Cut-off, S"

1.8.1 Data quality analysis

Sensitivity analyses were completed to assess how changes to key input data and methodological assumptions affect the LCIA results (**Supplementary Table 32**). 90% confidence intervals for mean values calculated in the Montney well inventory were used for sensitivity of those parameters. For most other parameters ±30% was used to assess sensitivity. CCGT fuel and amine consumption were assessed for lower/higher variance based on judgement of potential variance in those parameters.

Supplementary Table 32. Summary of sensitivity analyses conducted in this study.

Sensitivity	Sensitivity range	Comments
CCGT fuel consumption	±10%	Base CCGT is mature technology. Some uncertainty in efficiency loss due to CO_2 capture (e.g., different solvents).
CCGT amine consumption	±60%	Significant variability in reported data from pilot testing. Lack of data on proprietary solvents.
Fugitive emissions	±30%	Historical variances between reported emissions and independent measurements
CCGT N_2O emissions	±30%	Low confidence documented in published emission factors
Production flaring	±30%	Government reported data
CCGT construction	±30%	Generic inventories from Ecoinvent and engineering studies
Process electrical demand	±30%	Approximately equivalent to ±500 kPa on assumed inlet NG pressure (1000 kPag)
EUR	±15%	90% confidence interval
Process heat demand	±30%	Data from process model based on assumed efficiencies
Completions materials	±40%	90% confidence interval (typical)
Completions fuel	±19%	90% confidence interval
Drilling mud materials	±53%	90% confidence interval (typical)
CCGT CH_4 emissions	±30%	Low confidence documented in published emission factors
Completions flaring	±55%	90% confidence interval
Drilling fuel	±22%	90% confidence interval
Wellbore materials	±6%	90% confidence interval
CCGT other trace pollutants	±30%	Low confidence documented in published emission factors

2 SUPPLEMENTARY NOTE 2 – NG PROCESSING MODEL DETAILS

2.1 Process description

The NG processing facility was modelled in Aspen Hysys (Version 11)[82] with a sequence of connected sub-flowsheets (**Supplementary Figure 6**). NG, condensate, and water from the gathering pipeline flows to a three-phase inlet separator (V-100) (**Supplementary Figure 7**). NG from the inlet separator (V-100) flows to a two-stage compressor (K-210/240) with intercooling/aftercooling (**Supplementary Figure 8**) and then to a regenerative amine absorption plant (**Supplementary Figure 9**). In the amine plant, NG flows up through a trayed absorption column (V-300) countercurrent to monoethanolamine (MEA) solvent which absorbs most of the CO_2 and H_2S from the NG. NG leaving the top of the amine contactor flows through a water wash section (V-305) to remove MEA from the NG stream. MEA leaving the bottom of the contactor flows to a flash vessel (V-310) where a portion of the absorbed gases is released from the MEA. MEA is then heated in heat exchanger E-320 before flowing to the top of the regenerator column (V-330). The regenerator is a distillation column with a bottom reboiler and top condenser. MEA flows down through the trayed regenerator while hot vapour from the reboiler rises. Heat input from the reboiler releases additional CO_2 and H_2S (and water) from the MEA. The condenser cools the vapour leaving the column and recycles condensed water to the top of the column. MEA leaving the regenerator flows through the hot side of exchanger E-320, filtration to remove impurities, MEA/water make-up to replenish process losses, charge pump (P-340), cooler (E-350), and booster pump (E-360) to return to the top of the absorption column (V-300).

NG flows from the amine plant to the dehydration plant (**Supplementary Figure 10**) where the NG is cooled (E-400) to condense water which is removed in a two-phase separator (V-410). The NG then flows up through a trayed glycol absorption column (V-420) countercurrent to triethylene glycol (TEG) solvent which absorbs most of the water from the NG. TEG leaving the bottom of the absorption column is heated up in a heat exchanger (E-425) then flows to a two-phase separator (V-430) where trace hydrocarbons absorbed by the TEG are released as vapour. The TEG continues to the top of the glycol regenerator column (V-440). The regenerator is a distillation column with a bottom reboiler. TEG flows down through the trayed regenerator while

hot vapour from the reboiler rises. Heat input from the reboiler vapourises water from the TEG. Hot vapour leaving the top of the column flows to a condenser (E-475) and two-phase separator (V-480). TEG leaving the regenerator flows down through a stripping column (V-450) countercurrent to dry NG rising to remove additional water from the TEG. NG leaving the top of the stripping column enters the bottom of the regenerator column. TEG leaving the bottom of the stripping column flows through the hot side of exchanger E-425, filtration to remove impurities, TEG/water make-up to replenish process losses, pump (P-460) and cooler (E-470) to return to the top of the absorption column (V-420).

Dehydrated NG from absorption column V-420 flows to the refrigeration plant (**Supplementary Figure 11**) where the NG is cooled below the hydrocarbon dewpoint in the gas-gas heat exchanger (E-500) followed by the gas chiller (E-510). Heat is removed from the cold side of the gas chiller by a propane refrigeration cycle. NG from the gas chiller flows to the low temperature separator (V-520) where hydrocarbon liquids are removed. NG from the low temperature separator returns to the cold side of the gas-gas heat exchanger. Liquid hydrocarbons from the low temperature separator flow through the cold side of heat exchanger E-530 to the top of the stabiliser column (V-540). The stabiliser column is a distillation column with a bottom reboiler. Condensate flows down through the trayed stabiliser while hot vapour from the reboiler rises. Heat input from the reboiler releases low molecular weight hydrocarbons from the condensate to reduce the vapour pressure of the resulting liquid product. Vapour leaving the top of the column flows through the overhead compressor (K-550) to return to the main NG flow downstream of the gas-gas heat exchanger. The NG product stream leaving the refrigeration plant (NG-553) meets typical transmission pipeline quality specifications and a portion of this stream is used to provide fuel gas for the NG processing facility (NG-555). Stabilised liquid leaving the reboiler flows through the hot side of heat exchanger E-530 to the liquid hydrocarbon product outlet. Sales gas from the refrigeration plant (NG-554) is compressed to transmission pipeline pressure in a single stage sales compressor (K-1310) followed by an aftercooler (E-1320) (**Supplementary Figure 12**).

Condensate from the inlet separator (V-100) is processed along with unstabilised condensate streams from other unit operations to reduce the vapour pressure to meet condensate sales

specifications (**Supplementary Figure 13**). Produced water from the dehydration plant is also processed to remove liquid hydrocarbons. The condensate flows to a three-phase separator (V-600) and heat exchanger E-610 to the top of the stabiliser column (V-620). Stabiliser column V-620 is a distillation column with a bottom reboiler. Condensate flows down through the trayed stabiliser while hot vapour from the reboiler rises. Heat input from the reboiler releases low molecular weight hydrocarbons from the condensate. Vapour leaving the top of the column and vapour that flashed off the condensate in V-600 flow to the single-stage recycle compressor (**Supplementary Figure 14**) to return to the suction of the main NG compressor. Stabilised condensate leaving the reboiler flows through the hot side of heat exchanger E-610 to the liquid hydrocarbon product outlet along with the condensate from the refrigeration plant.

The acid gas (CO_2 and H_2S) from the amine plant flows through four stages of compression (**Supplementary Figure 15**) to reach a suitable pressure for the acid gas dehydration plant (**Supplementary Figure 16**). The acid gas dehydration process is similar to the NG dehydration process except that water removed in the inlet scrubber (V-1110) and vapour from the flash separator (V-1140) are recycled to the suction of the acid gas compressor. Water condensing out in the 2nd through 4th stages of the acid gas compressor is also recycled to the suction drum (V-1000) to recover CO_2 and H_2S. Dehydrated acid gas flows through two final stages of compression (**Supplementary Figure 17**) to reach the design pressure for sequestration with the CO_2 from the CCGT.

Produced water from the inlet separator (V-100) and other unit operations flows to a two-phase separator (**Supplementary Figure 18**) to remove vapour which is recycled along with vapour from the NG dehydration plant and acid gas regenerator column to the suction of the recycle compressor through the vapour recovery unit (**Supplementary Figure 19**).

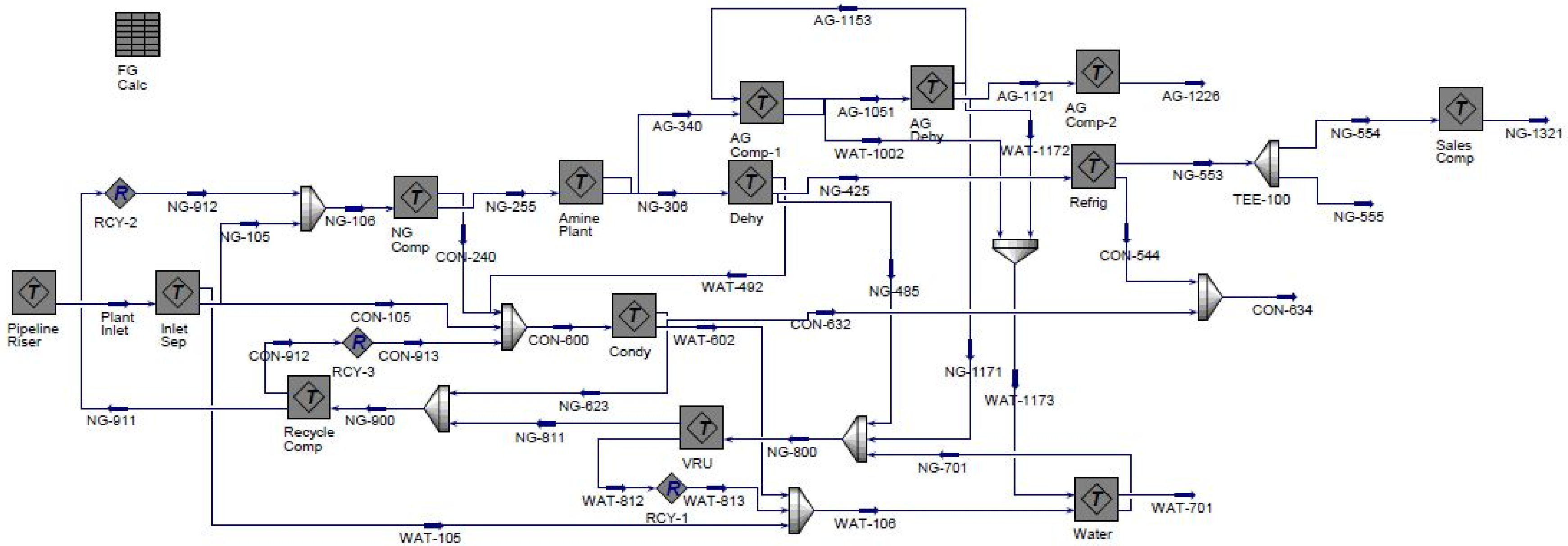

Supplementary Figure 6. Overall process model schematic.

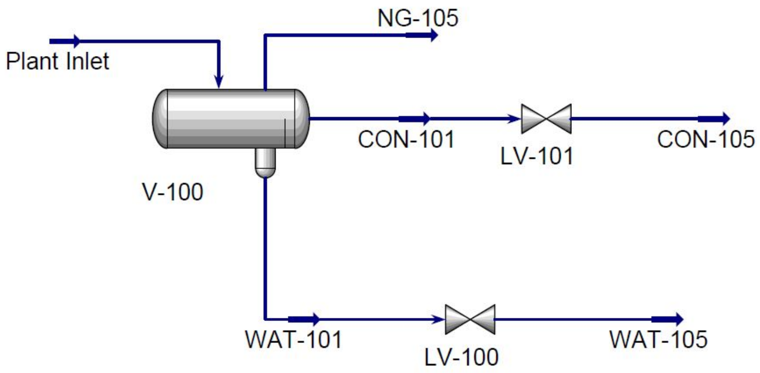

Supplementary Figure 7. Inlet separator model schematic.

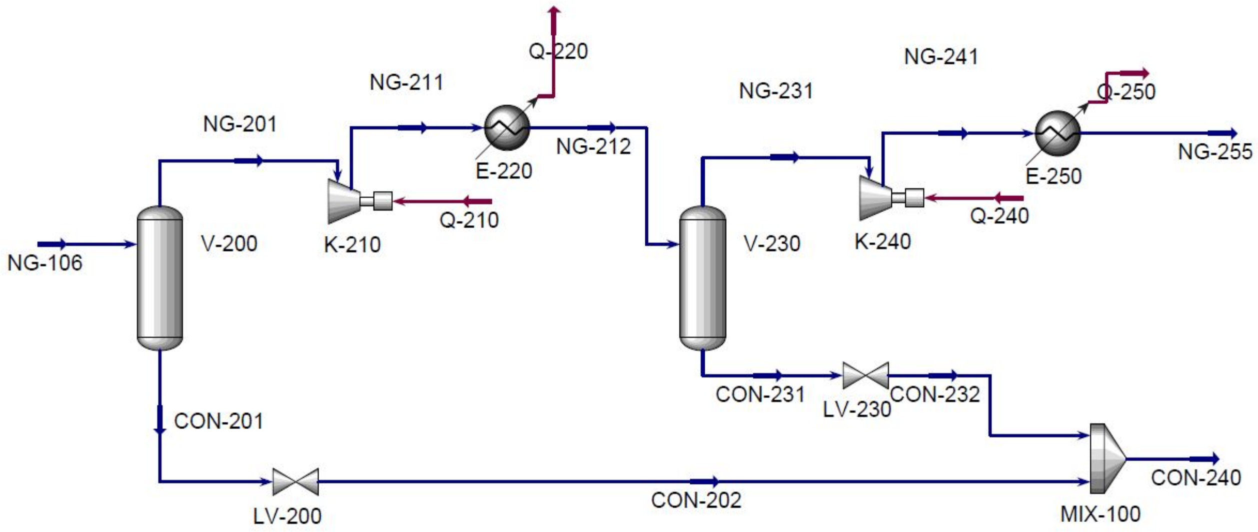

Supplementary Figure 8. NG compressor model schematic.

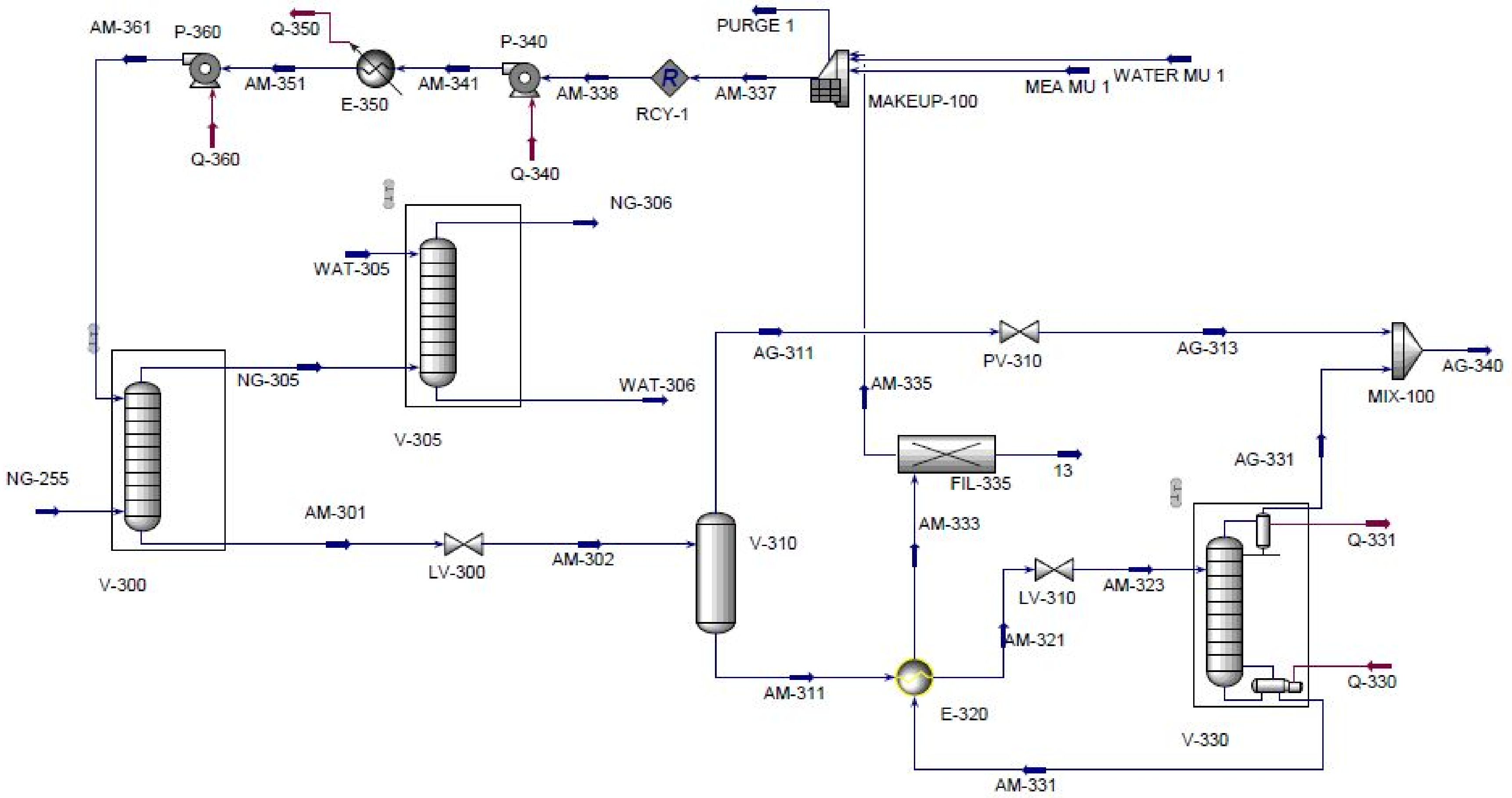

Supplementary Figure 9. Amine plant process model schematic.

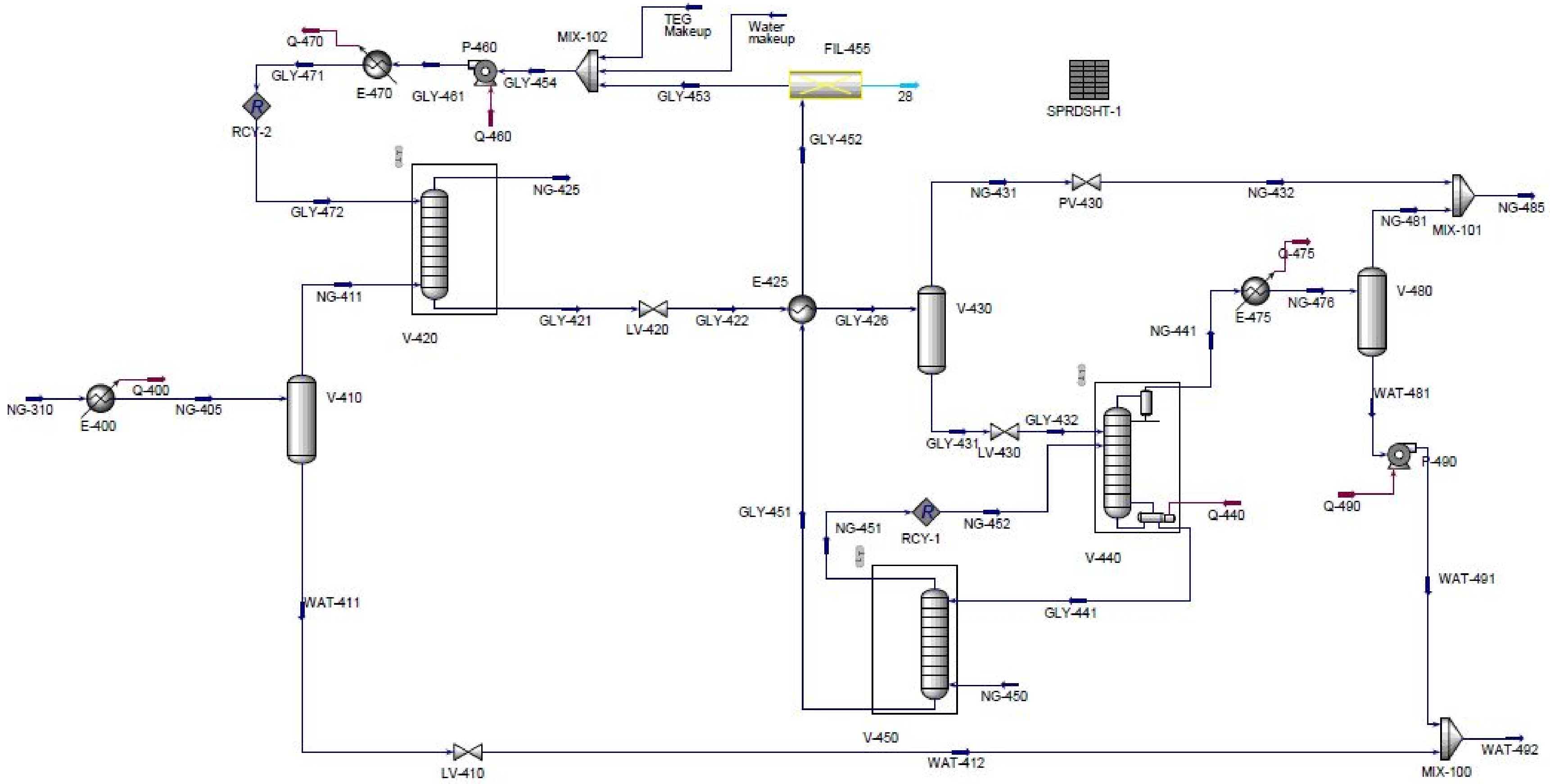

Supplementary Figure 10. Dehydration plant process model schematic.

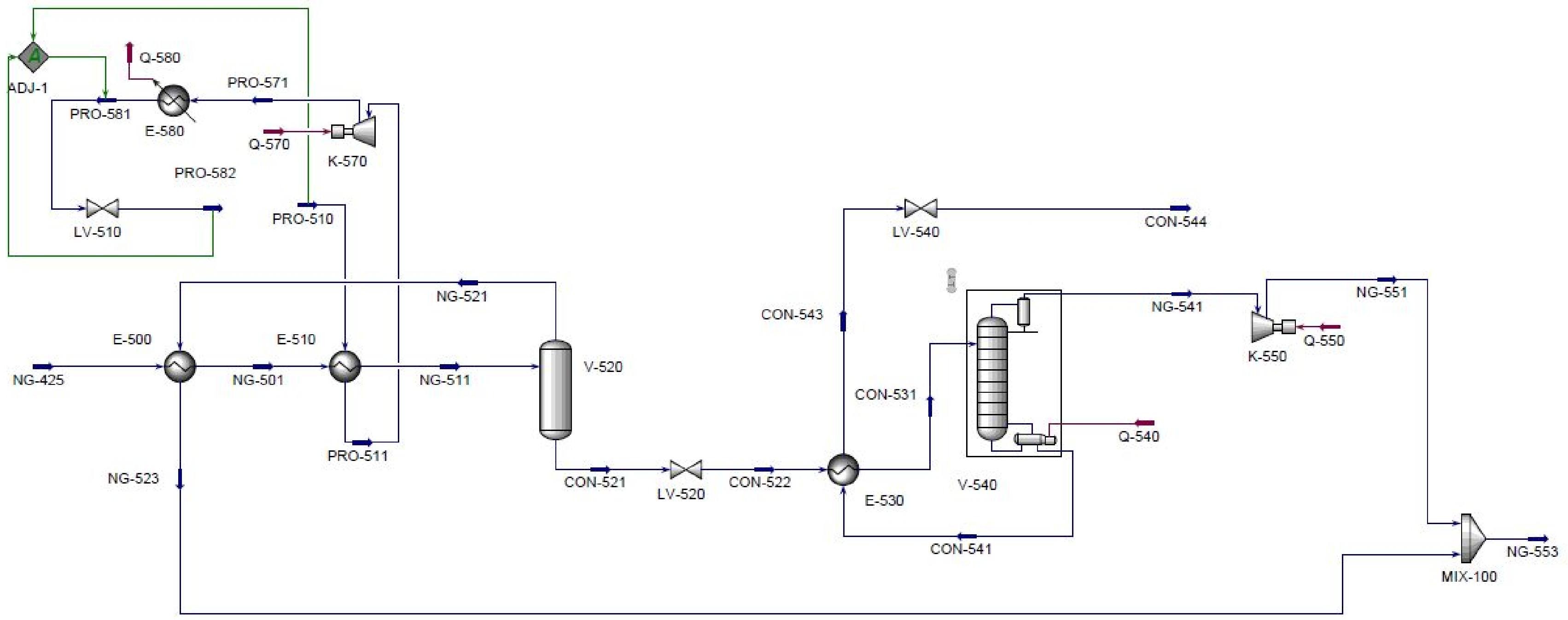

Supplementary Figure 11. Refrigeration plant process model schematic.

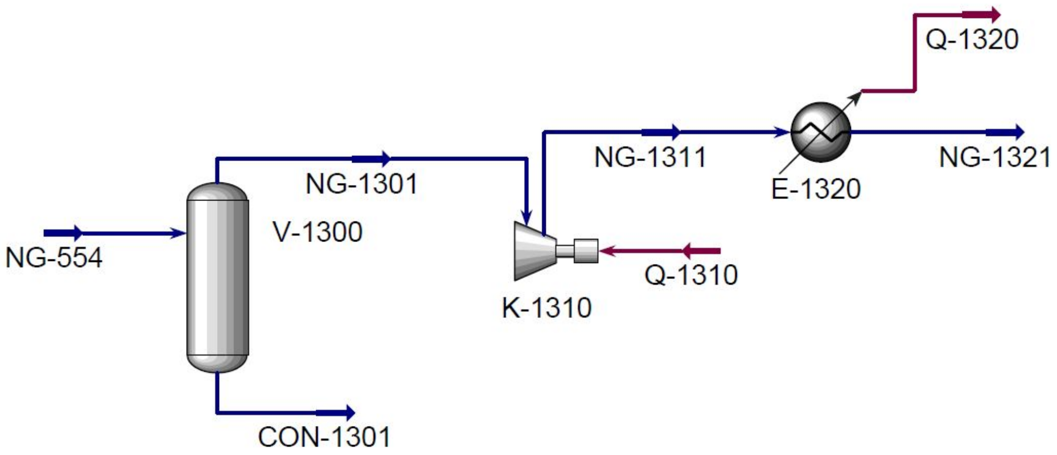

Supplementary Figure 12. Sales compressor process model schematic.

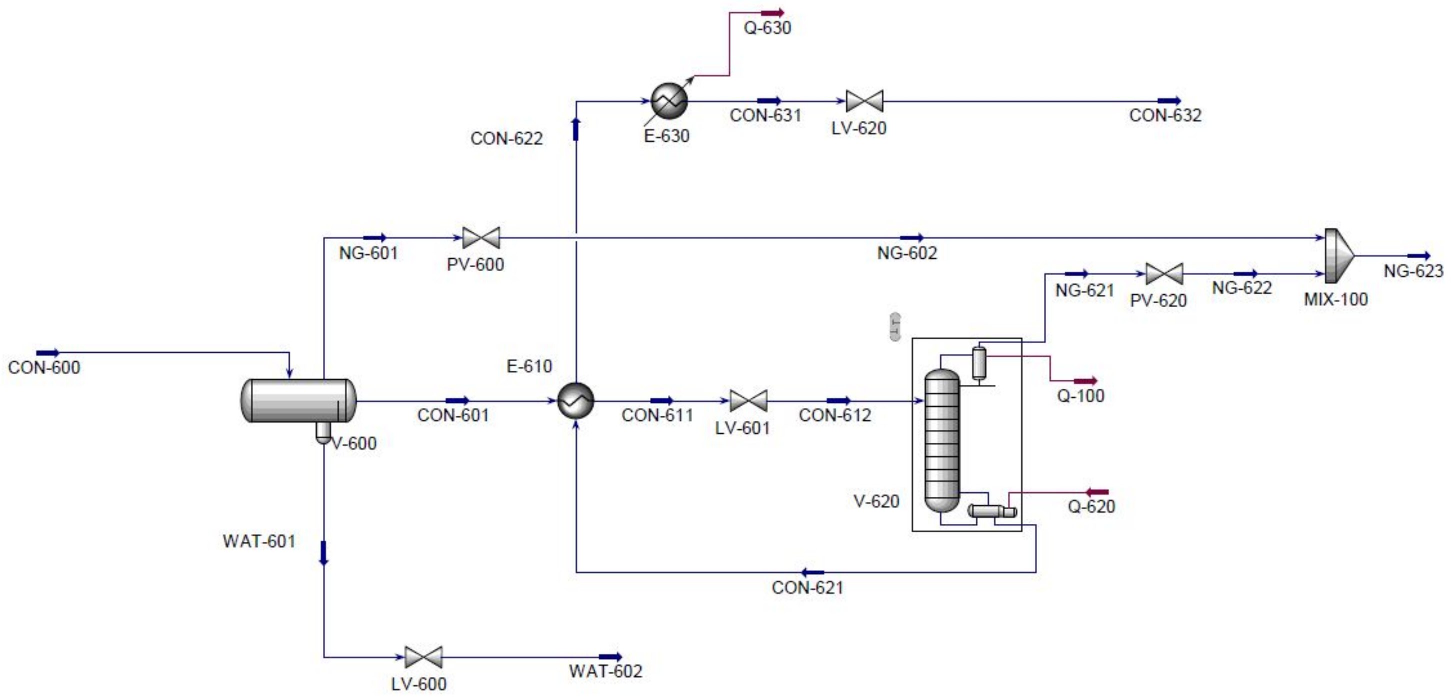

Supplementary Figure 13. Condensate stabilisation plant model schematic.

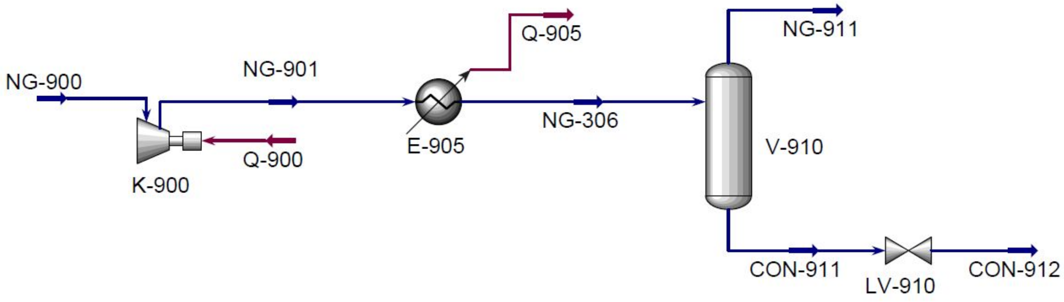

Supplementary Figure 14. Recycle compressor model schematic.

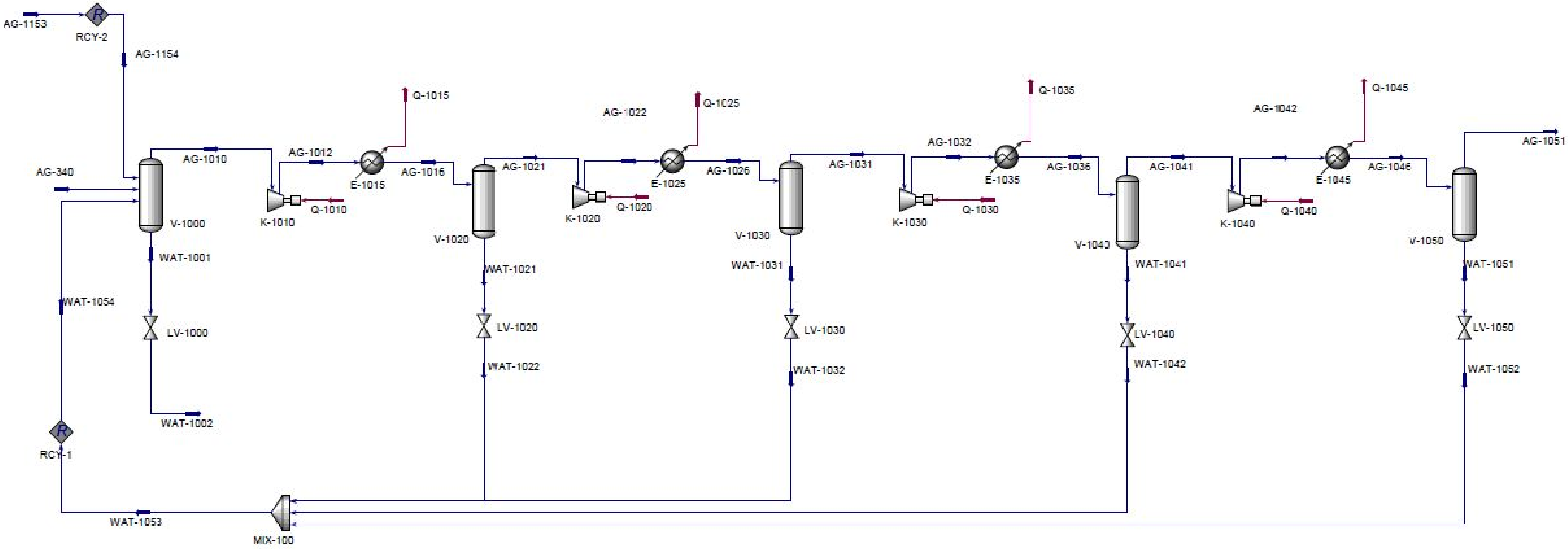

Supplementary Figure 15. Acid gas compressor model schematic (first four stages).

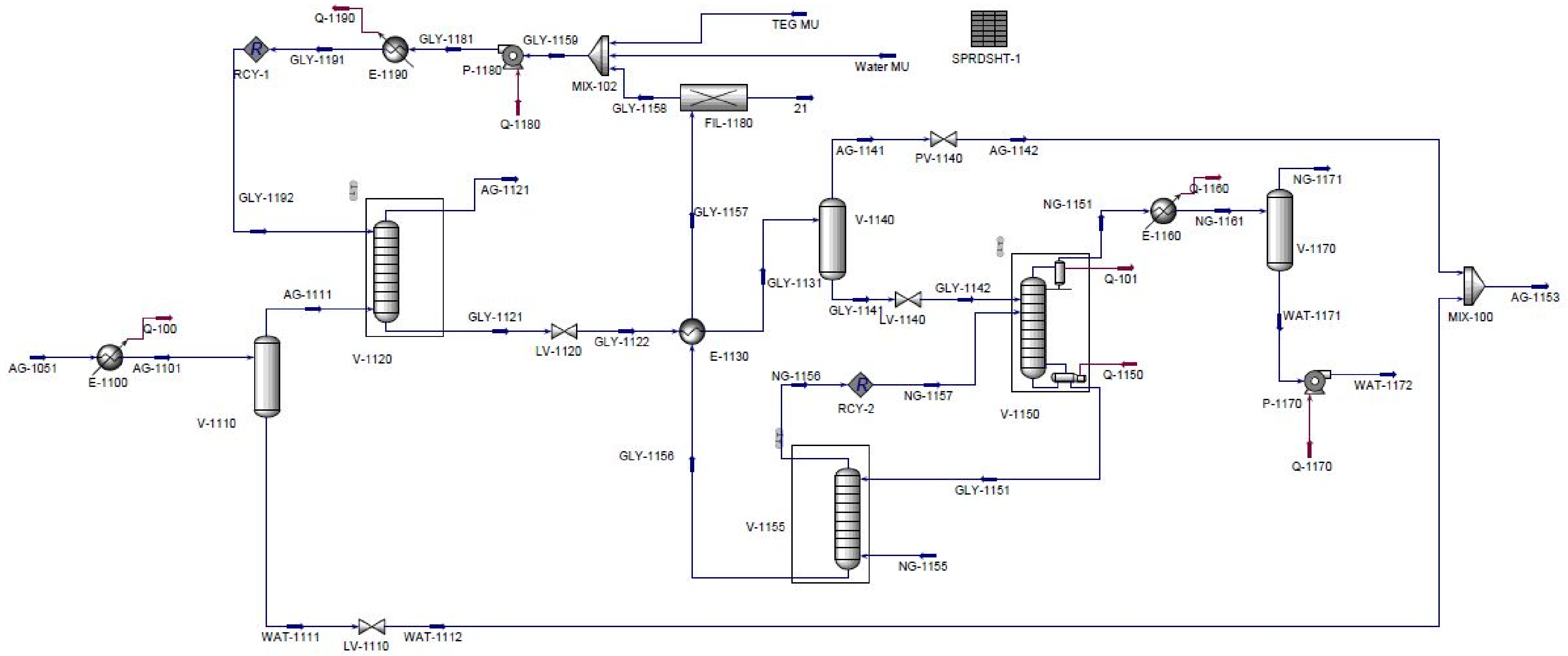

Supplementary Figure 16. Acid gas dehydration plant schematic.

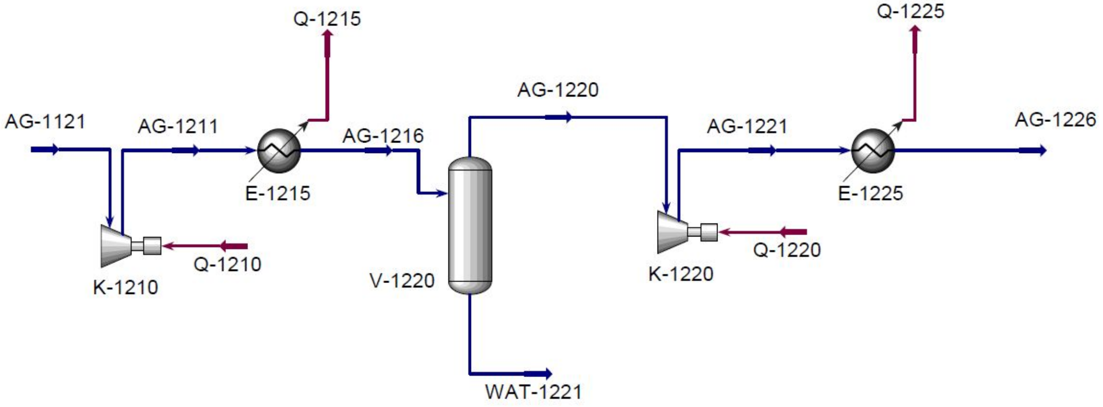

Supplementary Figure 17. Acid gas compressor model schematic (final two stages).

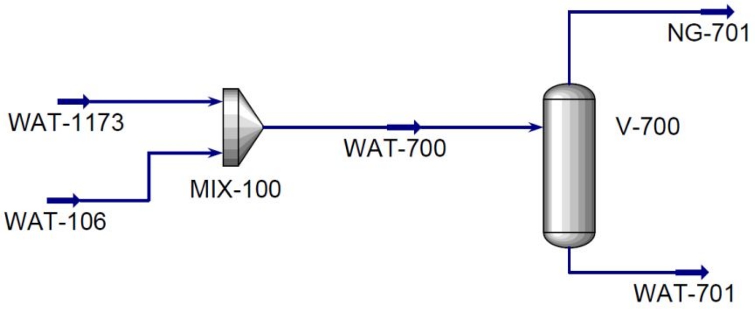

Supplementary Figure 18. Water processing model schematic.

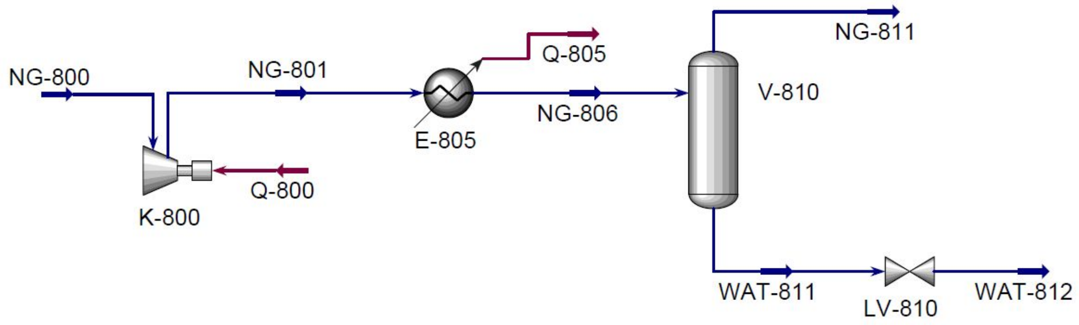

Supplementary Figure 19. Vapour recovery unit model schematic.

2.2 Process model assumptions

The NG processing plant design was based on supplying a net NG flow rate with LHV energy content of 1626 MW based on the CCGT fuel consumption.

All compressors were assumed to be reciprocating with an isentropic efficiency of 82%[49]. The small recycle compressors (K-550, K-800, K-900), propane compressor (K-570), and acid gas compressors were assumed to be electric. For the scenario assuming NG engine drive for the NG inlet and sales compressors (K-210, K-240, K-1310), engine fuel consumption was calculated based on brake specific fuel consumption of 9.50 MJ_{LHV}/bkW-hr which is representative of the type and scale of compressors typically employed in upstream NG industry in BC[13]. For electric drive compressors, a motor efficiency of 95% was assumed.

Electricity consumption for pumps was based on isentropic efficiency of 86% for centrifugal and 90% for reciprocating[49]. Compressor cooling fan electricity consumption was estimated as 3% of compressor drive power and other plant electrical loads (e.g., control system, instrument air compressor) were estimated as 2% of compressor drive power.

NG consumption for glycol/amine reboiler duty was based on a thermal efficiency of 85/90%.

Produced water inlet flow was calculated using an average water-to-NG ratio of 0.141 m^3/E3m^3 based on the estimated lifetime water (20 600 m^3) and NG production of the wells sampled in this study[13]. The plant inlet was assumed to be 1000 kPa and 20°C. The discharge pressure of the NG compressor (K-240) and sales compressors (K-1310) were 4115 kPa and 9030 kPa respectively with intercooling/aftercooling to 50°C. We combine NG, condensate, and water based on typical Montney analyses and the ratio of EUR for each commodity at the inlet separator conditions to determine the composition of the respective streams. Typical extended gas analysis (i.e., including trace concentrations of heavier hydrocarbons) was not publicly available. While this is not expected to materially impact the overall mass-energy balance for the process or results of this study, it will impact specific details of the process design (e.g., recovery of aromatic hydrocarbons from glycol regeneration overhead vapours).

Design parameters for the regenerative amine sweetening (**Supplementary Table 33**), NG dehydration (**Supplementary Table 34**), refrigeration plant (**Supplementary Table 35**), and condensate stabiliser (**Supplementary Table 36**) processes were assumed based on typical parameters used in the NG processing industry[49] and adjusted as needed to achieve outlet NG and condensate compositions consistent with typical sales specifications[83,84]. Tray efficiencies for the amine absorber (14-23% for CO_2 and 48-51% for H_2S) and regenerator (31-43% for CO_2 and 58-61% for H_2S) were calculated by Aspen Hysys based on assumed tower characteristics.

Supplementary Table 33. Amine sweetening design parameters used in this study.

	**Value**
Absorbent	MEA 30%wt
Rich amine loading (mol CO_2/mol amine)	0.47
Absorber – number of stages	20
Absorber gas inlet temperature (°C)	50
Absorber amine inlet temperature (°C)	50
Water wash gas outlet pressure (kPa)	4050
Lean-rich exchanger pinch (°C)	16
Regenerator – number of stages	20
Reflux condenser temperature (°C)	50
Reboiler temperature (°C)	121
Regenerator reflux condenser outlet pressure (kPa)	160
Amine charge pump isentropic efficiency	86%[49]
Amine booster pump isentropic efficiency	90%[49]

Supplementary Table 34. Dehydration plant design parameters used in this study.

	Value
Absorbent	TEG
Circulation rate (l_{TEG}/kg_{H2O})	30
Absorber – number of theoretical stages	5
Absorber gas inlet temperature (°C)	40
Absorber glycol inlet temperature (°C)	45
Absorber gas outlet pressure (kPa)	3990
Regenerator – number of theoretical stages	10
Reboiler temperature (°C)	198
Lean glycol concentration (%wt.)	99.7
Glycol inlet temperature (°C)	60
Regenerator outlet pressure (kPa)	120
Glycol pump isentropic efficiency	90%[49]

Supplementary Table 35. Refrigeration plant design parameters used in this study.

	Value
Low temperature separator temperature (°C)	-15
NG outlet pressure (kPa)	3900
Stabiliser – number of theoretical stages	10
Reboiler temperature (°C)	122
Stabiliser condensate inlet temperature (°C)	10
Stabiliser vapour outlet pressure (kPa)	900

Supplementary Table 36. Condensate stabiliser design parameters used in this study.

	Value
Stabiliser – number of theoretical stages	10
Reboiler temperature (°C)	113
Stabiliser condensate inlet temperature (°C)	50
Stabiliser vapour outlet pressure (kPa)	350

Parameters for the acid gas dehydration plant (**Supplementary Table 37**) were selected to achieve less than 64 mg_{H2O}/m^3 acid gas. The final discharge pressure from the last acid gas compressor stage was assumed to be 15.3 MPa.

Supplementary Table 37. Acid gas dehydration plant design parameters used in this study.

	Value
Absorbent	TEG
Circulation rate (l_{TEG}/kg_{H2O})	30
Absorber – number of theoretical stages	5
Absorber gas inlet temperature (°C)	40
Absorber glycol inlet temperature (°C)	50
Absorber gas outlet pressure (kPa)	3700
Regenerator – number of theoretical stages	10
Reboiler temperature (°C)	197
Lean glycol concentration (%wt)	99.5
Glycol inlet temperature (°C)	60
Regenerator reflux condenser outlet pressure (kPa)	120
Glycol pump isentropic efficiency	90%[49]

2.3 Process model results

The shaft power requirements for compressors and pumps (**Supplementary Table 38**) and process heat requirements (**Supplementary Table 39**) from the NG processing plant model were used to determine the fuel and electricity consumption required for each of the design cases (**Supplementary Table 40**) and the overall plant flows (**Supplementary Table 41**). The processing plant model results were used to determine the amount of well effluent and electricity input that is required per unit of energy consumed by the CCGT (**Supplementary Table 42**).

Supplementary Table 38. Shaft power calculated in the NG processing plant model. All values are kW for the design cases with NG engine driven inlet compressors and electric motor driven inlet compressors.

Equipment	NG-drive inlet compressor	Electric-drive inlet compressor
Compressors:		
Inlet (K-210/240)	8144	8037
Sales (K-1310)	4476	4476
Propane (K-570)	1440	1422
Refrigeration overhead (K-550)	282	279
Recycle (K-900)	31	31
VRU (K-800)	16	16
Acid gas (K-1010/1020/1030/1040 & 1210/1220	103	102
Pumps (total)	29	29

Supplementary Table 39. Process heat requirements calculated in the NG processing plant model. All values are MJ/h for the design cases with NG engine driven inlet compressors and electric motor driven inlet compressors.

Equipment	NG-drive inlet compressor	Electric-drive inlet compressor
Amine regenerator (V-330)	4162	4163
Glycol regenerator (V-440)	3369	3369
Refrigeration stabiliser (V-540)	3721	3673
Condensate stabiliser (V-620)	1293	1275
Acid gas glycol regenerator (V-1150)	18	18

Supplementary Table 40. Electricity and NG consumption estimates from the NG processing plant model. Values are provided for the design cases with NG engine driven inlet compressors and electric motor driven inlet compressors.

	NG-drive inlet compressor	Electric-drive inlet compressor
Electricity consumption (kW):		
Compressors	1972	15 119
Cooling fans	435	431
Pumps	29	29
Other	290	287
**Total electricity**	**2726**	**15 866**
NG consumption (GJ/h):		
Compressors (K-210/240/1310)	119.9	0
Process heat	14.18	14.11
Dehydrator stripping gas	9.38	9.38
**Total process NG**	**143.5**	**23.5**

Supplementary Table 41. Overall flows from the NG processing plant model. Values are provided in energy content (GJ/h, LHV basis) for the design cases with NG engine driven inlet compressors and electric motor driven inlet compressors.

	NG-drive inlet compressor	Electric-drive inlet compressor
Process inlet:		
NG	6449	6364
Condensate	145.2	143.3
**Total well effluent**	**6594**	**6507**
Products:		
NG	5811	5853
Condensate	642	634
**Total products**	**6454**	**6487**

Supplementary Table 42. Required well effluent, fuel, and electricity inputs to NG processing plant. Values expressed per unit of energy produced by the NG gas processing plant LHV basis (GJ_{out}) for the design cases with NG engine driven inlet compressors and electric motor driven inlet compressors. Total energy production includes liquid condensate co-product allocation.

	NG-drive inlet compressor	Electric-drive inlet compressor
Well effluent input (GJ_{In}/ GJ_{out})	1.0217	1.00308
Electricity input (kWh/ GJ_{out})	0.422	2.446
Reciprocating engine NG consumption (GJ/ GJ_{out})	1.86E-2	0
Boiler NG consumption (GJ/ GJ_{out})	2.20E-3	2.17E-3

2.4 NG processing plant cost model

Capital and operating/maintenance costs for the NG processing facility were estimated using Aspen Process Economic Analyzer[70]. Costs were estimated separately for each of the design cases (NG drive inlet compressor and electric drive inlet compressor) based on the process design described above (**Supplementary Table 43**). In addition to the process unit operations modelled in the process simulations, the following additional equipment were included to obtain a more representative cost estimate: high pressure emergency flare stack (610 mm x 40 m), low pressure emergency flare stack (150 mm x 40 m), high pressure flare knockout drum (1.7 m x 8 m), low pressure flare knockout drum (1 m x 4 m), fuel gas scrubber (1 m x 3 m), sales NG scrubber (2 m x 4 m), water storage tanks (4 x 160 m^3), recycle tank (160 m^3), condensate storage tanks (4 x 160 m^3), water pumps (centrifugal charge and reciprocating main), condensate pumps (centrifugal

charge and reciprocating main), recycle pumps, water filters, condensate filters, and instrument air system. Installed backup spares (100%) were assumed for all main process pumps and the propane compressor. The inlet separator was assumed to be oversized (4 m x 25 m) to accommodate inlet slugs from the gathering pipeline during pigging operations. The Q1 2018 base cost estimates obtained from Aspen Process Economic Analyzer for North America construction were adjusted to 2017 base year using the Chemical Engineering Plant Cost Index[55].

Supplementary Table 43. Capital and maintenance cost estimates for NG processing plant assumed in this study for each design case. Amounts in US$ for North America with base year of 2018.

	**NG-drive inlet compressor**	**Electric-drive inlet compressor**
Capital	$106 110 000	$102 230 000
Maintenance	$983 000/year	$747 000/year

3 SUPPLEMENTARY NOTE 3 – ADDITIONAL RESULTS

3.1 NG supply chain impacts

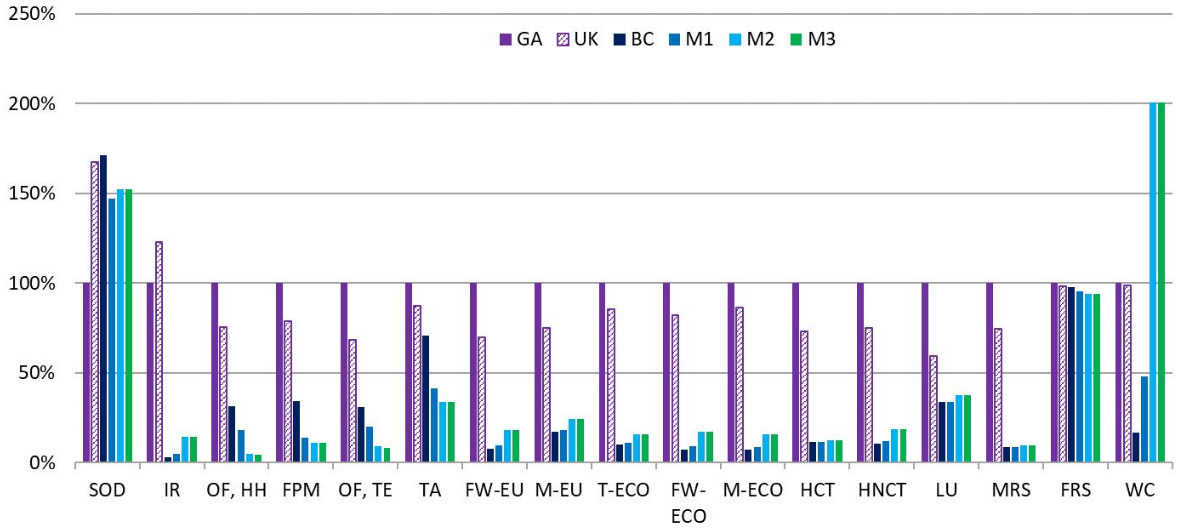

Supplementary Figure 20. Life cycle environmental impacts of NG production practices. Comparison of life cycle environmental impact intensities for global average NG supply (GA), UK average NG supply (UK), BC average upstream production (BC), and the three low-emission cases developed in this study for BC Montney production with NG drive compressors (M1), electric drive compressors (M2), and electric drive compressors with reduced fugitive emissions (M3). Environmental impact categories: stratospheric ozone depletion (SOD), ionizing radiation (IR), ozone formation – human health (OF-HH), fine particulate matter (FPM), ozone formation – terrestrial ecosystems (OF-TE), terrestrial acidification (TA), freshwater eutrophication (FW-EU), marine eutrophication (M-EU), terrestrial ecotoxicity (T-ECO), freshwater ecotoxicity (FW-ECO), marine ecotoxicity (M-ECO), human carcinogenic toxicity (HCT), human non-carcinogenic toxicity (HNCT), land use (LU), mineral resource scarcity (MRS), fossil resource scarcity (FRS), and water consumption (WC). Values shown as a percentage of impact intensity for global average NG supply per unit energy on LHV basis.

3.2 Baseload CCGT scenarios

Environmental impact results for CCGT with CCS for each NG supply scenario are presented and compared with wind and photovoltaic generation in **Supplementary Figure 21** and **Supplementary Figure 22**. This study focused on assessing results for CCGT with CO_2 capture rates greater than 95% based on the findings of several prior studies that high capture rates for CCGT are feasible and can be achieved at low marginal cost (e.g., refs.[63,64,85,86]). However, we also

calculated global warming (GW) intensity results for the commonly assumed historical CO_2 capture rate of 90% based on the lowest capture rate in the NETL baseline study[63] to facilitate comparisons of the results of this study with prior work. The effect of duty cycles on lifecycle environmental impact intensities based on the five existing CCGT facilities considered in this study is shown in **Supplementary Figure 23**.

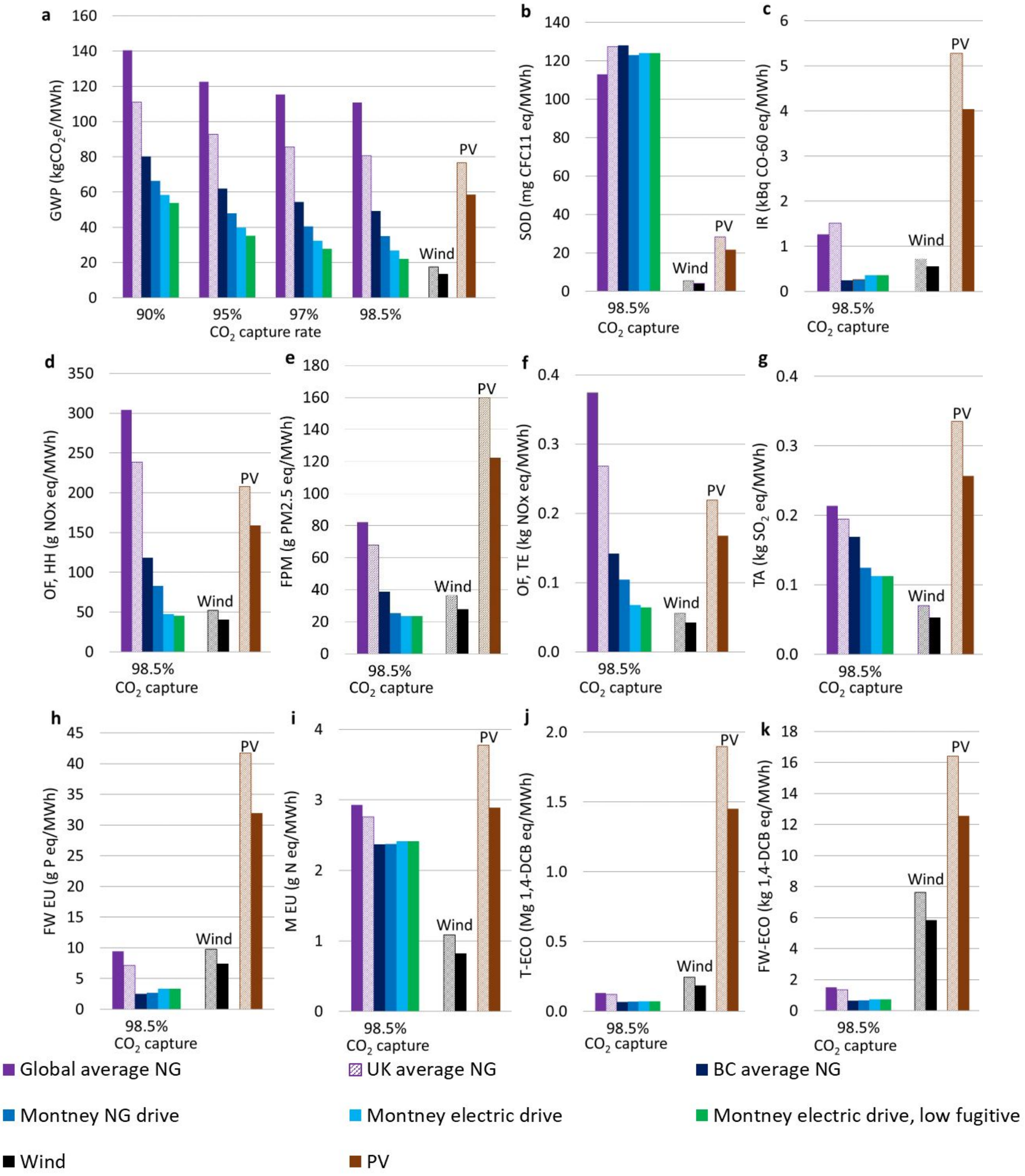

Supplementary Figure 21. Midpoint environmental impact indicators per MWh electricity produced for CCGT with CCS and six NG supply chain scenarios compared to wind and photovoltaic generation. NG supply scenarios: global average supply (Ecoinvent), UK average supply (Ecoinvent), BC average production (2020), BC Montney production with NG drive compressors, BC Montney production with electric drive compressors, and BC Montney production with electric drive compressors and 2030 fugitive methane emission reduction target achieved. Results for wind and photovoltaic shown for BC (diagonal hatch) and western USA (solid). (a) Global warming potential. (b) Stratospheric ozone depletion. (c) Ionizing radiation. (d) Ozone formation – human health. (e) Fine particulate matter. (f) Ozone formation – terrestrial ecosystems. (g) Terrestrial acidification. (h) Freshwater eutrophication. (i) Marine eutrophication. (j) Terrestrial ecotoxicity. (k) Freshwater ecotoxicity.

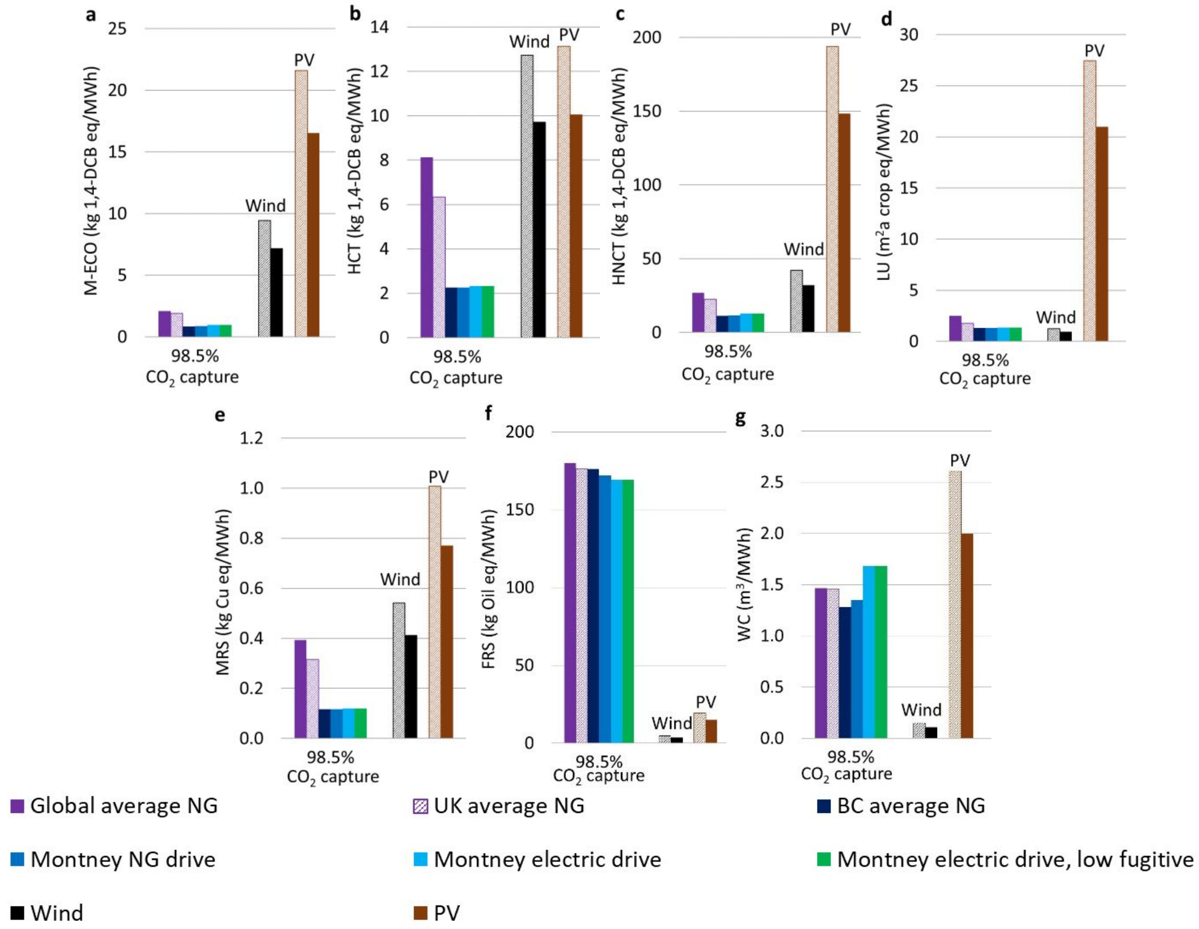

Supplementary Figure 22. Midpoint environmental impact indicators per MWh electricity produced for CCGT with CCS and six NG supply chain scenarios compared to wind and photovoltaic generation. NG supply scenarios: global average supply (Ecoinvent), UK average supply (Ecoinvent), BC average production (2020), BC Montney production with NG drive compressors, BC Montney production with electric drive compressors, and BC Montney production with electric drive compressors and 2030 fugitive methane emission reduction target achieved. Results for wind and photovoltaic shown for BC (diagonal hatch) and western USA (solid). (a) Marine ecotoxicity. (b) Human carcinogenic toxicity. (c) Human non-carcinogenic toxicity. (d) Land use. (e) Mineral resource scarcity. (f) Fossil resource scarcity. (g) Water consumption.

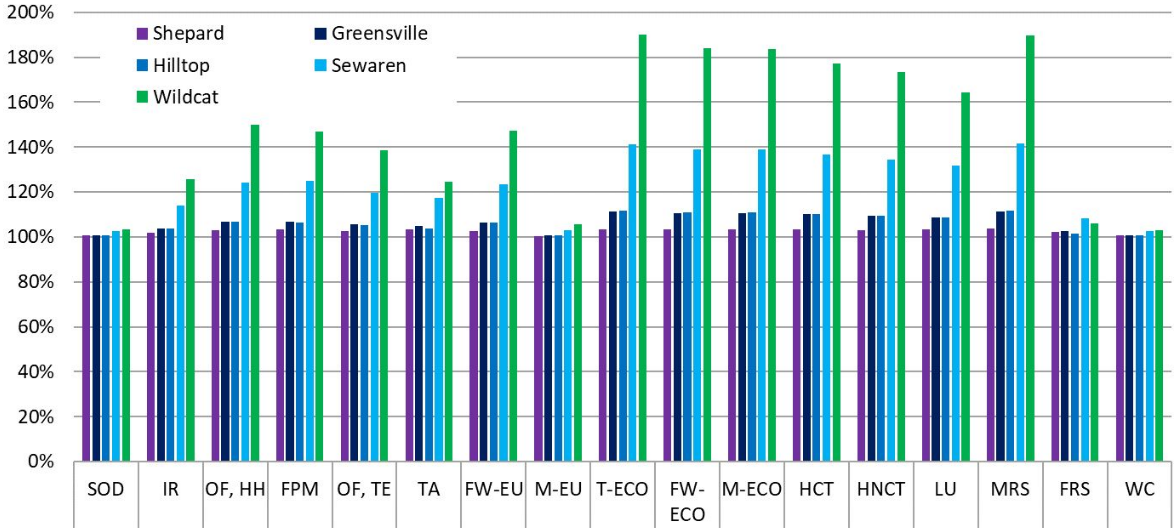

Supplementary Figure 23. Effect of CCGT duty cycle on life cycle environmental impact intensities. Comparison of midpoint life cycle environmental impact intensities for electricity generated from CCGT with CCS operating based on average duty cycles for five existing CCGT facilities in North America. Values shown as a percentage of the impact intensities (per MWh of net electricity generated) for the NETL baseload scenario (capacity factor 0.85) for CCGT with 97% CO_2 capture. BC Montney NG supply with electric drive compression and reduced fugitive methane emissions. Environmental impact categories: stratospheric ozone depletion (SOD), ionizing radiation (IR), ozone formation – human health (OF-HH), fine particulate matter (FPM), ozone formation – terrestrial ecosystems (OF-TE), terrestrial acidification (TA), freshwater eutrophication (FW-EU), marine eutrophication (M-EU), terrestrial ecotoxicity (T-ECO), freshwater ecotoxicity (FW-ECO), marine ecotoxicity (M-ECO), human carcinogenic toxicity (HCT), human non-carcinogenic toxicity (HNCT), land use (LU), mineral resource scarcity (MRS), fossil resource scarcity (FRS), and water consumption (WC).

Environmental impact intensities for electricity generated by CCGT with CCS are negatively correlated with nominal operating hours per year (**Supplementary Figure 24** and **Supplementary Figure 25**). The strength of the negative correlation varies depending on the relative contributions of fixed infrastructure and startup emissions to the environmental impact category. Environmental impact intensities are more strongly corelated with nominal operating hours than startup type within the range of hot/warm/cold startup distributions (40/40/20%, 60/30/10%, and 80/15/5%) that were considered in this analysis. Rich solvent storage significantly reduces GW intensity for scenarios with low nominal operating hours (more frequent start/stop cycles) but does not materially affect any other environmental impact category.

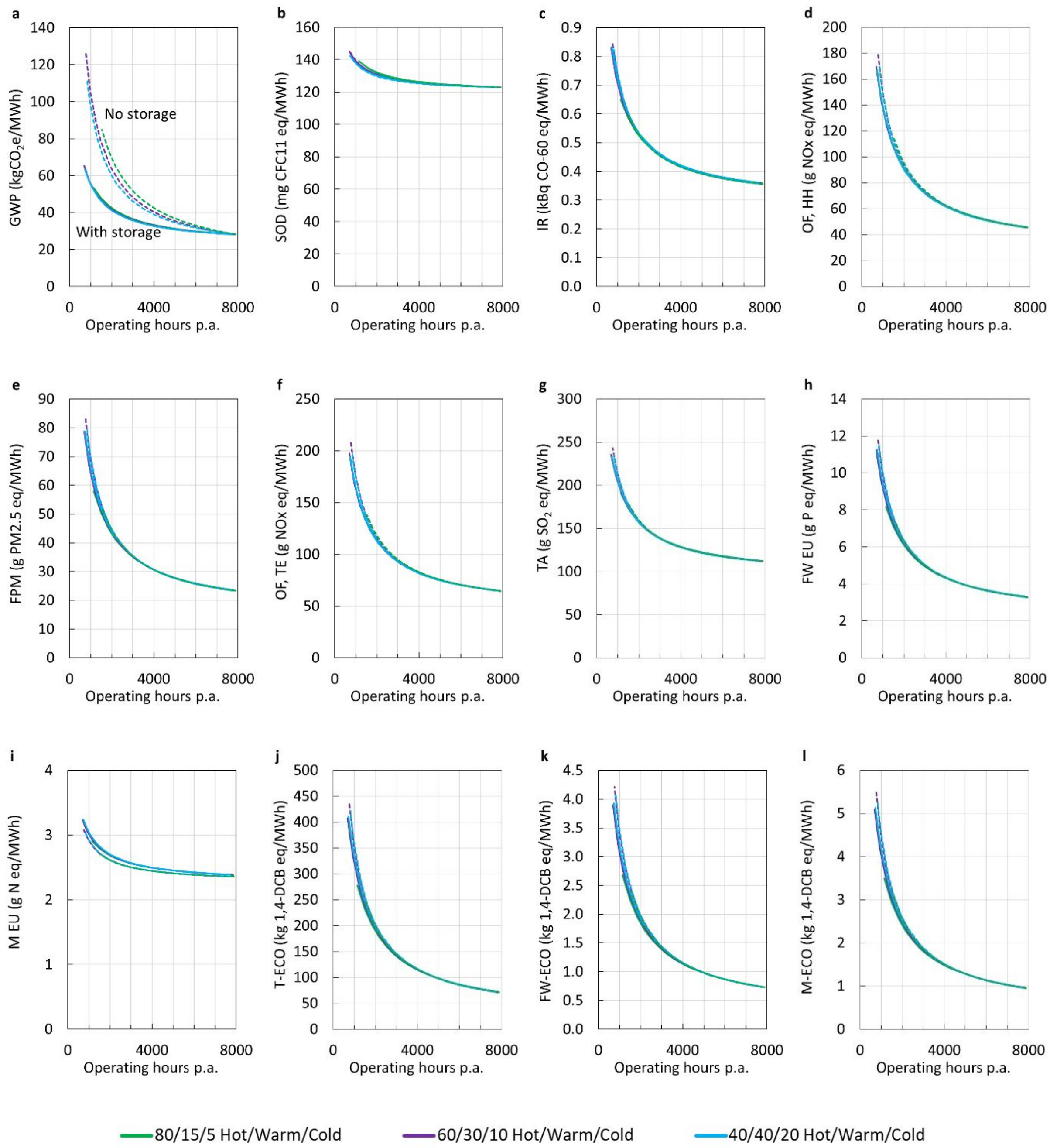

Supplementary Figure 24. Effect of operating hours on life cycle environmental impact intensities. Environmental impacts per MWh electricity produced from CCGT with CCS versus nominal operating hours per year for three different distributions of hot/warm/cold starts. Baseline case (dashed) compared to case with rich solvent storage to mitigate startup CO_2 emissions (solid). CCGT operating at 95% rated output during normal operation. **(a)** Global warming potential. **(b)** Stratospheric ozone depletion. **(c)** Ionizing radiation. **(d)** Ozone formation – human health. **(e)** Fine particulate matter. **(f)** Ozone formation – terrestrial ecosystems. **(g)** Terrestrial acidification. **(h)** Freshwater eutrophication. **(i)** Marine eutrophication. **(j)** Terrestrial ecotoxicity. **(k)** Freshwater ecotoxicity. **(l)** Marine ecotoxicity.

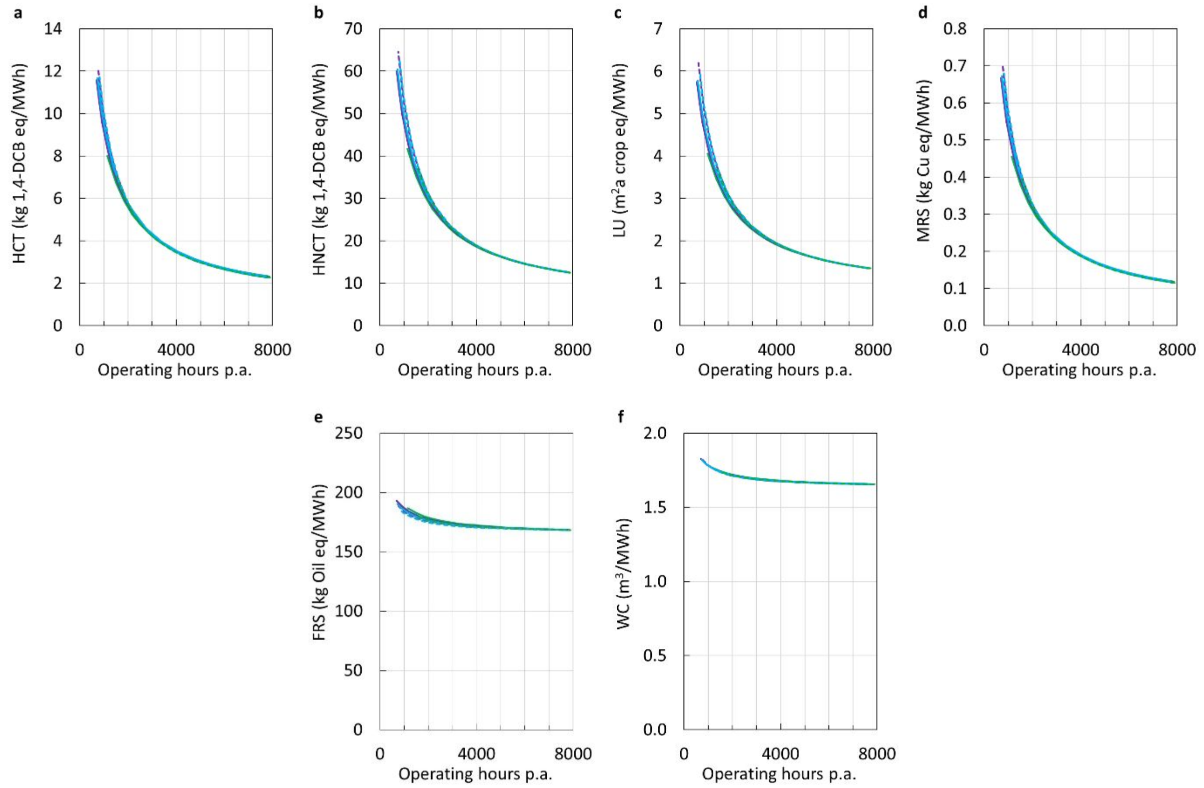

Supplementary Figure 25. Effect of operating hours on life cycle environmental impact intensities. Environmental impacts per MWh electricity produced from CCGT with CCS versus nominal operating hours per year for three different distributions of hot/warm/cold starts. Baseline case (dashed) compared to case with rich solvent storage to mitigate startup CO_2 emissions (solid). CCGT operating at 95% rated output during normal operation. (a) Human carcinogenic toxicity. (b) Human non-carcinogenic toxicity. (c) Land use. (d) Mineral resource scarcity. (e) Fossil resource scarcity. (f) Water consumption.

The effect of startup/shutdown cycles on life cycle GHG emissions and average GHG emission intensity with average UK NG supply are shown in **Supplementary Figure 26** and **Supplementary Figure 27**. These can be compared against Figures 6 and 7 in the main text for the low-emission NG supply scenario with electric compressors and low fugitive emissions.

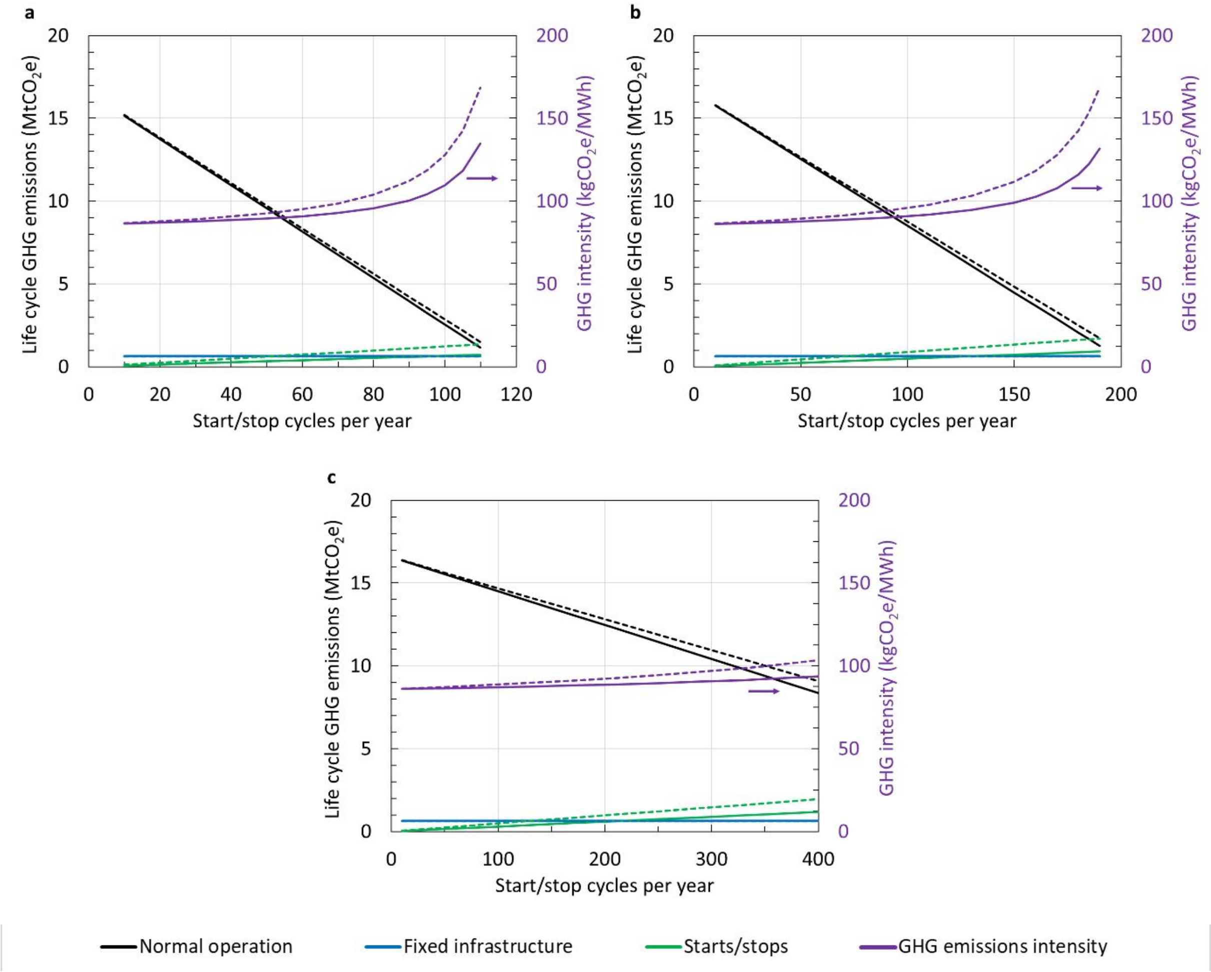

Supplementary Figure 26. Impact of start/stop cycles on life cycle GHG emissions (UK NG supply). Effect of start/stop frequency on the contribution of normal operation, fixed infrastructure, and start/stop cycles to life cycle GHG emissions for CCGT with CCS (LHS) and overall GHG emission intensity (RHS) of electricity produced. Baseline case (dashed) compared to case with rich solvent storage to mitigate startup CO_2 emissions (solid). CCGT operating at 95% rated output during normal operation with 97% CO_2 capture rate. UK average NG supply based on Ecoinvent background inventory. **(a)** cold starts, **(b)** warm starts, and **(c)** hot starts. Duration of cooldown preceding each hot/warm/cold start is assumed to be 8/36/64 hours.

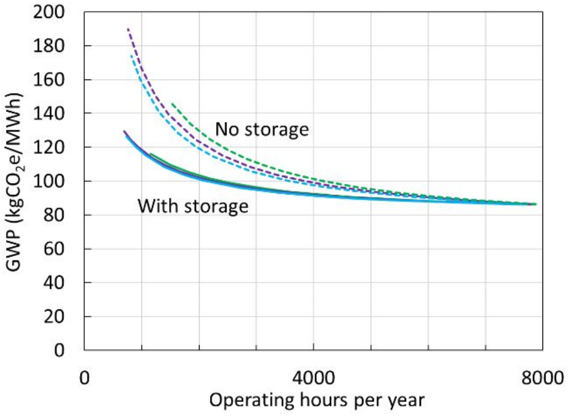

Supplementary Figure 27. Effect of annual operating hours on life cycle GHG emission intensity (UK NG supply). Life cycle GHG emission intensity per MWh electricity produced from CCGT with CCS versus nominal operating hours per year for three different distributions of hot/warm/cold starts: 40/40/20% (blue), 60/30/10% (purple), and 80/15/5% (green). Baseline case (dashed) compared to case with rich solvent storage to mitigate startup CO_2 emissions (solid). CCGT operating at 95% rated output during normal operation with 97% CO_2 capture rate. UK average NG supply based on Ecoinvent background inventory.

3.3 Sensitivity analysis

All individual parameters affect life cycle GW intensity less than 7% over the ranges considered for the lowest emission scenario in this study (Supplementary Figure 28). Life cycle GW intensity for CCGT with CCS is most sensitive to amine consumption in the CO_2 capture plant (±6.5% for ±60% change) due to relatively high sensitivity and low confidence in the estimate due to limited and highly variable public data[66,67]. This uncertainty is notable because this study used monoethanolamine as a proxy for the proprietary solvent assumed in the US NETL study due to lack of public data on consumption rates for the proprietary solvent. CCGT fuel consumption, which impacts both direct and indirect emissions, is also a key uncertainty in the GW intensity results (±5.8% for ±10% change). Despite assessing sensitivity using the low-fugitive NG supply scenario, fugitive emission factor is the third most sensitive parameter for life cycle GW intensity (±4.7% for ±30% change). The relatively high sensitivity of GW intensity to the CCGT nitrous oxide emission factor is notable because of the low confidence expressed in published nitrous oxide emission factors for gas turbines[30,54]. The total share of baseload life cycle GHG emissions associated with EEIO characterisation factors from all sources included in this study is 3.9% in the scenario with 98.5% fossil-CO_2 capture and the lowest emission NG supply chain.

This study assumes that the CO_2-rich solvent stored during startup is regenerated using spare capacity at a relatively low gas turbine output power (50%) to minimise the time required to regenerate the solvent. Many other assumptions are possible depending on operating needs/preferences (e.g., other part-load gas turbine output or the regeneration equipment could be oversized to regenerate stored solvent with 100% gas turbine output). In practice, plant operators would have flexibility regenerating stored solvent to maximise profitability for each start/shutdown sequence depending on electricity/NG prices and operational considerations such as reserve requirements. This choice has little effect on the overall LCA results. For example, the difference in total electricity generated and NG consumed is less than 1% between a cold start where CO_2-rich amine is regenerated at 80% gas turbine output and a cold start where amine is regenerated at 50% and then the CCGT is operated at rated output for the difference in start durations (6.5 h total cold start duration at 80% v. 3.5 h at 50%).

Variation in CCGT amine consumption (±60%) significantly affects life cycle impact intensity for ionizing radiation (±20%), freshwater eutrophication (±11%), and marine eutrophication (±52%); while variation in CCGT nitrous oxide emissions (±30%) significantly affects stratospheric ozone depletion (±22%). The only other sensitivity analysis parameters which affect any life cycle impact intensity by more than 10% are process electrical demand (±10% on ionizing radiation) and CCGT construction (±11-12% on terrestrial ecotoxicity, freshwater ecotoxicity, marine ecotoxicity, and mineral resource scarcity).

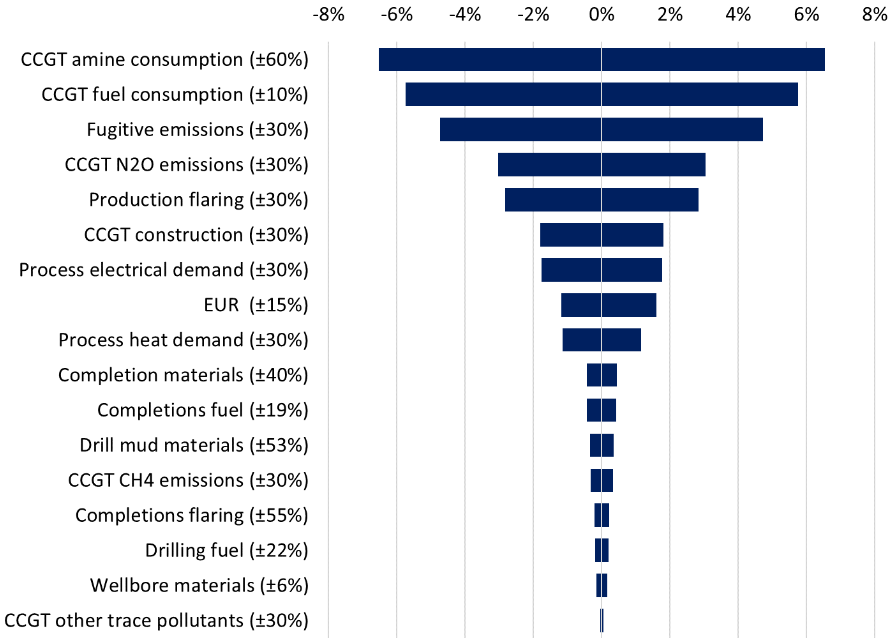

Supplementary Figure 28. Sensitivity analysis of life cycle GW intensity. Percent change in life cycle GHG emission intensity per MWh electricity produced from CCGT with CCS for the baseload scenario (98.5% CO_2 capture) based on changes to indicated parameters. NG supply scenario is BC Montney production with electric drive compressors and fugitive emissions reduced 75% from 2014. Rationale for sensitivity ranges for each parameter provided in Supplementary Note 1.8.1.

There is significant uncertainty in unburned methane emission rates during CCGT startup due to very limited public data (Supplementary Note 1.6.3); however, direct emissions of unburned methane contribute relatively little to life cycle GHG emissions in this study (e.g., 2.4% for the Wildcat duty cycle with 97% CO_2 capture, interim solvent storage, and the lowest emission NG supply scenario in this study).

4 REFERENCES

(1) Forster, P.; Storelvmo, T.; Armour, K.; Collins, W.; Dufresne, J. L.; Frame, D.; Lunt, D. J.; Mauritsen, T.; Palmer, M. D.; Watanabe, M.; et al. *The Earth's Energy Budget, Climate Feedbacks, and Climate Sensitivity. In: Climate Change 2021: The Physical Science Basis. Contribution of Working Group to the Sixth Assessment Report of the Intergovernmental Panel on Climate Change [Masson-Delmotte, V., P. Zhai, A. Pirani, S. L. Connors, C. Péan, S. Berger, N. Caud, Y. Chen, L. Goldfarb, M. I. Gomis, M. Huang, K.Leitzell, E. Lonnoy, J.B.R. Matthews, T. K. Maycock, T. Waterfield, O. Yelekçi, R. Yu and B. Zhou (eds.)]. Cambridge University Press. In Press.*; 2021.

(2) Myhre, G.; Shindell, D.; Bréon, F.-M.; Collins, W.; Fuglestvedt, J.; Huang, J.; Koch, D.; Lamarque, J.-F.; Lee, D.; Mendoza, B.; et al. Anthropogenic and Natural Radiative Forcing. In: Climate Change 2013: The Physical Science Basis. Contribution of Working Group I to the Fifth Assessment Report of the Intergovernmental Panel on Climate Change [Stocker, T.F., D. Qin, G.-K. Plattner, M. Tignor, S.K. Allen, J. Boschung, A. Nauels, Y. Xia, V. Bex and P.M. Midgley (eds.)]. Cambridge University Press, Cambridge, United Kingdom and New York, NY, USA. **2013**.

(3) McDaniel. *British Columbia Montney Supply Study and Type Curve Analysis. McDaniel & Associates Consultants Ltd. Available: https://www2.gov.bc.ca/assets/gov/farming-natural-resources-and-industry/natural-gas-oil/petroleum-geoscience/oil-gas-reports/20191118_-_resource_cost_of_supply_study_for_publication.pdf (Accessed 19 March 2023)*; 2019.

(4) *Google Earth Pro (Version 7.3.6.9345 (64-bit)) [Computer program]. Northeast British Columbia, 56.5°N, -121.75°E, Viewed 13 April 2023*; 2022.

(5) GeoBC. Landcover classification of British Columbia. Available: https://www2.gov.bc.ca/assets/gov/data/geobc/bc_land_cover.pdf (Accessed 6 April 2023). 2015.

(6) Kurz, W. A.; Shaw, C. H.; Boisvenue, C.; Stinson, G.; Metsaranta, J.; Leckie, D.; Dyk, A.; Smyth, C.; Neilson, E. T. Carbon in Canada's boreal forest — A synthesis. *Environmental Reviews* **2013**, *21* (4), 260-292. DOI: 10.1139/er-2013-0041.

(7) ECCC. *National Inventory Report 1990–2020: Greenhouse Gas Sources and Sinks in Canada. Part 2. Environment and Climate Change Canada. Available: https://publications.gc.ca/site/eng/9.506002/publication.html (Accessed 4 April 2023)*; 2022.

(8) IPCC. *2019 Refinement to the 2006 IPCC Guidelines for National Greenhouse Gas Inventories, Calvo Buendia, E., Tanabe, K., Kranjc, A., Baasansuren, J., Fukuda, M., Ngarize, S., Osako, A., Pyrozhenko, Y., Shermanau, P. and Federici, S. (eds). Published: IPCC, Switzerland. Available: https://www.ipcc-nggip.iges.or.jp/public/2019rf/index.html (Accessed 6 April 2023)*; 2019.

(9) Yeh, S.; Jordaan, S. M.; Brandt, A. R.; Turetsky, M. R.; Spatari, S.; Keith, D. W. Land Use Greenhouse Gas Emissions from Conventional Oil Production and Oil Sands. *Environmental Science & Technology* **2010**, *44* (22), 8766-8772. DOI: 10.1021/es1013278.

(10) BC Government. 2020 Major Timber Processing Facilities in British Columbia. BC Ministry of Forests. Available: https://www2.gov.bc.ca/gov/content/industry/forestry/competitive-forest-industry/forest-industry-economics/fibre-mill-information/major-timber-processing-facilities-survey (Accessed 7 April 2023). **2023**.

(11) Mykleby, P. M.; Snyder, P. K.; Twine, T. E. Quantifying the trade-off between carbon sequestration and albedo in midlatitude and high-latitude North American forests. *Geophysical Research Letters* **2017**, *44* (5), 2493-2501. DOI: 10.1002/2016gl071459.
(12) Favero, A.; Sohngen, B.; Huang, Y.; Jin, Y. Global cost estimates of forest climate mitigation with albedo: a new integrative policy approach. *Environmental Research Letters* **2018**, *13* (12). DOI: 10.1088/1748-9326/aaeaa2.
(13) BCER. Data Center. British Columbia Energy Regulator. Available: https://www.bc-er.ca/data-reports/data-centre/ (Accessed 20 April 2023). **2023**.
(14) Lemprière, T. C.; Kurz, W. A.; Hogg, E. H.; Schmoll, C.; Rampley, G. J.; Yemshanov, D.; McKenney, D. W.; Gilsenan, R.; Beatch, A.; Blain, D.; et al. Canadian boreal forests and climate change mitigation. *Environmental Reviews* **2013**, *21* (4), 293-321. DOI: 10.1139/er-2013-0039.
(15) Seedre, M.; Shrestha, B. M.; Chen, H. Y. H.; Colombo, S.; Jõgiste, K. Carbon dynamics of North American boreal forest after stand replacing wildfire and clearcut logging. *Journal of Forest Research* **2017**, *16* (3), 168-183. DOI: 10.1007/s10310-011-0264-7.
(16) Zha, T. S.; Barr, A. G.; Bernier, P. Y.; Lavigne, M. B.; Trofymow, J. A.; Amiro, B. D.; Arain, M. A.; Bhatti, J. S.; Black, T. A.; Margolis, H. A.; et al. Gross and aboveground net primary production at Canadian forest carbon flux sites. *Agricultural and Forest Meteorology* **2013**, *174-175*, 54-64. DOI: 10.1016/j.agrformet.2013.02.004.
(17) Deloitte LLP. *Energy, oil, and gas price forecast. Available: https://www2.deloitte.com/ca/en/pages/resource-evaluation-and-advisory/topics/deloitte-canadian-price-forecast.html (Accessed 22 May 2023)*; 2023.
(18) Lyon, D. R.; Hmiel, B.; Gautam, R.; Omara, M.; Roberts, K. A.; Barkley, Z. R.; Davis, K. J.; Miles, N. L.; Monteiro, V. C.; Richardson, S. J.; et al. Concurrent variation in oil and gas methane emissions and oil price during the COVID-19 pandemic. *Atmospheric Chemistry and Physics* **2021**, *21* (9), 6605-6626. DOI: 10.5194/acp-21-6605-2021.
(19) BC Ministry of Environment and Climate Change Strategy. *Provincial greenhouse gas emissions inventory 1990-2021. Government of British Columbia. Available: https://www2.gov.bc.ca/gov/content/environment/climate-change/data/provincial-inventory (Accessed 17 November 2023)*; 2023.
(20) BC Government. *Natural Gas & Oil Statistics. Government of British Columbia. Available: https://www2.gov.bc.ca/gov/content/industry/natural-gas-oil/statistics (Accessed 20 April 2023);* 2023.

(21) US EPA. *US Environmentally-Extended Input-Output (USEEIO) Technical Content. US Environmental Protection Agency. Available: https://www.epa.gov/land-research/us-environmentally-extended-input-output-useeio-technical-content (Accessed 5 October 2023)*; 2023.
(22) Yatsenko, O.; Nitsenko, V.; Tananaiko, T.; Szetela, B.; Kobylianska, A. Trade and economic integration dominants in North America countries' interaction. *Journal of International Studies* **2019**, *12* (3), 277-293. DOI: 10.14254/2071-8330.2019/12-3/22.
(23) BCER. *Kermit. British Columbia Energy Regulator. Available: https://www.bc-er.ca/energy-professionals/online-systems/kermit/ (Accessed 27 April 2023)*; 2023.
(24) BCER. eLibrary. British Columbia Energy Regulator. Available: https://www.bc-er.ca/energy-professionals/online-systems/elibrary/ (Accessed 20 April 2023). **2023**.

(25) Mallapragada, D. S.; Reyes-Bastida, E.; Roberto, F.; McElroy, E. M.; Veskovic, D.; Laurenzi, I. J. Life cycle greenhouse gas emissions and freshwater consumption of liquefied Marcellus shale gas used for international power generation. *Journal of Cleaner Production* **2018**, *205*, 672-680. DOI: 10.1016/j.jclepro.2018.09.111.
(26) Liu, R. E.; Ravikumar, A. P.; Bi, X. T.; Zhang, S.; Nie, Y.; Brandt, A.; Bergerson, J. A. Greenhouse Gas Emissions of Western Canadian Natural Gas: Proposed Emissions Tracking for Life Cycle Modeling. *Environmental Science & Technology* **2021**, *55* (14), 9711-9720. DOI: 10.1021/acs.est.0c06353.
(27) Nie, Y.; Zhang, S.; Liu, R. E.; Roda-Stuart, D. J.; Ravikumar, A. P.; Bradley, A.; Masnadi, M. S.; Brandt, A. R.; Bergerson, J.; Bi, X. T. Greenhouse-gas emissions of Canadian liquefied natural gas for use in China: Comparison and synthesis of three independent life cycle assessments. *Journal of Cleaner Production* **2020**, *258*. DOI: 10.1016/j.jclepro.2020.120701.
(28) Stuckman, M. Y.; Lopano, C. L.; Berry, S. M.; Hakala, J. A. Geochemical solid characterization of drill cuttings, core and drilling mud from Marcellus Shale Energy development. *Journal of Natural Gas Science and Engineering* **2019**, *68*. DOI: 10.1016/j.jngse.2019.102922.
(29) Panther Drilling. *Rig 3 Details. Panther Drilling Corporation Inc. Available: http://pantherdrilling.ca/rig-3 (Accessed 4 April 2023)*; 2023.
(30) US EPA. *AP-42: Compilation of Air Emissions Factors. United States Environmental Protection Agency. Available: https://www.epa.gov/air-emissions-factors-and-quantification/ap-42-compilation-air-emissions-factors (Accessed 2 May 2023)*; 2023.
(31) BCER. *GIS Open Data Portal. British Columbia Energy Regulator. Available: https://data-bc-er.opendata.arcgis.com/ (Accessed 27 April 2023)*; 2023.
(32) Enbridge. *Informational Postings. Available: https://noms.wei-pipeline.com/info/ (Accessed 18 October 2023)*; 2021.
(33) Modern West Advisory Inc. *Life-Cycle Analysis of Canadian Natural Gas. Available: https://www.eralberta.ca/wp-content/uploads/2022/04/Modern-West-Advisory-Final-Outcomes-Report-Non-Confidential.pdf (Accessed 23 March 2023)*; 2021.
(34) BCOGC. *British Columbia's 2021 Oil and Gas Reserves and Production Report. BC Oil and Gas Commission. Available: https://www.bc-er.ca/data-reports/reports/ (Accessed 30 March 2023)*; 2022.
(35) Skone, T. J.; Schivley, G.; Jamieson, M.; Marriott, J.; Cooney, G.; Littlefield, J.; Mutchek, M.; Krynock, M.; Shih, C. *Life Cycle Analysis: Natural Gas Combined Cycle (NGCC) Power Plants. DOE/NETL-2018/1890. Available: https://www.osti.gov/biblio/1562914 (Accessed 19 October 2022)*; National Energy Technology Laboratory, 2018.
(36) Jordaan, S. M.; Heath, G. A.; Macknick, J.; Bush, B. W.; Mohammadi, E.; Ben-Horin, D.; Urrea, V.; Marceau, D. Understanding the life cycle surface land requirements of natural gas-fired electricity. *Nature Energy* **2017**, *2* (10), 804-812. DOI: 10.1038/s41560-017-0004-0.
(37) BCOGC. *Well Decommissioning Guidelines. VERSION 1.2: November 2022. BC Oil and Gas Commission. Available: https://www.bc-er.ca/files/operations-documentation/Well-Decommissioning/Well-Decommissioning-Guidelines.pdf (Accessed 28 April 2023)*; 2022.
(38) Tyner, D. R.; Johnson, M. R. Where the Methane Is-Insights from Novel Airborne LiDAR Measurements Combined with Ground Survey Data. *Environmental Science & Technology* **2021**, *55* (14), 9773-9783. DOI: 10.1021/acs.est.1c01572.

(39) Baray, S.; Jacob, D. J.; Maasakkers, J. D.; Sheng, J.-X.; Sulprizio, M. P.; Jones, D. B. A.; Bloom, A. A.; McLaren, R. Estimating 2010–2015 anthropogenic and natural methane emissions in Canada using ECCC surface and GOSAT satellite observations. *Atmospheric Chemistry and Physics* **2021**, *21* (23), 18101-18121. DOI: 10.5194/acp-21-18101-2021.
(40) Atherton, E.; Risk, D.; Fougère, C.; Lavoie, M.; Marshall, A.; Werring, J.; Williams, J. P.; Minions, C. Mobile measurement of methane emissions from natural gas developments in northeastern British Columbia, Canada. *Atmospheric Chemistry and Physics* **2017**, *17* (20), 12405-12420. DOI: 10.5194/acp-17-12405-2017.
(41) MacKay, K.; Lavoie, M.; Bourlon, E.; Atherton, E.; O'Connell, E.; Baillie, J.; Fougere, C.; Risk, D. Methane emissions from upstream oil and gas production in Canada are underestimated. *Scientific Reports* **2021**, *11* (1), 8041. DOI: 10.1038/s41598-021-87610-3.
(42) BC Ministry of Environment and Climate Change Strategy. *Provincial greenhouse gas emissions inventory 1990-2020. Government of British Columbia. Available: https://www2.gov.bc.ca/gov/content/environment/climate-change/data/provincial-inventory (Accessed 21 February 2023)*; 2022.
(43) BC Ministry of Environment and Climate Change Strategy. *Provincial greenhouse gas emissions inventory 1990-2019. Available: https://www2.gov.bc.ca/gov/content/environment/climate-change/data/provincial-inventory (Accessed 27 October 2021)*; 2021.
(44) *QGIS (Version 3.34.0-Prizren) [Computer program]*; 2023.
(45) Shen, L.; Gautam, R.; Omara, M.; Zavala-Araiza, D.; Maasakkers, J. D.; Scarpelli, T. R.; Lorente, A.; Lyon, D.; Sheng, J.; Varon, D. J.; et al. Satellite quantification of oil and natural gas methane emissions in the US and Canada including contributions from individual basins. *Atmospheric Chemistry and Physics* **2022**, *22* (17), 11203-11215. DOI: 10.5194/acp-22-11203-2022.
(46) BC Government. *Clean BC. Roadmap to 2030. Available: https://www2.gov.bc.ca/assets/gov/environment/climate-change/action/cleanbc/cleanbc_roadmap_2030.pdf (Accessed 18 October 2022)*; 2021.
(47) Canada Energy Regulator. *Energy conversion calculator. Available: https://apps.cer-rec.gc.ca/Conversion/calculator-calculatrice.aspx?GoCTemplateCulture=en-CA*; 2016.
(48) Yin, W.; Venderbosch, R. H.; Heeres, H. J. Recent developments in the catalytic hydrotreatment of pyrolysis liquids. In *Direct Thermochemical Liquefaction for Energy Applications*, Elsevier, 2018; pp 249-292.
(49) Gas Processors Suppliers Association. *Engineering Data Book. 12th ed. FPS.*; Gas Processors Suppliers Association, 2004.
(50) ECCC. Canada's Air Pollutant Emissions Inventory. Environment and Climate Change Canada. Available: https://data.ec.gc.ca/data/substances/monitor/canada-s-air-pollutant-emissions-inventory/APEI_Tables_Canada_Provinces_Territories/?lang (Accessed 2 May 2023). **2023**.
(51) Statistics Canada. Supply and demand of primary and secondary energy in natural units. Table: 25-10-0030-01. Available: https://doi.org/10.25318/2510003001-eng (Accessed 11 May 2023). 2022.
(52) BC Ministry of Environment and Climate Change Strategy. *Electricity emission intensity factors for grid-connected entities. Available:*

https://www2.gov.bc.ca/gov/content/environment/climate-change/industry/reporting/quantify/electricity; 2022.
(53) Government of Canada. *Multi-Sector Air Pollutants Regulations. Canadian Environmental Protection Act. Available: https://gazette.gc.ca/rp-pr/p2/2016/2016-06-29/html/sor-dors151-eng.html (Accessed 16 October 2023)*; 2016.
(54) *Simapro (Version 9.5.0.1) [Computer program]*; 2023.
(55) Chemical Engineering. *Chemical Engineering Plant Cost Index. Available: https://www.chemengonline.com/pci-home*; 2021.
(56) BCER. Email correspondence to Ryan Cownden (27 October). RE: Incident Release Inquiry. **2023**.
(57) AESO. Historical Generation Data (CSD). Alberta Electric System Operator. Available: https://www.aeso.ca/market/market-and-system-reporting/data-requests/historical-generation-data/ (Accessed 7 July 2023). **2023**.
(58) US EPA. Clean Air Markets Program Data. United States Environmental Protection Agency. Available: https://campd.epa.gov/data/custom-data-download (Accessed 24 August 2023). **2023**.
(59) UKBEIS. *Start-up and Shut-down times of power CCUS facilities. United Kingdom Department for Business, Energy & Industrial Strategy. BEIS research paper number 2020/031. Available: https://www.gov.uk/government/publications/start-up-and-shut-down-times-of-power-carbon-capture-usage-and-storage-ccus-facilities (Accessed 6 Apr 2023)*; 2020.
(60) Kehlhofer, R.; Rukes, B.; Hannemann, F.; Stirnimann, F. *Combined-Cycle Gas and Steam Turbine Power Plants. 3rd edn. Tulsa, OK, USA: Pennwell Corporation.*; 2019.
(61) Environmental Consulting & Technology Inc. *REVISED PLAN APPROVAL APPLICATION FOR THE HILL TOP ENERGY CENTER COMBINED-CYCLE PROJECT, Cumberland Township, Greene County, Pennsylvania. Revision I. ECT No. 150533-0500. July 2017. Available: https://www.dep.pa.gov/About/Regional/SouthwestRegion/Community%20Information/Pages/Hill-Top-Energy-Center.aspx (Accessed 24 Aug 2023)*; 2017.
(62) Environmental Resources Management. *ESC Harrison County Power, LLC Air Permit Application, Combined-Cycle Power Plant Project, Clarksburg, Harrison County, West Virginia. Available: http://www.dep.wv.gov/daq/Documents/December%202016%20Applications/033-00264_APPL_R14-0036.pdf (Accessed 7 August 2023).* 2016.
(63) Schmitt, T.; Leptinsky, S.; Turner, M.; Zoelle, A.; Woods, M.; Shultz, T.; James, R. *Cost And Performance Baseline for Fossil Energy Plants Volume 1: Bituminous Coal and Natural Gas to Electricity. National Energy Technology Laboratory. Available: https://doi.org/10.2172/1893822. (Accessed 9 February 2023)*; 2022.
(64) IEAGHG. *Update Techno-Economic Benchmarks for Fossil Fuel-Fired Power Plants with CO_2 Capture. IEAGHG Technical Report 2020-07.* ; 2020.
(65) Spitz, T.; González Díaz, A.; Chalmers, H.; Lucquiaud, M. Operating flexibility of natural gas combined cycle power plant integrated with post-combustion capture. *International Journal of Greenhouse Gas Control* **2019**, *88*, 92-108. DOI: 10.1016/j.ijggc.2019.04.023.
(66) Buvik, V.; Høisæter, K. K.; Vevelstad, S. J.; Knuutila, H. K. A review of degradation and emissions in post-combustion CO2 capture pilot plants. *International Journal of Greenhouse Gas Control* **2021**, *106*. DOI: 10.1016/j.ijggc.2020.103246.

(67) Morken, A. K.; Pedersen, S.; Nesse, S. O.; Flø, N. E.; Johnsen, K.; Feste, J. K.; de Cazenove, T.; Faramarzi, L.; Vernstad, K. CO2 capture with monoethanolamine: Solvent management and environmental impacts during long term operation at the Technology Centre Mongstad (TCM). *International Journal of Greenhouse Gas Control* **2019**, *82*, 175-183. DOI: 10.1016/j.ijggc.2018.12.018.
(68) Hajny, K. D.; Salmon, O. E.; Rudek, J.; Lyon, D. R.; Stuff, A. A.; Stirm, B. H.; Kaeser, R.; Floerchinger, C. R.; Conley, S.; Smith, M. L.; Shepson, P. B. Observations of Methane Emissions from Natural Gas-Fired Power Plants. *Environ Sci Technol* **2019**, *53* (15), 8976-8984. DOI: 10.1021/acs.est.9b01875.
(69) Bechtel National Inc. *Front-End Engineering Design (FEED) Study for a Carbon Capture Plant Retrofit to a Natural Gas-Fired Gas Turbine Combined Cycle Power Plant (2x2x1 Duct-Fired 758-MWe Facility with F Class Turbines). doi:10.2172/1836563*; 2022.
(70) *Aspen Process Economic Analyzer, Version 11 (Build 38.0.0.4079)*; 2019.
(71) Pavri, R.; Moore, G. D. *Gas Turbine Emissions and Control. General Electric Power Systems Report GER-4211. Available: https://www.ge.com/content/dam/gepower-new/global/en_US/downloads/gas-new-site/resources/reference/ger-4211-gas-turbine-emissions-and-control.pdf (Accessed 7 July 2023)*; 2001.
(72) Bender, B. *Lean Pre-Mixed Combustion. In: Gas Turbine Handbook. National Energy Technology Laboratory. Available: https://netl.doe.gov/carbon-management/turbines/handbook (Accessed 7 July 2023).* 2007.
(73) Dunn-Rankin, D. *Lean combustion technology and control*; Academic Press, 2008.
(74) Barbera, E.; Mio, A.; Massi Pavan, A.; Bertucco, A.; Fermeglia, M. Fuelling power plants by natural gas: An analysis of energy efficiency, economical aspects and environmental footprint based on detailed process simulation of the whole carbon capture and storage system. *Energy Conversion and Management* **2022**, *252*. DOI: 10.1016/j.enconman.2021.115072.
(75) Fadeyi, S.; Arafat, H. A.; Abu-Zahra, M. R. M. Life cycle assessment of natural gas combined cycle integrated with CO2 post combustion capture using chemical solvent. *International Journal of Greenhouse Gas Control* **2013**, *19*, 441-452. DOI: 10.1016/j.ijggc.2013.10.008.
(76) Holloway, S.; Karimjee, A.; Akai, M.; Pipatti, R.; Rypdal, K. *IPCC Guidelines for National Greenhouse Gas Inventories. Volume 2: Energy. Chapter 5: Carbon Dioxide Transport, Injection and Geological Storage. Available: https://www.ipcc-nggip.iges.or.jp/public/2006gl/pdf/2_Volume2/V2_5_Ch5_CCS.pdf (Accessed 2 March 2022)*; 2006.
(77) Preston, C.; Monea, M.; Jazrawi, W.; Brown, K.; Whittaker, S.; White, D.; Law, D.; Chalaturnyk, R.; Rostron, B. IEA GHG Weyburn CO2 monitoring and storage project. *Fuel Processing Technology* **2005**, *86* (14-15), 1547-1568. DOI: 10.1016/j.fuproc.2005.01.019.
(78) White, D. Seismic characterization and time-lapse imaging during seven years of CO2 flood in the Weyburn field, Saskatchewan, Canada. *International Journal of Greenhouse Gas Control* **2013**, *16*, S78-S94. DOI: 10.1016/j.ijggc.2013.02.006.
(79) Brown, K.; Whittaker, S.; Wilson, M.; Srisang, W.; Smithson, H.; Tontiwachwuthikul, P. The history and development of the IEA GHG Weyburn-Midale CO2 Monitoring and Storage Project in Saskatchewan, Canada (the world largest CO2 for EOR and CCS program). *Petroleum* **2017**, *3* (1), 3-9. DOI: 10.1016/j.petlm.2016.12.002.

(80) Furre, A.-K.; Eiken, O.; Alnes, H.; Vevatne, J. N.; Kiær, A. F. 20 Years of Monitoring CO2-injection at Sleipner. *Energy Procedia* **2017**, *114*, 3916-3926. DOI: 10.1016/j.egypro.2017.03.1523.
(81) Huijbregts, M. A. J.; Steinmann, Z. J. N.; Elshout, P. M. F.; Stam, G.; Verones, F.; Vieira, M.; Zijp, M.; Hollander, A.; van Zelm, R. ReCiPe2016: a harmonised life cycle impact assessment method at midpoint and endpoint level. *The International Journal of Life Cycle Assessment* **2016**, *22* (2), 138-147. DOI: 10.1007/s11367-016-1246-y.
(82) *Aspen Hysys, Version 11 [Computer program]*; 2019.
(83) TC Energy. Gas Quality Specifications. Available: http://www.tccustomerexpress.com/docs/Gas_Quality_Specifications_Fact_Sheet.pdf (Accessed 4 June 2023). 2022.
(84) Pembina Pipeline Corporation. NEBC, BC LIGHT, BLUEBERRY & BOUNDARY LAKE GATHERING PETROLEUM SPECIFICATIONS. Available: https://www.pembina.com/getmedia/925a342d-f09e-480f-be7c-1c0b989c9e89/NEBC-BCPL-Condensate-Specification-May-2022.pdf (Accessed 4 June 2023). 2022.
(85) Danaci, D.; Bui, M.; Petit, C.; Mac Dowell, N. En Route to Zero Emissions for Power and Industry with Amine-Based Post-combustion Capture. *Environmental Science & Technology* **2021**, *55* (15), 10619-10632. DOI: 10.1021/acs.est.0c07261.
(86) Michailos, S.; Gibbins, J. A Modelling Study of Post-Combustion Capture Plant Process Conditions to Facilitate 95–99% CO2 Capture Levels From Gas Turbine Flue Gases. *Frontiers in Energy Research* **2022**, *10*. DOI: 10.3389/fenrg.2022.866838.
